# *Syn*-selective silicon Mukaiyama-type aldol reactions of (pentafluoro-λ^6^-sulfanyl)acetic acid esters with aldehydes

**DOI:** 10.3762/bjoc.14.25

**Published:** 2018-02-08

**Authors:** Anna-Lena Dreier, Andrej V Matsnev, Joseph S Thrasher, Günter Haufe

**Affiliations:** 1 Organisch-Chemisches Institut, Universität Münster, Corrensstraße 40, 48149 Münster, Germany; 2 Department of Chemistry, Advanced Materials Research Laboratory, Clemson University, 91 Technology Drive, Anderson, South Carolina 29625, United States of America; 3 Cells-in-Motion Cluster of Excellence, Universität Münster, Waldeyerstraße 15, 48149 Münster, Germany

**Keywords:** aldol reaction, ester enolate, fluorine, SF_5_ compounds, stereochemistry

## Abstract

Aldol reactions belong to the most frequently used C–C bond forming transformations utilized particularly for the construction of complex structures. The selectivity of these reactions depends on the geometry of the intermediate enolates. Here, we have reacted octyl pentafluoro-λ^6^-sulfanylacetate with substituted benzaldehydes and acetaldehyde under the conditions of the silicon-mediated Mukaiyama aldol reaction. The transformations proceeded with high diastereoselectivity. In case of benzaldehydes with electron-withdrawing substituents in the *para*-position, *syn*-α-SF_5_-β-hydroxyalkanoic acid esters were produced. The reaction was also successful with *meta*-substituted benzaldehydes and *o*-fluorobenzaldehyde. In contrast, *p*-methyl-, *p*-methoxy-, and *p*-ethoxybenzaldehydes led selectively to aldol condensation products with (*E*)-configured double bonds in 30–40% yields. In preliminary experiments with an SF_5_-substituted acetic acid morpholide and *p*-nitrobenzaldehyde, a low amount of an aldol product was formed under similar conditions.

## Introduction

The classical acid- or base-catalyzed directed cross aldol reaction of an aldehyde and an enolizable second carbonyl compound is one of the most powerful and reliable carbon–carbon bond-forming transformations in organic synthesis applied most successfully for the construction of natural products and their analogs [1–2]. Later this type of reaction was extended to enolized carboxylic acid derivatives, particularly to silylated ketene acetals, as reaction partners for carbonyl active compounds [3–5]. Mild and highly selective reaction conditions could be developed in parallel with the progress in understanding the mechanism of these transformations [6–9]. Also, aldol reactions with fluorinated substrates, particularly with trifluoromethyl-containing ones, were investigated [10–13].

In recent years, the pentafluoro-λ^6^-sulfanyl (SF_5_) substituent has come into the focus of chemists because of the remarkable effects of this substituent on the physical and chemical properties of compounds, which are important for agricultural and medicinal chemistry as well as for materials sciences. While aromatic [14–15] and heteroaromatic [16–17] SF_5_ compounds have become readily available and many applications have been described [18–19], the chemistry of aliphatic analogs is still underdeveloped [20]. Generally, the incorporation of SF_5_ groups into aliphatic positions is based on radical addition of SF_5_X (X = Cl, Br, SF_5_) across π-bonds. The unconventional conditions usually required were overcome by Dolbier’s elegant triethylborane initiation [21]. Recently, the radical arylation of a SF_5_-substituted alkene was realized in order to gain access to SF_5_-containing dihydrobenzofurans and indolines [22]. There are not many transformations of aliphatic SF_5_ compounds described in the literature. Among them are the preparation and derivatization of SF_5_-aldehydes [23], Diels–Alder reactions [24–26], the “click reaction” of SF_5_-acetylenes with azides to form triazoles [27], and 1,3-dipolar cycloadditions of azomethine ylides with pentafluoro-λ^6^-sulfanyl-substituted acrylic esters and amides [28].

A couple of years ago, we became interested in SF_5_-substituted ester enolates as reaction intermediates. Thus, in 2016 we reported a highly *anti*-selective aldol addition of SF_5_-substituted acetic ester-based boron enolates to aromatic and aliphatic aldehydes to form *anti*-2-pentafluoro-λ^6^-sulfanyl-3-hydroxyalkyl-carboxylic acid esters [29]. Quite similar results were published independently by Carreira et al. a couple of weeks before us [30].

Recently, we discovered that silylated enolates can be formed as mixtures of (*E*)- and (*Z*)-isomers from SF_5_-substituted acetic acid esters of aliphatic terminal allylic alcohols at low temperature. At slightly elevated temperature, the latter diastereomers are transformed to γ,δ-unsaturated α-pentafluoro-λ^6^-sulfanyl alkanoic acids in an Ireland–Claisen rearrangement [31]. Under slightly modified conditions, this type of rearrangement was also successful for SF_5_-substituted acetic esters of cinnamyl alcohols [32].

Herein we describe our results [33] of highly *syn*-diastereoselective silicon Mukaiyama-type aldol reactions of SF_5_-acetic acid esters with different aldehydes. While our research was well underway, Ponomarenko and Röschenthaler et al. reported similar Ti(IV)-mediated aldol reactions proceeding via titanium enolates and some succeeding or competing reactions of intermediates or products [34].

## Results and Discussion

**Scheme 1:**
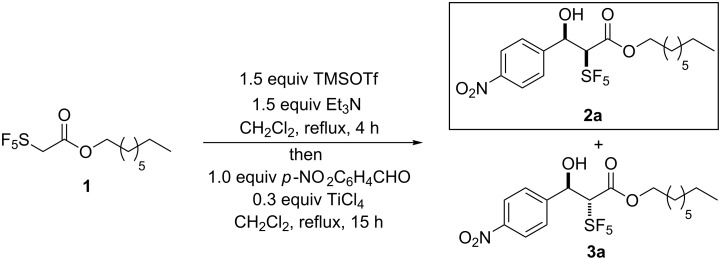
Silicon-mediated Mukaiyama-type aldol reaction of octyl 2-(pentafluoro-λ^6^-sulfanyl)acetate (**1**) with *p*-nitrobenzaldehyde.

As a model for our investigations we chose the reaction of the less volatile octyl 2-(pentafluoro-λ^6^-sulfanyl)acetate (**1**) with *p*-nitrobenzaldehyde. Analogously to a protocol used by Ishihara et al. [35] for an Evans aldol reaction of trifluoropropanoic amides, we refluxed 1 equiv of ester **1** with 1.5 equiv trimethylsilyl trifluoromethanesulfonate (TMSOTf) and 1.5 equiv triethylamine (Et_3_N) in dichloromethane for 4 hours. Then the mixture was cooled down to 0 °C and 1 equiv of *p*-nitrobenzaldehyde and 0.3 equiv of TiCl_4_ were added under stirring. Stirring at room temperature was continued for 15 hours. Then the reaction was quenched by the addition of ice-water. After work-up 22% of the aldol addition products were formed in a *syn/anti*-ratio of 97:3 as determined by ^19^F NMR spectroscopy (Scheme 1). Subsequently, the reaction conditions were optimized (Table 1).

**Table 1:** Optimization of the reaction conditions for the silicon Mukaiyama-type aldol reaction of ester **1** with *p*-nitrobenzaldehyde.

Entry	TMSOTf [equiv]	Et_3_N [equiv]	Lewis acid [equiv]	Temp.	Time [h]	Yield **2** + **3** [%]^a^ (**2**:**3***-*ratio)

1	1.5	1.5	0.3 TiCl_4_	rt	15	22 (93:7)
2	1.5	1.5	0.3 TiCl_4_	reflux	15	53 (93:7)
3	1.5	1.5	0.3 TiCl_4_	reflux	3 days	40 (78:22)
4	1.2	1.5	0.3 TiCl_4_	reflux	15	61 (73:27)
5	1.5	3.0	0.3 TiCl_4_	reflux	15	0
6	1.5	1.5	1.0 TiCl_4_	reflux	15	67 (81:19)
7	1.5	1.5	0.3 BF_3_·OEt_2_	reflux	15	0
8	1.5	1.5	1.0 BF_3_·OEt_2_	reflux	15	0
9	1.5	1.5	1.3 BF_3_·OEt_2_	reflux	15	0

^a^Determined by ^19^F NMR spectroscopy of the crude product mixture.

Elevation of the reaction temperature (15 h reflux) led to an increase of the yield, while the *syn/anti*-ratio was not changed (Table 1, entry 2). Elongation of the reaction time resulted in the formation of more side products, drop of aldol products’ yield and selectivity (Table 1, entry 3). A lower amount of TMSOTf (1.2 equiv) led to increased yield but lower selectivity (Table 1, entry 4). Application of excess Et_3_N delivered a complex mixture of products including fluorine-free ones. Only traces of the aldol products were detected (Table 1, entry 5). Increasing the amount of TiCl_4_ to 1.0 equiv resulted in an increased yield (67%), but lower selectivity (81:19, Table 1, entry 6). All attempts to catalyze the reaction with BF_3_·OEt_2_ as a Lewis acid failed. Aldol products were not found (Table 1, entries 7–9). Thus, the conditions of entry 2 presented the best compromise regarding the yield and selectivity. Therefore, these conditions were used for the reactions of other aromatic and aliphatic aldehydes (Table 2).

**Table 2:** Results of reactions of ester **1** with different aldehydes.

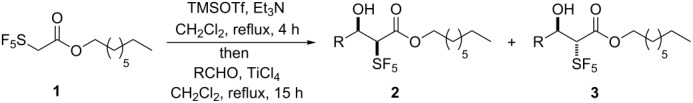				

Entry	Compounds	R	Yield **2** + **3** [%]^a^	**2** : **3** ratio^b^

1	**a**	*p*-NO_2_-C_6_H_4_	53 (40)	93:7 (99:1)
2	**b**	C_6_H_5_	44 (30)	86:14 (93:7)
3	**c**	*p*-F-C_6_H_4_	44 (37)	86:14 (91:9)
4	**d**	*p*-Cl-C_6_H_4_	38 (19)	81:19 (87:13)
5	**e**	*p*-Br-C_6_H_4_	39 (19)	83:17 (86:14)
6	**f**	*p*-SF_5_-C_6_H_4_	42 (22)	95:5 (95:5)
7	**g**	*p*-CH_3_-C_6_H_4_	0	–
8	**h**	*p*-CH_3_O-C_6_H_4_	0	–
9	**i**	*p*-C_2_H_5_O-C_6_H_4_	0	–
10	**j**	*m*-NO_2_*-*C_6_H_4_	61 (44)	90:10 (90:10)
11	**k**	*m*-CH_3_*-*C_6_H_4_	57 (41)	73:27 (84:16)
12	**l**	*m*-CH_3_O*-*C_6_H_4_	35 (26)	88:12 (99:1)
13	**m**	*o*-F-C_6_H_4_	69 (56)	83:17 (90:10)
14	**n**	*o*-Br-C_6_H_4_	0	–
15	**o**	2,6-Cl_2_-C_6_H_3_	0	–
16	**p**	2,4-(NO_2_)_2_-C_6_H_3_	0	–
17	**q**	CH_3_	58 (37)	87:13 (89:11)
18	**r**	*cyclo-*C_6_H_11_	0	–
19	**s**	CH_2_=CH	0	–
20	**t**	CH_3_-CH=CH	0	–

^a^Combined yield determined by ^19^F NMR spectroscopy of the crude mixture, isolated yield after repeated chromatography in parentheses; ^b^*syn/anti-*ratio of the crude product, ratio after chromatographic purification in parentheses determined by ^19^F NMR spectroscopy.

Table 2 shows that benzaldehyde and five of its derivatives substituted with electron withdrawing groups in *para*-position gave the desired aldol addition products in fair yields and with diastereoselectivities between 81:19 and 95:5 in favor of the *syn*-products (Table 2, entries 1–6). In contrast, from the reactions of **1** with benzaldehydes bearing electron-donating substituents (methyl-, methoxy-, ethoxy-) no aldol addition products could be isolated (Table 2, entries 7–9), but the aldol condensation products **4g–i** were isolated in fair yields (see below). On the other hand, *meta*-substituted benzaldehydes gave mainly the *syn-*aldolates **2j–l** and small amounts of the *anti*-isomers **3j–l** regardless of the electronic nature of the aryl substituent (Table 2, entries 10–12). 2-Fluorobenzaldehyde gave the highest yield (69%) and 83:17 diastereoselectivity (Table 2, entry 13), while 2-bromo-, 2,6-dichloro- and 2,4-dinitrobenzaldehydes failed to give any aldol products (Table 2, entries 14–16). Besides the starting materials, only minor amounts of SF_5_-containing side products of unknown structure were detected in the ^19^F NMR spectra of the crude product mixtures. Among the saturated and unsaturated aliphatic aldehydes, only acetaldehyde gave the expected aldolates (58% yield, ratio 87:13, Table 2, entry 17), while cyclohexane carbaldehyde, acrolein, and crotonaldehyde did not give any aldol products (Table 2, entries 18–20).

During the aldol addition, two stereocenters were formed giving either the *syn*-**2** or the *anti*-isomers **3**. In all reactions one of the diastereomers was formed in large excess. In most cases, this isomer was isolated in an almost pure form after repeated chromatography, which explains the low isolated yields.

**Figure 1:**
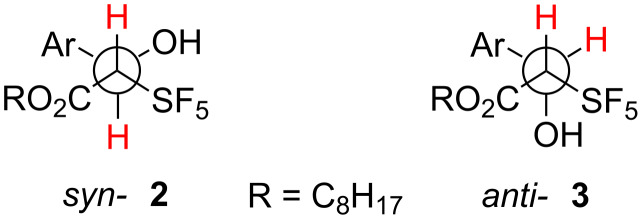
Newman projections of the *syn*- and the *anti*-diastereomeric aldol addition products.

Unfortunately, all products are oils and could not be crystallized. Thus, in order to determine the relative stereochemistry, we analyzed the vicinal coupling constants of the protons attached to the stereocenters. In the *syn*-isomers **2** these protons are *anti* to each other, while in the *anti*-isomers **3** these protons are in a *syn*-arrangement (Figure 1). Such an arrangement was found in the crystalline state of an *anti*-aldol product we obtained as a result of a boron-mediated aldol reaction of the benzyl ester analog of compound **1** with benzaldehyde [29]. This product and a couple of its aryl-substituted analogs exhibited coupling constants of 2.4–4.0 Hz. The products **3** produced as minor compounds in the present study had coupling constants between 3.3 and 4.0 Hz, which is typical for a gauche arrangement of the coupling nuclei according to the Karplus equation. Also the other NMR data agree with those found for authentic *anti*-aldol addition products. Thus, the minor products of the silicon Mukaiyama aldol reactions are the *anti*-isomers **3**.

On the other hand, the vicinal coupling constants of the major products were found between 8.5 Hz for the *p*-nitro derivative **2a** to about 9.4 Hz for derivatives **2b–e** with less electron-withdrawing substituents in *para-* and *meta*-positions, showing that these protons are in *anti*-position to each other as shown for compounds **2** in Figure 1. Thus, the major products are *syn*-isomers. Almost identical coupling constants and chemical shifts were also found for the aldol addition products of methyl SF_5_-acetate with benzaldehyde, *p*-nitro-, and *p*-methoxybenzaldehyde as described recently by Ponomarenko and Röschenthaler et al. [34].

**Scheme 2:**
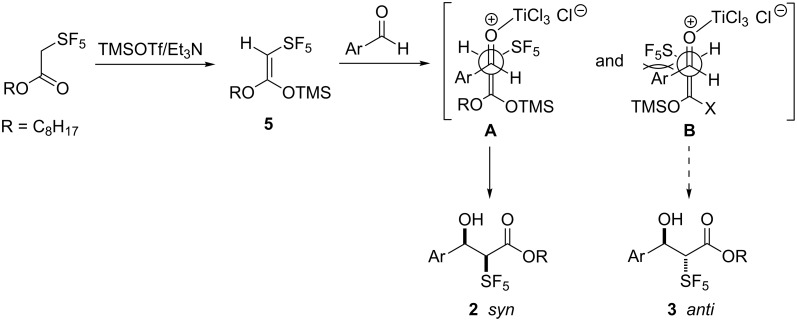
Mechanism of the formation of aldol addition products.

Considering our earlier results [31] on TMSOTf-mediated Claisen-type rearrangements of SF_5_-acetates of allyl alcohols, we favor the initial formation of (*Z*)-enolates (ketene silylacetals) **5** in the aldol reactions (Scheme 2).

From this enolate, two transition states **A** and **B** can be formed for the aldol reactions. **B** should be less favored due to the steric (and may be also electronic) repulsion of the aryl and the SF_5_ groups. Consequently, the *syn*-products resulting from transition state **A** are the major products of the aldol addition reactions.

**Scheme 3:**
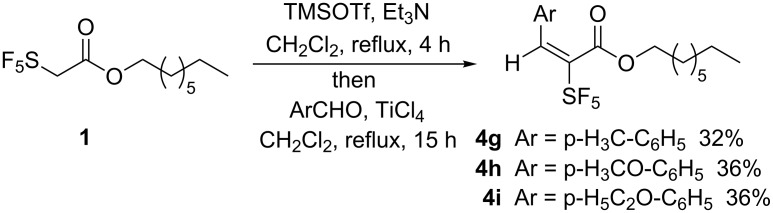
Formation of (*E*)-configured aldol condensation products.

As mentioned above, aldol addition products were not isolated from the reaction of **1** with electron rich *p*-methyl-, *p*-methoxy-, and *p*-ethoxybenzaldehydes. Here, aldol condensation products **4g–i** were obtained as single isomers after chromatography (Scheme 3).

In order to ascertain the configuration of our products, we performed a heteronuclear Overhauser effect (^1^H,^19^F-correlation spectrum (gHOESY), see Supporting Information File 1 for a copy of the spectrum). From this spectrum it becomes clear that the four equatorial fluorine atoms do interact with the vinylic proton. Thus, these atoms must be located in spatial proximity. This is possible only in the (*E*)-configuration of the double bond. A second proof for this configuration is the signal of the vinylic proton at δ = 7.41 ppm, which is a singlet. This was also found in the spectra of the analogous methyl esters, while for the corresponding (*Z*)-products a multiplet was identified, which is formed by ^4^*J*_H,F_ coupling with the four equatorial fluorine atoms of the SF_5_ group [34].

**Scheme 4:**
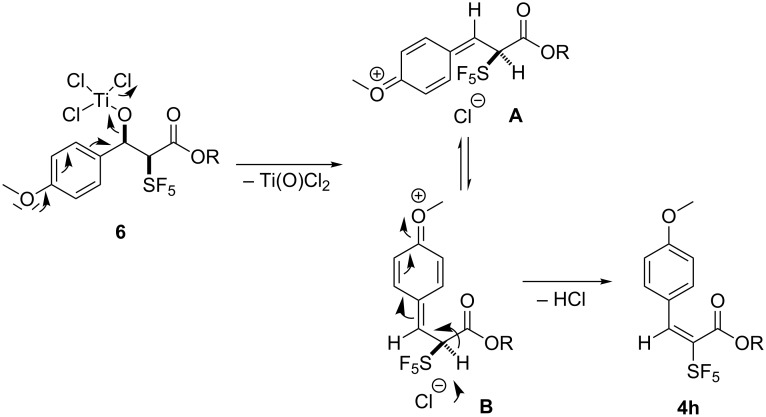
Anticipated mechanism of formation of aldol condensation products.

The formation of the condensation products might occur via two alternative mechanisms. Both variants depend on the initial formation of aldol addition products. These products could undergo acid catalyzed dehydration during aqueous work-up. Due to the presence of Lewis acids, which will be hydrolyzed, water would most probably eliminate via an intermediate benzyl cation, which would be stabilized by the electron-donating substituents in *p*-position. Consequently, the formation of a mixture of *E*/*Z*-isomers would be expected. Therefore, we were in favor of an alternative mechanism involving elimination subsequent to the addition step. According to Denmark’s mechanism [5] for the silicon Mukaiyama aldol reaction (see above), the nucleophilic attack of the silicon enolate (in our case the ketene silylacetal) at the TiCl_4_-activated aldehyde results in the formation of intermediate **6**. Under the influence of the electron-donating substituent, the elimination of titanium oxide dichloride (Ti(O)Cl_2_) is favored under liberation of a chloride. For the formed oxonium ion, two conformers **A** and **B** are possible due to free rotation around the single bond neighboring the SF_5_ group and the former benzylic carbon atom (Scheme 4).

Due to the possible repulsive interaction of the SF_5_ group with the quinoid ring in conformer **A**, conformer **B** should be favored. Assisted by the chloride, this species is deprotonated forming the condensation product **4h** with an (*E*)-configured double bond. Obviously, this formal dehydration is possible only in the presence of electron-donating *para*-substituents in the benzaldehyde. In order to evaluate this assumption, we performed the following NMR experiment: the ester **1** was dissolved in CD_2_Cl_2_ and TMSOTf and Et_3_N were successively added at room temperature. Then the mixture was refluxed for 4 hours, cooled down to ambient temperature, and *p*-anisaldehyde and TiCl_4_ were added. This mixture was heated to 40 °C in a sealed tube overnight and directly investigated by ^1^H and ^19^F NMR spectroscopy showing the formation of product **4h**. Thus, a concerted mechanism seems to be responsible for the exclusive formation of the (*E*)-configured products.

**Scheme 5:**
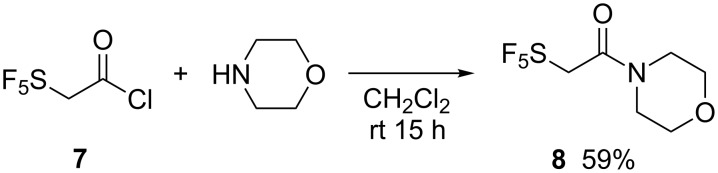
Synthesis of SF_5_-substituted acetmorpholide **8**.

Finally, we attempted to incorporate SF_5_-substituted acetamides into aldol additions with the intension of applying amides in Evans-type substrate directed asymmetric C–C-bond forming reactions. Therefore, 2-(pentafluoro-λ^6^-sulfanyl)acetic morpholide (**8**) was prepared by reaction of SF_5_-CH_2_C(O)Cl (**7**) [36–37] with morpholine (Scheme 5).

**Scheme 6:**
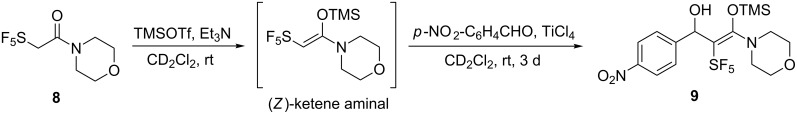
Intermediate formation of the (*Z*)-ketene aminal from morpholide **8** with TMSOTf/ Et_3_N and subsequent transformation to an aldol addition product **9** with *p*-nitrobenzaldehyde.

According to the general protocol, the morpholide **8** was treated with TMSOTf and Et_3_N and refluxed in CH_2_Cl_2_ for 4 hours. Then this mixture was treated with *p*-nitrobenzaldehyde and TiCl_4_ and refluxed in CH_2_Cl_2_ in a sealed tube for 15 hours. However, after aqueous work-up, the starting amide **8** and *p*-nitrobenzaldehyde were mainly recovered. A new SF_5_-containing compound was detected (^19^F NMR) in the crude product mixture, but we were unable to isolate this product. The following ^19^F NMR data were found δ = 57.7 ppm (dm, ^2^*J*_F,F_ = 147.6 Hz, 4F) and δ = 74.7 ppm (qn, ^2^*J*_F,F_ = 147.6 Hz, 1F), which are different from the starting material and from the corresponding carboxylic acid [36]. Therefore, we repeated the reaction in an NMR tube and treated the morpholide **8** with TMSOTf and Et_3_N in CD_2_Cl_2_ at room temperature. After a short period of time, the ^1^H and ^19^F NMR spectra showed the exclusive formation of the (*Z*)-ketene aminal. Signals of **8** were not found any more (Scheme 6).

The structure of this intermediate became evident from its NMR data (see Supporting Information File 1). In particular a quintet (^3^*J*_H,F_ = 7.0 Hz) at δ = 5.15 ppm in the ^1^H NMR spectrum is indicative of a vinylic proton. A quintet (^2^*J*_C,F_ = 19.4 Hz) at δ = 106.1 ppm in the ^13^C NMR spectrum can be assigned to the SF_5_-substituted carbon atom, and for the second carbon atom of the double bond a quintet (^3^*J*_C,F_ = 4.6 Hz) appears at δ = 156.9 ppm. From the poorly resolved multiplets at 91.6 ppm and 74.1 ppm in the ^19^F NMR spectrum one can reason that the SF_5_ group is attached to a double bond. Its (*Z*)-configuration was deduced from a ^1^H,^19^F-correlation spectrum (gHOESY) showing an interaction of the equatorial fluorine atoms of the SF_5_ group and protons of the TMS group (see Supporting Information File 1 for a copy of the spectrum). Consequently, these two substituents must be located at the same side of the double bond.

Subsequently, the reaction mixture was treated with *p*-nitrobenzaldehyde and TiCl_4_ and allowed to remain at room temperature for 3 days while new peaks appeared in NMR spectra. Besides signals of *p*-nitrobenzaldehyde, triethylamine hydrochloride, and several silicon compounds, the aforementioned additional signals were found. From two new doublets for two protons each of a 1,4-disubstituted benzene ring (δ = 8.32 and δ = 7.96), a multiplet for one proton at δ = 4.62 ppm, a broad singlet of one proton at δ = 6.29 ppm, and the two multiplets of the morpholine ring for four protons each at δ = 3.37 ppm and δ = 3.28 ppm, one can reason the formation of an aldol addition product **9**. This assignment is supported by a multiplet for one axial fluorine atom at δ = 75.39 ppm (*J* = 147 Hz) and a doublet (of multiplets) for four fluorine atoms at δ = 57.6 ppm (*J* = 147 Hz) in the ^19^F NMR spectra (overlapping with a doublet of another compound). Unfortunately, after work-up, we have not yet been able to isolate this product. Starting amide **8** was a major component of the crude reaction product.

In summary, the formation of the intermediate ketene aminal occurred very fast and was highly (*Z*)-selective. Unfortunately, the aldol product, which was formed after treatment with *p*-nitrobenzaldehyde, could not yet be isolated. Anyway, the preliminary results using morpholide **8** are promising. We expect that optimization of the reaction conditions and application of enantiopure Lewis acids or SF_5_-substituted acetamides bearing a chiral auxiliary will result in asymmetric aldol addition reactions in the future.

## Conclusion

From octyl SF_5_-acetate and TMSOTf/Et_3_N, a (*Z*)-enolate (ketene silylacetal) was preferentially formed as has already been shown in our earlier investigations. The C–C bond forming step proceeded preferably via a transition state with a *syn*-arrangement of the SF_5_ and OTMS groups resulting in the formation of *syn*-aldol products in case of aldehydes with electron-withdrawing substituents in the *para*-position or any substituents in the *meta*-position. In contrast, aldols derived from aldehydes with electron-donating substituents in the *para*-position were not stable under the reaction conditions. A formal Lewis acid (TiCl_4_)-assisted elimination of water produced the (*E)*-configured aldol condensation products. Preliminary results of formation of an aldol addition product from the reaction of an SF_5_-substituted acetmorpholide and *p*-nitrobenzaldehyde are promising, and successful asymmetric reactions may be expected.

## Supporting Information

File 1 General procedure, synthesis of the aldol products, spectroscopic data, and copies of ^1^H, ^13^C, and ^19^F NMR spectra.
