# Supplementary material for: Syn-selective silicon Mukaiyama-type aldol reactions of (pentafluoro-λ6-sulfanyl)acetic acid esters with aldehydes
Source: Beilstein J Org Chem. 2018 Feb 8;14:373–80. doi: 10.3762/bjoc.14.25 (PMC5815270; doi:10.3762/bjoc.14.25)

Supporting Information

for

***Syn*-selective silicon Mukaiyama-type aldol reactions of (pentafluoro-λ^6-sulfanyl)acetic acid esters with aldehydes**

Anna-Lena Dreier[1], Andrej V. Matsnev[2], Joseph S. Thrasher[2], and Günter Haufe[1,3]*

Address: [1]Organisch-Chemisches Institut, Universität Münster, Corrensstraße 40, 48149 Münster, Germany, [2]Department of Chemistry, Advanced Materials Research Laboratory, Clemson University, 91 Technology Drive, Anderson, South Carolina 29625, United States of America and [3]Cells-in-Motion Cluster of Excellence, Universität Münster, Waldeyerstraße 15, 48149 Münster, Germany

*Corresponding author

Email: Günter Haufe - haufe@uni.muenster.de

General procedure, synthesis of the aldol products, spectroscopic data, and copies of ^{1}H, ^{13}C, and ^{19}F NMR spectra

Table of contents

Experimental S2

General S2

General procedure S2

Spectroscopic data of *para*-substituted products S3

Spectroscopic data of *meta*-substituted products S10

Spectroscopic data of *ortho*-substituted products S12

Spectroscopic data of product of acetaldehyde S13

Spectroscopic data of SF_5-acetic acid morpholide (**8**), its ketene silylaminal, and the Aldol addition product **9** (crude NMR experiment) S14

References S16

Copies of spectra of *para*-substituted compounds S17

Copies of spectra of *meta*-substituted compounds S46

Copies of spectra of *ortho*-substituted compounds S55

Copies of spectra of product of acetaldehyde S58

Copies of spectra of compounds **8** and **9** S61

Experimental

General

All reactions were performed under argon atmosphere applying Schlenk techniques. Dichloromethane was dried and distilled over CaH_2 prior to use. TLC was performed on coated silica gel plates Merck 60 F_{254}. The spots were detected with alkaline $KMnO_4$ solution. For the purification of the compounds by column chromatography silica gel Merck 60 (0.063–0.2 mm) was used. Solvents for chromatography were purified prior to use. The NMR spectra were recorded on Bruker AV300 MHz and AV400 MHz spectrometers. ^{1}H NMR spectra were referenced to TMS as the internal standard, ^{13}C NMR spectra were referenced to the used deuterated solvents, and $CFCl_3$ was used as the internal standard for the ^{19}F NMR spectra. The multiplicity of the ^{13}C NMR signals (with regard to the C,H-coupling) was determined by DEPT technique. Technically the SF_5 group is an AB_4 spin system, although with high-field NMR spectrometers this pattern moves toward AX_4. In all cases the expected nine lines of the A spectrum are clearly resolved in the ^{19}F NMR spectra. Conveniently they are marked as quintet (qn). In the B spectrum lines are overlapping and the equatorial fluorine atoms appears as a doublet of multiplets (dm). The coupling between the axial and the equatorial fluorine atoms correlates with doublet coupling constant. ESI mass spectrometry was performed on a MicroTof (Bruker Daltonics, Bremen) spectrometer.

Synthesis of α-SF_5-substituted β-hydroxycarboxylic acid derivatives by silicon-mediated Mukaiyama aldol reaction

General procedure:

In an oven dried pressure vessel under argon atmosphere, $SF_5CH_2C(O)OC_8H_{17}$ (**1**) (76 mg, 0.25 mmol, 1.0 equiv) was dissolved in CH_2Cl_2 (2 mL) and TMSOTf (84 mg, 0.38 mmol, 1.5 equiv) and Et_3N (38 mg, 0.38 mmol, 1.5 equiv) were added successively. The mixture was refluxed for 4 hours and then cooled down to 0 °C. Under stirring the corresponding aldehyde (1.0 equiv) and $TiCl_4$ (14 mg, 0.075 mmol, 0.3 equiv) were added. The mixture was heated at 40 °C for 15 hours and subsequently quenched with ice-water (10 mL). The phases were separated, and the aqueous was extracted with CH_2Cl_2 (4 × 10 mL). The combined organic layers were dried with $MgSO_4$, and the solvent was evaporated under reduced pressure. The crude product was analyzed by ^{19}F NMR spectroscopy. The major *syn*-products **2** were separated by column chromatography (silica gel) and analyzed. The ^{1}H and ^{19}F NMR spectral data of the minor products **3** were extracted from the spectra of the crude mixtures and compared to the data of authentic *anti*-compounds synthesized by the boron mediated aldol reaction [1].

Octyl 3-hydroxy-3-(4-nitrophenyl)-2-(pentafluorosulfanyl)propanoate (2a/3a)

According to the general procedure, octyl 2-(pentafluorosulfanyl)acetate (84 mg, 0.28 mmol, 1.0 equiv) was reacted with 4-nitrobenzaldehyde (44 mg, 0.28 mmol, 1 equiv). Yield: 130 mg (crude; portion of aldol products: 53%; *syn*/*anti* ratio: 93:7 (^{19}F NMR)). The crude product was purified by column chromatography (toluene/diethyl ether, 99:1) to give a colorless clear oil. Yield: 51 mg (40%; *syn*/*anti* ratio: 99:1 (^{19}F NMR)).

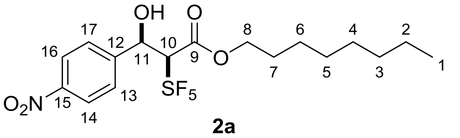

2a

^{1}H NMR (300 MHz, $CDCl_3$): δ 0.88 (t, $^3J_{H,H}$ = 6.8 Hz, 3H, 1-CH_3), 1.06 – 1.34 (m, 10H, 2-/3-/4-/5-/6-CH_2), 1.35 – 1.49 (m, 2H, 7-CH_2), 3.20 (s, 1H, OH), 3.92 (t, $^3J_{H,H}$ = 6.6 Hz, 2H, 8-CH_2), 4.75 (dqn, $^3J_{H,H}$ = 8.5 Hz, $^3J_{H,F}$ = 5.8 Hz, 1H, 10-CH), 5.57 (d, $^3J_{H,H}$ = 8.5 Hz, 1H, 11-CH), 7.61 (d, $^3J_{H,H}$ = 8.7 Hz, 2H, 13-/17-CH), 8.22 (d, $^3J_{H,H}$ = 8.8 Hz, 2H, 14-/16-CH). **^{13}C NMR** (100 MHz, $CDCl_3$): δ 14.2 (q, C-1), 22.7 (t, C-2), 25.6 (t, C-6), 28.1 (t, C-7), 29.1, 29.2 (t, C-4/-5), 31.8 (t, C-3), 67.7 (t, C-8), 71.9 (dqn, $^3J_{C,F}$ = 2.4 Hz, C-11), 85.7 (dqn, $^2J_{C,F}$ = 9.5 Hz, C-10), 124.1 (d, C-14/-16), 127.4 (d, C-13/-17), 146.4 (s, C-12), 148.1 (s, C-15), 165.4 (qn, $^3J_{C,F}$ = 2.8 Hz, C-9). **^{19}F NMR** (282 MHz, $CDCl_3$): δ 66.5 (dm, $^2J_{F,F}$ = 147.3 Hz, 4F, 10-SF_5), 79.7 (qn, $^2J_{F,F}$ = 147.1 Hz, 1F, 10-SF_5). **MS-ES(+)-EM**: *m*/*z* calcd. for $C_{17}H_{24}F_5NO_5SNa^+$ 472.1188, found 472.1188 $[M+Na]^+$.

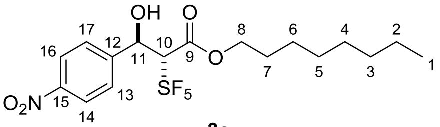

3a

^{1}H NMR (300 MHz, $CDCl_3$): δ 0.88 (t, $^3J_{H,H}$ = 6.8 Hz, 3H, 1-CH_3), 1.06 – 1.34 (m, 10H, 2-/3-/4-/5-/6-CH_2), 1.49 – 1.58 (m, 2H, 7-CH_2), 1.63 (s, 1H, OH), 4.03 – 4.23 (m, 2H, 8-CH_2), 4.63 (dqn, $^3J_{H,F}$ = 5.9 Hz, $^3J_{H,H}$ = 3.4 Hz, 1H, 10-CH), 5.63 (d, $^3J_{H,H}$ = 3.3 Hz, 1H, 11-CH), 7.55 (d, $^3J_{H,H}$ = 8.7 Hz, 2H, 13-/17-CH), 8.25 (d, $^3J_{H,H}$ = 9.0 Hz, 2H, 14-/16-CH). **^{19}F NMR** (282 MHz, $CDCl_3$): δ 68.0 (dm, $^2J_{F,F}$ = 147.2 Hz, 4F, 10-SF_5), 80.2 (qn, $^2J_{F,F}$ = 147.3 Hz, 1F, 10-SF_5).

Octyl 3-hydroxy-2-(pentafluorosulfanyl)-3-phenylpropanoate (2b/3b)

According to the general procedure, octyl 2-(pentafluorosulfanyl)acetate (86 mg, 0.29 mmol, 1.0 equiv) was reacted with benzaldehyde (31 mg, 0.29 mmol, 1.0 equiv). Yield: 120 mg (crude, portion of aldol products: 44%; *syn*/*anti*-ratio 86:14 (^{19}F NMR)). The crude product was purified by column chromatography (silica gel, toluene/diethyl ether, 99:1) to give a colorless oil. Yield: 35 mg (30%; *syn*/*anti*-ratio 93:7 (^{19}F NMR)).

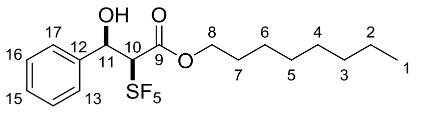

2b

^{1}H NMR (300 MHz, $CDCl_3$): δ 0.89 (t, $^{3}J_{H,H}$ = 6.8 Hz, 3H, 1-CH_3), 1.03 – 1.39 (m, 12H, 2-/3-/4-/5-/6-/7-CH_2), 2.67 (s, 1H, OH), 3.69 – 3.91 (m, 2H, 8-CH_2), 4.78 (dqn, $^{3}J_{H,H}$ = 9.4 Hz, $^{3}J_{H,F}$ = 5.9 Hz, 1H, 10-CH), 5.43 (d, $^{3}J_{H,H}$ = 9.5 Hz, 1H, 11-CH), 7.28 – 7.44 (m, 5H, 13-/14-/15-/16-/17-CH). **^{13}C NMR** (100 MHz, $CDCl_3$): δ 14.2 (q, C-1), 22.8 (t, C-2), 25.7 (t, C-6), 28.0 (t, C-7), 29.1, 29.2 (t, C-4/-5), 31.9 (t, C-3), 66.6 (t, C-8), 73.7 (dqn, $^{3}J_{C,F}$ = 2.4 Hz, C-11), 90.2 (dqn, $^{2}J_{C,F}$ = 8.4 Hz, C-10), 127.8 (d, C-13/-17), 128.9 (d, C-14/-16), 129.4 (d, C-15), 138.4 (s, C-12), 163.7 (qn, $^{3}J_{C,F}$ = 3.1 Hz, C-9). **^{19}F NMR** (282 MHz, $CDCl_3$): δ 66.8 (dm, $^{2}J_{F,F}$ = 146.5 Hz, 4F, 10-SF_5), 81.6 (qn, $^{2}J_{F,F}$ = 147.3 Hz, 1F, 10-SF_5). **MS-ES(+)-EM**: *m*/*z* calcd. for $C_{17}H_{25}F_5O_3SNa^+$ 427.1337; found: 427.1338 $[M+Na]^+$.

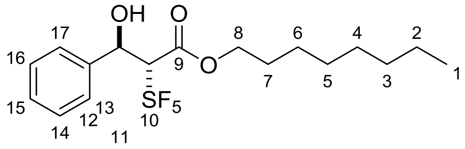

3b

^{1}H NMR (300 MHz, $CDCl_3$): δ 0.89 (t, $^{3}J_{H,H}$ = 6.8 Hz, 3H, 1-CH_3), 1.03 – 1.39 (m, 10H, 2-/3-/4-/5-/6-CH_2), 1.45 – 1.54 (m, 2H, 7-CH_2), 1.60 (s, 1H, OH), 4.06 – 4.13 (m, 2H, 8-CH_2), 4.66 (dqn, $^{3}J_{H,F}$ = 6.4 Hz, $^{3}J_{H,H}$ = 3.6 Hz, 1H, 10-CH), 5.50 (d, $^{3}J_{H,H}$ = 3.5 Hz, 1H, 11-CH), 7.28 – 7.44 (m, 5H, 13-/14-/15-/16-/17-CH). **^{19}F NMR** (282 MHz, $CDCl_3$): δ 66.0 (dm, $^{2}J_{F,F}$ = 147.2 Hz, 4F, 10-SF_5), 80.4 (qn, $^{2}J_{F,F}$ = 147.1 Hz, 1F, 10-SF_5). Spectra agree to those of an authentic sample.[1]

Octyl 3-(4-fluorophenyl)-3-hydroxy-2-(pentafluorosulfanyl)propanoate (2c/3c)

According to the general procedure, octyl 2-(pentafluorosulfanyl)acetate (80 mg, 0.27 mmol, 1.0 equiv) was reacted with 4-fluorobenzaldehyde (34 mg, 0.27 mmol, 1.0 equiv). Yield: 115 mg (crude, portion of aldol products: 44%; *syn*/*anti*-ratio 86:14 (^{19}F NMR)). The crude product was purified by column chromatography (silica gel, toluene/diethyl ether, 99:1) to give a colorless oil. Yield: 42 mg (37%; *syn*/*anti*-ratio 91:9 (^{19}F NMR)).

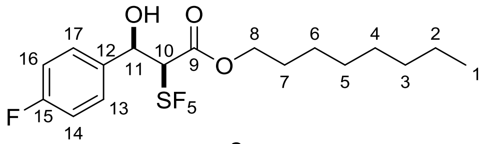

2c

^{1}H NMR (300 MHz, $CDCl_3$): δ 0.89 (t, $^{3}J_{H,H}$ = 6.8 Hz, 3H, 1-CH_3), 1.06 – 1.45 (m, 12H, 2-/3-/4-/5-/6-/7-CH_2), 2.73 (s, 1H, OH), 3.75 – 3.94 (m, 2H, 8-CH_2), 4.71 (dqn, $^{3}J_{H,H}$ = 9.4 Hz, $^{3}J_{H,F}$ = 5.8 Hz, 1H, 10-CH), 5.44 (d, $^{3}J_{H,H}$ = 9.4 Hz, 1H, 11-CH), 6.96 – 7.12 (m, 2H, 14-/16-

CH), 7.27 – 7.46 (m, 2H, 13-/17-CH). **^{13}C NMR** (100 MHz, $CDCl_3$): *δ* 14.2 (q, C-1), 22.8 (t, C-2), 25.7 (t, C-6), 28.1 (t, C-7), 29.1, 29.2 (t, C-4/-5), 31.9 (t, C-3), 66.7 (t, C-8), 73.0 (dqn, $^3J_{C,F}$ = 2.2 Hz, C-11), 90.1 (dqn, $^2J_{C,F}$ = 8.5 Hz, C-10), 115.8 (dd, $^2J_{C,F}$ = 21.7 Hz, C-14/-16), 129.7 (dd, $^3J_{C,F}$ = 8.4 Hz, C-13/-17), 134.2 (qn, $^4J_{C,F}$ = 1.4 Hz, C-12), 163.1 (d, $^1J_{C,F}$ = 248.8 Hz, C-15), 163.7 (qn, $^3J_{C,F}$ = 2.8 Hz, C-9). **^{19}F NMR** (282 MHz, $CDCl_3$): *δ* -112.3 (tt, $^3J_{H,F}$ = 8.5 Hz, $^4J_{H,F}$ = 5.3 Hz, 1F, 15-CF), 66.9 (dm, $^2J_{F,F}$ = 146.6 Hz, 4F, 10-SF_5), 81.5 (qn, $^2J_{F,F}$ = 147.0 Hz, 1F, 10-SF_5). **MS-ES(+)-EM**: *m/z* calcd. for $C_{17}H_{24}F_6O_3SNa^+$ 445.1243, found 445.1237 $[M+Na]^+$.

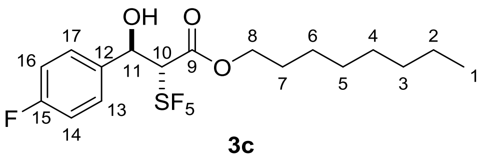

3c

^{1}H NMR (300 MHz, $CDCl_3$): *δ* 0.89 (t, $^3J_{H,H}$ = 6.8 Hz, 3H, 1-CH_3), 1.06 – 1.45 (m, 10H, 2-/3-/4-/5-/6-CH_2), 1.45 – 1.58 (m, 2H, 7-CH_2), 4.06 – 4.18 (m, 2H, 8-CH_2), 4.61 (dqn, $^3J_{H,F}$ = 6.4 Hz, $^3J_{H,H}$ = 3.8 Hz, 1H, 10-CH), 5.49 (d, $^3J_{H,H}$ = 3.8 Hz 1H, 11-CH), 6.96 – 7.12 (m, 2H, 14-/16-CH), 7.27 – 7.46 (m, 2H, 13-/17-CH). **^{19}F NMR** (282 MHz, $CDCl_3$): *δ* -113.6 (tt, $^3J_{H,F}$ = 8.5 Hz, $^4J_{H,F}$ = 5.1 Hz, 1F, 15-CF), 66.2 (dm, $^2J_{F,F}$ = 147.3 Hz, 4F, 10-SF_5), 80.3 (qn, $^2J_{F,F}$ = 147.4 Hz, 1F, 10-SF_5). Spectra agree to those of an authentic sample.[1]

Octyl 3-(4-chlorophenyl)-3-hydroxy-2-(pentafluorosulfanyl)propanoates (2d/3d)

According to the general procedure, octyl 2-(pentafluorosulfanyl)acetate (80 mg, 0.27 mmol, 1.0 equiv) was reacted with 4-chlorobenzaldehyde (38 mg, 0.27 mmol, 1 equiv). Yield: 120 mg (crude, portion of aldol products: 38%; *syn*/*anti*-ratio 81:19 (^{19}F NMR)). The crude product was purified by column chromatography (silica gel, toluene/diethyl ether, 99:1) to give a colorless oil. Yield: 22 mg (19%; *syn*/*anti*-ratio 87:13 (^{19}F NMR)).

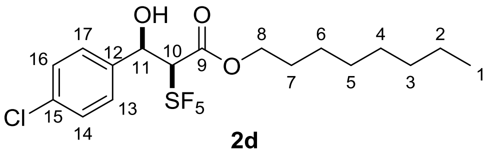

2d

^{1}H NMR (300 MHz, $CDCl_3$): *δ* 0.89 (t, $^3J_{H,H}$ = 6.9 Hz, 3H, 1-CH_3), 1.05 – 1.41 (m, 12H, 2-/3-/4-/5-/6-/7-CH_2), 2.74 (s, 1H, OH), 3.77 – 3.94 (m, 2H, 8-CH_2), 4.71 (dqn, $^3J_{H,H}$ = 9.3 Hz, $^3J_{H,F}$ = 5.9 Hz, 1H, 10-CH), 5.43 (d, $^3J_{H,H}$ = 9.3 Hz, 1H, 11-CH), 7.27 – 7.39 (m, 4H, 13-/14-/16-/17-CH). **^{13}C NMR** (100 MHz, $CDCl_3$): *δ* 14.2 (q, C-1), 22.8 (t, C-2), 25.7 (t, C-6), 28.1 (t, C-7), 29.1, 29.2 (t, C-4/-5), 31.9 (t, C-3), 66.8 (t, C-8), 73.1 (dqn, $^3J_{C,F}$ = 2.2 Hz, C-11), 89.9 (dqn, $^2J_{C,F}$ = 8.9 Hz, C-10), 129.1 (d, C-14/-16), 129.2 (d, C-13/-17), 135.3 (s, C-15), 136.8 (qn, $^4J_{C,F}$ = 1.4 Hz, C-12), 163.7 (qn, $^3J_{C,F}$ = 3.2 Hz, C-9). **^{19}F NMR** (282 MHz, $CDCl_3$): *δ* 67.0

(dm, $^2J_{F,F}$ = 146.5 Hz, 4F, 10-SF_5), 81.4 (qn, $^2J_{F,F}$ = 146.8 Hz, 1F, 10-SF_5). **MS-ES(+)-EM**: *m*/*z* calcd. for $C_{17}H_{24}ClF_5O_3SNa^+$ 461.0947; found: 461.0942 $[M+Na]^+$.

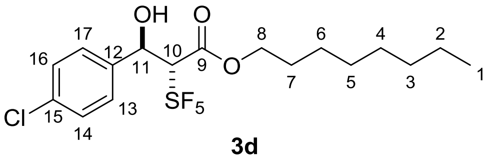

3d

^{1}H NMR (300 MHz, $CDCl_3$): δ 0.89 (t, $^3J_{H,H}$ = 6.9 Hz, 3H, 1-CH_3), 1.05 – 1.41 (m, 10H, 2-/3-/4-/5-/6-CH_2), 1.46 – 1.57 (m, 2H, 7-CH_2), 4.06 – 4.18 (m, 2H, 8-CH_2), 4.60 (dqn, $^3J_{H,F}$ = 6.4 Hz, $^3J_{H,H}$ = 3.7 Hz, 1H, 10-CH), 5.49 (s, $^3J_{H,H}$ = 3.7 Hz, 1H, 11-CH), 7.27 – 7.39 (m, 4H, 13-/14-/16-/17-CH). **^{19}F NMR** (282 MHz, $CDCl_3$): δ 66.2 (dm, $^2J_{F,F}$ = 147.1 Hz, 4F, 10-SF_5), 80.2 (qn, $^2J_{F,F}$ = 147.1 Hz, 1F, 10-SF_5).

Octyl 3-(4-bromophenyl)-3-hydroxy-2-(pentafluorosulfanyl)propanoate (2e/3e)

According to the general procedure, octyl 2-(pentafluorosulfanyl)acetate (80 mg, 0.27 mmol, 1.0 equiv) was reacted with 4-bromobenzaldehyde (50 mg, 0.27 mmol, 1.0 equiv). Yield: 142 mg (crude, portion of aldol products: 39%; *syn*/*anti*-ratio 83:17 (^{19}F NMR)). The crude product was purified by column chromatography (silica gel, pentane/toluene, 50:50) to give a colorless oil. Yield: 26 mg (19%; *syn*/*anti*-ratio 86:14 (^{19}F NMR)).

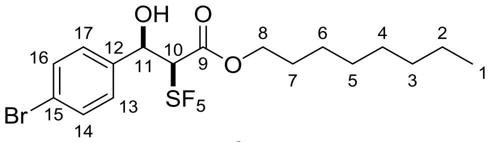

2e

^{1}H NMR (300 MHz, $CDCl_3$): δ 0.89 (t, $^3J_{H,H}$ = 6.8 Hz, 3H, 1-CH_3), 1.08 – 1.44 (m, 12H, 2-/3-/4-/5-/6-/7-CH_2), 2.74 (s, 1H, OH), 3.77 – 3.95 (m, 2H, 8-CH_2), 4.70 (dqn, $^3J_{H,H}$ = 9.3 Hz, $^3J_{H,F}$ = 5.8 Hz, 1H, 10-CH), 5.42 (d, $^3J_{H,H}$ = 9.3 Hz, 1H, 11-CH), 7.18 – 7.31 (m, 2H, 13-/17-CH), 7.44 – 7.55 (m, 2H, 14-/16-CH). **^{13}C NMR** (100 MHz, $CDCl_3$): δ 14.2 (q, C-1), 22.8 (t, C-2), 25.7 (t, C-6), 28.1 (t, C-7), 29.2, 29.2 (t, C-4/-5), 31.9 (t, C-3), 66.8 (t, C-8), 73.1 (dqn, $^3J_{C,F}$ = 2.4 Hz, C-11), 89.8 (dqn, $^2J_{C,F}$ = 8.7 Hz, C-10), 123.5 (s, C-15), 129.5 (d, C-13/-17), 132.0 (d, C-14/-16), 137.3 (s, C-12), 163.7 (qn, $^3J_{C,F}$ = 3.2 Hz, C-9). **^{19}F NMR** (282 MHz, $CDCl_3$): δ 67.0 (dm, $^2J_{F,F}$ = 146.8 Hz, 4F, 10-SF_5), 81.4 (qn, $^2J_{F,F}$ = 148.5 Hz, 1F, 10-SF_5). **MS-ES(+)-EM**: *m*/*z* calcd. for $C_{17}H_{24}[^{79}Br]F_5O_3SNa^+$ 505.0442; found: 505.0456 $[M+Na]^+$.

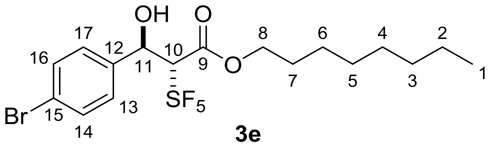

3e

^{1}**H NMR** (300 MHz, $CDCl_3$): δ 0.89 (t, $^3J_{H,H}$ = 6.8 Hz, 3H, 1-CH_3), 1.08 – 1.44 (m, 10H, 2-/3-/4-/5-/6-CH_2), 1.47 – 1.56 (m, 2H, 7-CH_2), 1.58 (s, 1H, OH), 4.08 – 4.16 (m, 2H, 8-CH_2), 4.60 (dqn, $^3J_{H,F}$ = 6.4 Hz, $^3J_{H,H}$ = 3.6 Hz, 1H, 10-CH), 5.49 (d, $^3J_{H,H}$ = 3.6 Hz, 1H, 11-CH), 7.18 – 7.31 (m, 2H, 13-/17-CH), 7.44 – 7.55 (m, 2H, 14-/16-CH). ^{19}**F NMR** (282 MHz, $CDCl_3$): δ 66.2 (dm, $^2J_{F,F}$ = 149.8 Hz, 4F, 10-SF_5), 80.2 (qn, $^2J_{F,F}$ = 149.9 Hz, 1F, 10-SF_5). Spectra agree to those of an authentic sample.[1]

Octyl 3-hydroxy-2-(pentafluorosulfanyl)-3-[4-(pentafluorosulfanyl)phenyl]propanoate (2f/3f)

According to the general procedure, octyl 2-(pentafluorosulfanyl)acetate (80 mg, 0.27 mmol, 1.0 equiv) was reacted with 4-(pentafluorosulfanyl)benzaldehyde (63 mg, 0.27 mmol, 1 equiv). Yield: 155 mg (crude, portion of aldol products: 42%; *syn*/*anti*-ratio 95:5 (^{19}F NMR)). The crude product was purified by column chromatography (silica gel, pentane/toluene, 50:50) to give a colorless oil. Yield: 34 mg (22%; *syn*/*anti*-ratio 95:5 (^{19}F NMR)).

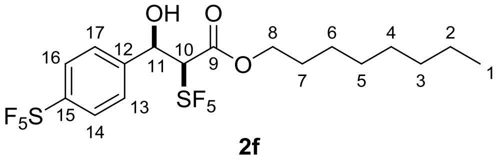

2f

^{1}**H NMR** (300 MHz, $CDCl_3$): δ 0.88 (t, $^3J_{H,H}$ = 7.0 Hz, 3H, 1-CH_3), 1.07 – 1.45 (m, 12H, 2-/3-/4-/5-/6-/7-CH_2), 2.98 (s, 1H, OH), 3.82 – 3.93 (m, 2H, 8-CH_2), 4.73 (dqn, $^3J_{H,H}$ = 8.8 Hz, $^3J_{H,F}$ = 5.7 Hz, 1H, 10-CH), 5.51 (d, $^3J_{H,H}$ = 8.8 Hz, 1H, 11-CH), 7.52 (d, $^3J_{H,H}$ = 8.4 Hz, 2H, 13-/17-CH). 7.70 – 7.81 (m, 2H, 14-/16-CH). ^{13}**C NMR** (100 MHz, $CDCl_3$): δ 14.2 (q, C-1), 22.7 (t, C-2), 25.6 (t, C-6), 28.0 (t, C-7), 29.0, 29.2 (t, C-4/-5), 31.8 (t, C-3), 67.1 (t, C-8), 72.8 (dqn, $^3J_{C,F}$ = 1.9 Hz, C-11), 89.4 (dqn, $^2J_{C,F}$ = 8.9 Hz, C-10), 126.5 (dqn, $^3J_{C,F}$ = 4.3 Hz, C-14/-16), 128.2 (d, C-13/-17), 142.0 (s, C-12), 154.2 (qn, $^2J_{C,F}$ = 16.6 Hz, C-15), 163.9 (qn, $^3J_{C,F}$ = 3.2 Hz, C-9). ^{19}**F NMR** (282 MHz, $CDCl_3$): δ 62.2 (dm, $^2J_{F,F}$ = 150.4 Hz, 4F, 15-SF_5), 67.6 (dm, $^2J_{F,F}$ = 147.1 Hz, 4F, 10-SF_5), 81.0 (qn, $^2J_{F,F}$ = 146.8 Hz, 1F, 10-SF_5), 83.1 (qn, $^2J_{F,F}$ = 149.9 Hz, 1F, 15-SF_5). **MS-ES(+)-EM**: *m*/*z* calcd. for $C_{17}H_{24}F_{10}O_3S_2Na^+$ 553.0899; found: 553.0901 $[M+Na]^+$.

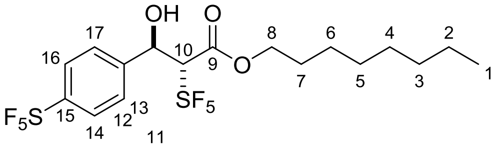

3f

^{1}**H NMR** (300 MHz, $CDCl_3$): δ 0.88 (t, $^3J_{H,H}$ = 7.0 Hz, 3H, 1-CH_3), 1.07 – 1.45 (m, 10H, 2-/3-/4-/5-/6-CH_2), 1.46 – 1.58 (qn, 2H, 7-CH_2), 1.59 (s, 1H, OH), 4.02 – 4.27 (m, 2H, 8-CH_2), 4.63 (dqn, $^3J_{H,F}$ = 6.2 Hz, $^3J_{H,H}$ = 3.7 Hz, 1H, 10-CH), 5.57 (s, 1H, $^3J_{H,H}$ = 3.7 Hz, 11-CH), 7.45 (d,

$^3J_{H,H}$ = 8.4 Hz, 2H, 13-/17-CH). 7.70 – 7.81 (m, 2H, 14-/16-CH). **^{19}F NMR** (282 MHz, $CDCl_3$): δ 62.3 (dm, $^2J_{F,F}$ = 150.4 Hz, 4F, 15-SF_5), 66.3 (dm, $^2J_{F,F}$ = 147.4 Hz, 4F, 10-SF_5), 79.8 (qn, $^2J_{F,F}$ = 147.7 Hz, 1F, 10-SF_5), 83.6 (qn, $^2J_{F,F}$ = 150.3 Hz, 1F, 15-SF_5). Spectra agree with those of an authentic sample.[1]

Octyl (*E*)-3-(4-methylphenyl)-2-(pentafluorosulfanyl)acrylate (4g)

According to the general procedure, octyl 2-(pentafluorosulfanyl)acetate (75 mg, 0.25 mmol, 1.0 equiv) was reacted with 4-methylbenzaldehyde (30 mg, 0.25 mmol, 1.0 equiv). Instead of the expected aldol addition product, the corresponding condensation product was found exclusively. The product was purified by column chromatography (silica gel, pentane/diethyl ether, 30:1) and a second chromatography (pentane/toluene, 5:1) to give a colorless oil. Yield: 31 mg (32%).

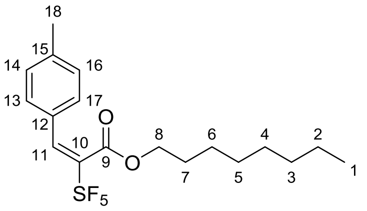

4g

^{1}H NMR (400 MHz, $CDCl_3$): δ 0.88 (t, $^3J_{H,H}$ = 7.0 Hz, 3H, 1-CH_3), 1.10 – 1.33 (m, 10H, 2-/3-/4-/5-/6-CH_2), 1.50 – 1.60 (m, 2H, 7-CH_2), 2.37 (s, 3H, 18-CH_3), 4.18 (t, $^3J_{H,H}$ = 6.6 Hz, 2H, 8-CH_2), 7.17 – 7.25 (m, 4H, 13-/14-/16-/17-CH), 7.45 (s, 1H, 11H). **^{13}C NMR** (100 MHz, $CDCl_3$): δ 14.2 (q, C-1), 21.6 (q, C-18), 22.8 (t, C-2), 25.8 (t, C-6), 28.1 (t, C-7), 29.2, 29.2 (t, C-4/-5), 31.9 (t, C-3), 67.2 (t, C-8), 128.3 (s, C-12), 128.9 (d, C-13/-17), 129.8 (d, C-14/-16), 136.9 (dqn, $^3J_{C,F}$ = 5.9 Hz, C-11), 146.0 (qn, $^2J_{C,F}$ = 17.5 Hz, C-10), 141.2 (s, C-15), 163.0 (qn, $^3J_{C,F}$ = 2.1 Hz, C-9). **^{19}F NMR** (282 MHz, $CDCl_3$): δ 64.5 (dm, $^2J_{F,F}$ = 150.9 Hz, 4F, 10-SF_5), 81.7 (qn, $^2J_{F,F}$ = 151.0 Hz, 1F, 10-SF_5). **MS-ES(+)-EM**: *m*/*z* calcd. for $C_{18}H_{25}F_5O_3SNa^+$ 423.1388; found: 423.1386 $[M+Na]^+$.

Octyl (*E*)-3-(4-methoxyphenyl)-2-(pentafluorosulfanyl)acrylate (4h)

According to the general procedure, octyl 2-(pentafluorosulfanyl)acetate (80 mg, 0.27 mmol, 1.0 equiv) was reacted with 4-methoxybenzaldehyde (37 mg, 0.27 mmol, 1.0 equiv). Instead of the expected aldol addition product, the corresponding condensation product was found exclusively. The product was purified by column chromatography (silica gel, pentane/diethyl ether, 30:1) to give a colorless oil. Yield: 40 mg (36%).

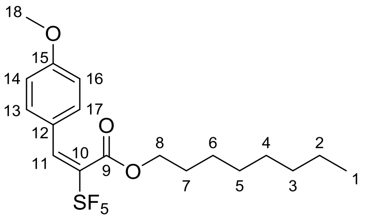

4h

^{1}H NMR (300 MHz, $CDCl_3$): δ 0.87 (t, $^{3}J_{H,H}$ = 6.8 Hz, 3H, 1-CH_3), 1.14 – 1.34 (m, 10H, 2-/3-/4-/5-/6-CH_2), 1.51 – 1.66 (m, 2H, 7-CH_2), 3.83 (s, 3H, 18-CH_3), 4.21 (t, $^{3}J_{H,H}$ = 6.6 Hz, 2H, 8-CH_2), 6.84 – 6.94 (m, 2H, 14-/16-CH), 7.25 – 7.33 (m, 2H, 13-/17-CH), 7.41 (s, 1H, 11-CH). **^{13}C NMR** (100 MHz, $CDCl_3$): δ 14.2 (q, C-1), 22.8 (t, C-2), 25.8 (t, C-6), 28.2 (t, C-7), 29.2, 29.2 (t, C-4/-5), 31.9 (t, C-3), 55.5 (q, C-18), 67.1 (t, C-8), 114.5 (d, C-14/-16), 123.4 (s, C-12), 130.8 (d, C-13/-17), 136.5 (dqn, $^{3}J_{C,F}$ = 6.0 Hz, C-11), 144.8 (qn, $^{2}J_{C,F}$ = 17.6 Hz, C-10), 161.7 (s, C-15), 163.3 (qn, $^{3}J_{C,F}$ = 3.0 Hz, C-9). **^{19}F NMR** (282 MHz, $CDCl_3$): δ 64.8 (dm, $^{2}J_{F,F}$ = 151.0 Hz, 4F, 10-SF_5), 82.2 (qn, $^{2}J_{F,F}$ = 151.6 Hz, 1F, 10-SF_5). **MS-ES(+)-EM**: *m*/*z* calcd. for $C_{18}H_{25}F_5O_3SNa^+$ 439.1337; found: 439.1337 $[M+Na]^+$.

Octyl (*E*)-3-(4-ethoxyphenyl)-2-(pentafluorsulfanyl)acrylate (4i)

According to the general procedure, octyl 2-(pentafluorosulfanyl)acetate (80 mg, 0.27 mmol, 1.0 equiv) was reacted with 4-ethoxybenzaldehyde (41 mg, 0.27 mmol, 1.0 equiv). Instead of the expected aldol addition product, the corresponding condensation product was found exclusively. The product was purified by column chromatography (silica gel, pentane/diethyl ether, 30:1) and a second chromatography (pentane/toluene, 5:1) to give a colorless oil. Yield: 42 mg (36%).

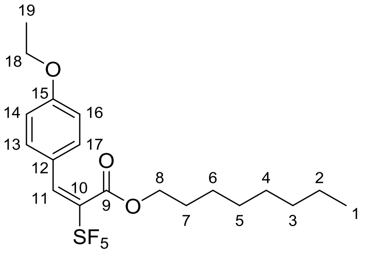

4i

^{1}H NMR (300 MHz, $CDCl_3$): δ 0.87 (t, $^{3}J_{H,H}$ = 6.8 Hz, 3H, 1-CH_3), 1.12 – 1.35 (m, 10H, 2-/3-/4-/5-/6-CH_2), 1.46 (t, $^{3}J_{H,H}$ = 7.0 Hz, 19-CH_3), 1.52 – 1.66 (m, 2H, 7-CH_2), 4.05 (q, $^{3}J_{H,H}$ = 7.0 Hz, 18-CH_2), 4.20 (t, $^{3}J_{H,H}$ = 6.6 Hz, 2H, 8-CH_2), 6.87 (d, $^{3}J_{H,H}$ = 8.8 Hz, 2H, 14-/16-CH), 7.28 (d, $^{3}J_{H,H}$ = 8.7 Hz, 2H, 13-/17-CH), 7.41 (s, 1H, 11-CH). **^{13}C NMR** (100 MHz, $CDCl_3$): δ 14.2 (q, C-1), 14.8 (q, C-19), 22.8 (t, C-2), 25.8 (t, C-6), 28.2 (t, C-7), 29.21, 29.24 (t, C-4/-5), 31.9 (t, C-3), 63.8 (t, C-18), 67.1 (t, C-8), 115.0 (d, C-14/-16), 123.1 (s, C-12), 130.9 (d, C-13/-17), 136.6 (dqn, $^{3}J_{C,F}$ = 5.8 Hz, C-11), 144.6 (qn, $^{2}J_{C,F}$ = 17.0 Hz, C-10), 161.1 (s, C-15), 163.3 (qn, $^{3}J_{C,F}$ = 1.9 Hz, C-9). **^{19}F NMR** (282 MHz, $CDCl_3$): δ 64.8 (dm,

$^2J_{F,F}$ = 151.0 Hz, 4F, 10-SF_5), 82.3 (qn, $^2J_{F,F}$ = 150.3 Hz, 1F, 10-SF_5). **MS-ES(+)-EM**: *m*/*z* calcd. for $C_{19}H_{27}F_5O_3SNa^+$ 453.1493; found: 453.1482 $[M+Na]^+$.

Octyl 3-hydroxy-3-(3-nitrophenyl)-2-(pentafluorosulfanyl)propanoate (2j/3j)

According to the general procedure, octyl 2-(pentafluorosulfanyl)acetate (80 mg, 0.27 mmol, 1.0 equiv) was reacted with 3-nitrobenzaldehyde (41 mg, 0.27 mmol, 1.0 equiv). Yield: 129 mg (crude, portion of aldol products: 61%; *syn*/*anti*-ratio 90:10 (^{19}F NMR)). The crude product was purified by column chromatography (silica gel, toluene/diethyl ether, 99:1) and second chromatography (cyclohexane/ethyl acetate, 2:1) to give a colorless oil. Yield: 52 mg (44%; *syn*/*anti*-ratio 90:10 (^{19}F NMR)).

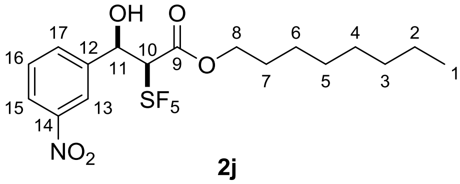

2j

^{1}H NMR (300 MHz, $CDCl_3$): *δ* 0.87 (t, $^3J_{H,H}$ = 6.7 Hz, 3H, 1-CH_3), 1.04 – 1.48 (m, 12H, 2-/3-/4-/5-/6-/7-CH_2), 2.96 (s, 1H, OH), 3.91 (t, $^3J_{H,H}$ = 6.7 Hz, 2H, 8-CH_2), 4.76 (dqn, $^3J_{H,H}$ = 8.7 Hz, $^3J_{H,F}$ = 5.8 Hz, 1H, 10-CH), 5.58 (d, $^3J_{H,H}$ = 8.7 Hz, 1H, 11-CH), 7.56 (m, 1H, 16-CH), 7.77 (m, 1H, 17-CH), 8.21 (m, 1H, 15-CH), 8.31 (t, $^4J_{H,H}$ = $^4J_{H,H}$ = 2.0 Hz, 1H, 13-CH). **^{13}C NMR** (100 MHz, $CDCl_3$): *δ* 14.2 (q, C-1), 22.7 (t, C-2), 25.6 (t, C-6), 28.0 (t, C-7), 29.1, 29.2 (t, C-4/-5), 31.8 (t, C-3), 67.2 (t, C-8), 72.7 (d, C-11), 89.3 (dqn, $^2J_{C,F}$ = 9.1 Hz, C-10), 122.8 (d, C-13), 124.1 (d, C-15), 129.8 (d, C-16), 133.9 (d, C-17), 140.5 (s, C-12), 148.3 (s, C-14), 163.9 (qn, $^3J_{C,F}$ = 3.0 Hz, C-9). **^{19}F NMR** (282 MHz, $CDCl_3$): *δ* 67.9 (dm, $^2J_{F,F}$ = 146.8 Hz, 4F, 10-SF_5), 80.9 (qn, $^2J_{F,F}$ = 147.3 Hz, 1F, 10-SF_5). **MS-ES(+)-EM**: *m*/*z* calcd. for $C_{17}H_{24}F_5NO_5SNa^+$ 472.1188; found: 472.1184 $[M+Na]^+$.

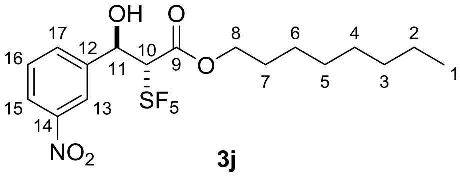

3j

^{1}H NMR (300 MHz, $CDCl_3$): *δ* 0.88 (t, $^3J_{H,H}$ = 6.7 Hz, 3H, 1-CH_3), 1.04 – 1.48 (m, 10H, 2-/3-/4-/5-/6-CH_2), 1.48 – 1.60 (m, 2H, 7-CH_2), 4.16 (m, 2H, 8-CH_2), 4.66 (dqn, $^3J_{H,F}$ = 6.2 Hz, $^3J_{H,H}$ = 4.2 Hz, 1H, 10-CH), 5.62 (d, $^3J_{H,H}$ = 4.2 Hz, 1H, 11-CH), 7.50- 7.64 (m, 1H, 16-CH), 7.69 – 7.82 (m, 1H, 17-CH), 8.16 – 8.27 (m, 1H, 15-CH), 8.31 (t, $^4J_{H,H}$ = $^4J_{H,H}$ = 2.0 Hz, 1H, 13-CH). **^{19}F NMR** (282 MHz, $CDCl_3$): *δ* 66.6 (dm, $^2J_{F,F}$ = 147.3 Hz, 4F, 10-SF_5), 79.8 (qn, $^2J_{F,F}$ = 147.3 Hz, 1F, 10-SF_5).

Octyl 3-hydroxy-3-(3-methylyphenyl)-2-(pentafluorosulfanyl)propanoate (2k/3k)

According to the general procedure, octyl 2-(pentafluorosulfanyl)acetate (80 mg, 0.27 mmol, 1.0 equiv) was reacted with 3-methylbenzaldehyde (32 mg, 0.27 mmol, 1.0 equiv). Yield: 125 mg (crude, portion of aldol products: 57%; *syn*/*anti*-ratio 73:27 (^{19}F NMR)). The crude product was purified by column chromatography (silica gel, toluene/diethyl ether, 99:1) to give a colorless oil. Yield: 45 mg (41%, *syn*/*anti*-ratio 84:16 (^{19}F NMR)).

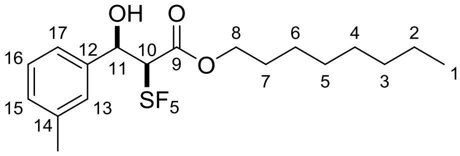

2k

^{1}H NMR (300 MHz, $CDCl_3$): δ 0.89 (t, $^3J_{H,H}$ = 6.9 Hz, 3H, 1-CH_3), 1.04 – 1.39 (m, 12H, 2-/3-/4-/5-/6-/7-CH_2), 2.36 (s, 3H, 18-CH_3), 2.62 (s, 1H, OH), 3.70 – 3.91 (m, 2H, 8-CH_2), 4.77 (dqn, $^3J_{H,H}$ = 9.5 Hz, $^3J_{H,F}$ = 5.9 Hz, 1H, 10-CH), 5.39 (d, $^3J_{H,H}$ = 9.6 Hz, 1H, 11-CH), 7.07 – 7.30 (m, 4H, 13-/15-/16/-17-CH). **^{13}C NMR** (100 MHz, $CDCl_3$): δ 14.2 (q, C-1), 21.5 (q, C-18), 22.8 (t, C-2), 25.7 (t, C-6), 28.0 (t, C-7), 29.2, 29.2 (t, C-4/-5), 31.9 (t, C-3), 66.6 (t, C-8), 73.7 (dqn, $^3J_{C,F}$ = 2.4 Hz, C-11), 90.2 (dqn, $^2J_{C,F}$ = 8.2 Hz, C-10), 124.9 (d, C-17), 128.4 (d, C-13), 128.8 (d, C-16), 130.2 (d, C-15), 138.3 (s, C-12), 138.6 (s, C-14), 163.7 (qn, $^3J_{C,F}$ = 3.2 Hz, C-9). **^{19}F NMR** (282 MHz, $CDCl_3$): δ 66.7 (dm, $^2J_{F,F}$ = 146.5 Hz, 4F, 10-SF_5), 81.7 (qn, $^2J_{F,F}$ = 145.8 Hz, 1F, 10-SF_5). **MS-ES(+)-EM**: *m*/*z* calcd. for $C_{18}H_{27}F_5O_3SNa^+$ 441.1493; found: 441.1485 $[M+Na]^+$.

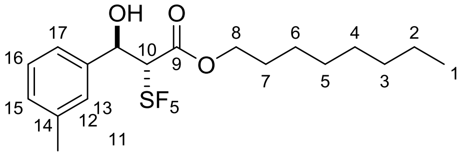

3k

^{1}H NMR (300 MHz, $CDCl_3$): δ 0.89 (t, $^3J_{H,H}$ = 6.9 Hz, 3H, 1-CH_3), 1.04 – 1.39 (m, 10H, 2-/3-/4-/5-/6-CH_2), 1.39 – 1.57 (m, 2H, 7-CH_2), 2.34 (s, 3H, 18-CH_3), 4.10 (t, $^3J_{H,H}$ = 6.6 Hz, 2H, 8-CH_2), 4.65 (dqn, $^3J_{H,F}$ = 6.5 Hz, $^3J_{H,H}$ = 3.5 Hz, 1H, 10-CH), 5.47 (s, $^3J_{H,H}$ = 3.5 Hz, 1H, 11-CH), 7.07 – 7.30 (m, 4H, 13-/15-/16/-17-CH). **^{19}F NMR** (282 MHz, $CDCl_3$): δ 66.0 (dm, $^2J_{F,F}$ = 147.2 Hz, 4F, 10-SF_5), 80.5 (qn, $^2J_{F,F}$ = 147.2 Hz, 1F, 10-SF_5).

Octy 3-hydroxy-3-(3-methoxyphenyl)-2-(pentafluorosulfanyl)propanoate (2l/3l)

According to the general procedure, octyl 2-(pentafluorosulfanyl)acetate (80 mg, 0.27 mmol, 1.0 equiv) was reacted with 3-methoxybenzaldehyd (37 mg, 0.27 mmol, 1.0 equiv). Yield: 143 mg (crude, portion of aldol products: 35%; *syn*/*anti*-ratio 88:12 (^{19}F NMR)). The crude product was purified by column chromatography (silica gel, toluene/diethyl ether, 99:1) to give a colorless oil. Yield: 31 mg (26%, *syn*/*anti*-ratio 99:1 (^{19}F NMR)).

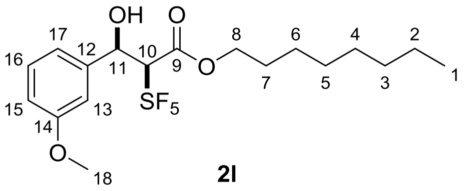

2l

^{1}H NMR (300 MHz, $CDCl_3$): *δ* 0.89 (t, $^{3}J_{H,H}$ = 6.9 Hz, 3H, 1-CH_3), 1.06 – 1.43 (m, 12H, 2-/3-/4-/5-/6-/7-CH_2), 2.65 (s, 1H, OH), 3.76 – 3.92 (m, 2H, 8-CH_2), 3.80 (s, 3H, 18-CH_3), 4.77 (dqn, $^{3}J_{H,H}$ = 9.4 Hz, $^{3}J_{H,F}$ = 5.9 Hz, 1H, 10-CH), 5.41 (d, $^{3}J_{H,H}$ = 9.5 Hz, 1H, 11-CH), 6.86 (ddd, $^{3}J_{H,H}$ = 8.2 Hz, $^{4}J_{H,H}$ = 2.6 Hz, $^{4}J_{H,H}$ = 1.0 Hz, 1H, 15-CH), 6.90 – 6.98 (m, 2H, 13-/17-CH), 7.21 – 7.30 (m, 1H, 16-CH). **^{13}C NMR** (100 MHz, $CDCl_3$): *δ* 14.2 (q, C-1), 22.8 (t, C-2), 25.7 (t, C-6), 28.0 (t, C-7), 29.2, 29.2 (t, C-4/-5), 31.9 (t, C-3), 55.4 (q, C-18), 66.6 (t, C-8), 73.6 (dqn, $^{3}J_{C,F}$ = 2.4 Hz, C-11), 90.1 (dqn, $^{2}J_{C,F}$ = 8.4 Hz, C-10), 113.2 (d, C-13), 115.1 (d, C-15), 120.0 (d, C-17), 129.9 (d, C-16), 139.8 (s, C-12), 159.9 (s, C-14), 163.7 (qn, $^{3}J_{C,F}$ = 3.1 Hz, C-9). **^{19}F NMR** (282 MHz, $CDCl_3$): *δ* 66.8 (dm, $^{2}J_{F,F}$ = 146.6 Hz, 4F, 10-SF_5), 81.6 (qn, $^{2}J_{F,F}$ = 146.8 Hz, 1F, 10-SF_5). **MS-ES(+)-EM**: *m/z* calcd. for $C_{18}H_{27}F_5O_4SNa^+$ 457.1442; found: 457.1446 $[M+Na]^+$.

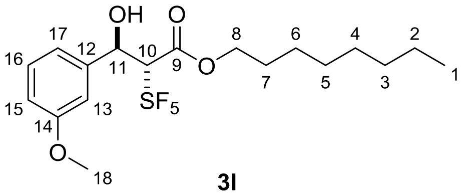

3l

^{1}H NMR (300 MHz, $CDCl_3$): *δ* 0.89 (t, $^{3}J_{H,H}$ = 6.9 Hz, 3H, 1-CH_3), 1.06 – 1.43 (m, 12H, 2-/3-/4-/5-/6-/7-CH_2), 1.60 (s, 1H, OH), 3.80 (s, 3H, 18-CH_3), 4.08 – 4.14 (m, 2H, 8-CH_2), 4.66 (dqn, $^{3}J_{H,F}$ = 6.4 Hz, $^{3}J_{H,H}$ = 3.3 Hz, 1H, 10-CH), 5.47 (d, $^{3}J_{H,H}$ = 3.3 Hz, 1H, 11-CH), 6.86 (ddd, $^{3}J_{H,H}$ = 8.2 Hz, $^{4}J_{H,H}$ = 2.6 Hz, $^{4}J_{H,H}$ = 1.0 Hz, 1H, 15-CH), 6.90 – 6.98 (m, 2H, 13-/17-CH), 7.21 – 7.30 (m, 1H, 16-CH). **^{19}F NMR** (282 MHz, $CDCl_3$): *δ* 65.9 (dm, $^{2}J_{F,F}$ = 146.4 Hz, 4F, 10-SF_5), 80.4 (qn, $^{2}J_{F,F}$ = 146.4 Hz, 1F, 10-SF_5).

Octyl 3-(2-fluorophenyl)-3-hydroxy-2-(pentafluorosulfanyl)propanoate (2m/3m)

According to the general procedure, octyl 2-(pentafluorosulfanyl)acetate (80 mg, 0.27 mmol, 1.0 equiv) was reacted with 2-fluorbenzaldehyde (34 mg, 0.27 mmol, 1.0 equiv). Yield: 138 mg (crude, portion of aldol products: 69%; *syn*/*anti*-ratio 83:17 (^{19}F NMR)). The crude product was purified by column chromatography (silica gel, toluene/diethyl ether, 99:1) to give a colorless oil. Yield: 62 mg (56%, *syn*/*anti*-ratio 90:10 (^{19}F NMR)).

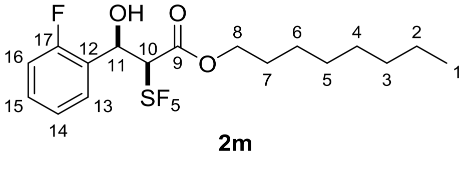

2m

^{1}H NMR (300 MHz, $CDCl_3$): *δ* 0.88 (t, $^{3}J_{H,H}$ = 6.9 Hz, 3H, 1-CH_3), 1.11 – 1.34 (m, 10H, 2-/3-/4-/5-/6-CH_2), 1.38 – 1.51 (m, 2H, 7-CH_2), 3.16 (s, 1H, OH), 3.84 – 3.99 (m, 2H, 8-CH_2), 4.96 (dqn, $^{3}J_{H,H}$ = 8.3 Hz, $^{3}J_{H,F}$ = 5.9 Hz, 1H, 10-CH), 5.68 (d, $^{3}J_{H,H}$ = 8.3 Hz, 1H, 11-CH), 6.99 – 7.10 (m, 1H, 16-CH), 7.12 – 7.21 (m, 1H, 14-CH), 7.27 – 7.39 (m, 1H, 15-CH), 7.40 – 7.48 (m, 1H, 13-CH). **^{13}C NMR** (75 MHz, $CDCl_3$): *δ* 14.2 (q, C-1), 22.8 (t, C-2), 25.7 (t, C-6), 28.0 (t, C-7), 29.1, 29.2 (t, C-4/-5), 31.9 (t, C-3), 66.9 (t, C-8), 68.3 – 68.6 (m, C-11), 88.2 (dqn, $^{2}J_{C,F}$ = 8.6 Hz, $^{4}J_{C,F}$ = 2.3 Hz, C-10), 116.0 (dd, $^{2}J_{C,F}$ = 21.8 Hz, C-16), 124.7 (dd, $^{4}J_{C,F}$ = 3.5 Hz, C-14), 125.5 – 125.8 (m, C-12), 129.3 (dd, $^{3}J_{C,F}$ = 3.5 Hz, C-13), 130.9 (dd, $^{3}J_{C,F}$ = 8.6 Hz, C-15), 160.0 (d, $^{1}J_{C,F}$ = 248.2 Hz, C-17), 164.3 (qn, $^{3}J_{C,F}$ = 3.1 Hz, C-9). **^{19}F NMR** (282 MHz, $CDCl_3$): *δ* [ppm] -117.1 (ddd, $^{3}J_{H,F}$ = 10.7 Hz, $^{4}J_{H,F}$ = 7.3 Hz, $^{4}J_{H,F}$ = 5.3 Hz, 1F, 17-CF), 67.7 (d, $^{2}J_{F,F}$ = 147.5 Hz, 4F, 10-SF_5), 81.2 (qn, $^{2}J_{F,F}$ = 146.6 Hz, 1F, 10-SF_5). **MS-ES(+)-EM**: *m*/*z* calcd. for $C_{17}H_{24}F_6O_3SNa^+$ 445.1243; found: 445.1252 $[M+Na]^+$.

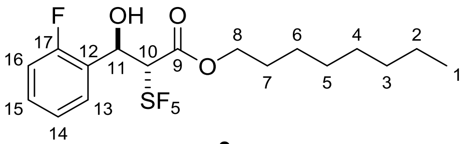

3m

^{1}H-NMR (300 MHz $CDCl_3$): *δ* 0.89 (t, $^{3}J_{H,H}$ = 6.9 Hz, 3H, 1-CH_3), 1.06 – 1.36 (m, 10H, 2-/3-/4-/5-/6-CH_2), 1.37 – 1.54 (m, 2H, 7-CH_2), 1.63 (s, 1H, OH), 3.99 – 4.21 (m, 2H, 8-CH_2), 4.75 (dqn, $^{3}J_{H,F}$ = 6.3 Hz, $^{3}J_{H,H}$ = 3.3 Hz, 1H, 10-CH), 5.75 (d, $^{3}J_{H,H}$ = 3.3 Hz, 1H, 11-CH), 6.99 – 7.10 (m, 1H, 16-CH), 7.12 – 7.21 (m, 1H, 14-CH), 7.27 – 7.39 (m, 1H, 15-CH), 7.40 – 7.48 (m, 1H, 13-CH). **^{19}F NMR** (282 MHz, $CDCl_3$): *δ* -118.4 – -118.2 (m, 1F, 17-CF), 66.1 (d, $^{2}J_{F,F}$ = 148.2 Hz, 4F, 10-SF_5), 80.0 (qn, $^{2}J_{F,F}$ = 147.2 Hz, 1F, 10-SF_5). See spectra of an authentic sample.[1]

Octyl 3-hydroxy-2-(pentafluorosulfanyl)butanoate (2q/3q)

According to the general procedure, octyl 2-(pentafluorosulfanyl)acetate (80 mg, 0.27 mmol, 1.0 equiv) was reacted with acetaldehyde (16 mg, 0.27 mmol, 1.0 equiv). Yield: 54 mg (crude, portion of aldol products: 58%; *syn*/*anti*-ratio 87:13 (^{19}F NMR)). The crude product was purified by column chromatography (silica gel, toluene/diethyl ether, 99:1) to give a colorless oil. Yield: 34 mg (37%, *syn*/*anti*-ratio 89:11 (^{19}F NMR)).

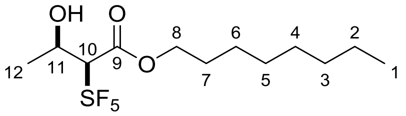

2q

^{1}H NMR (400 MHz, $CDCl_3$): *δ* 0.89 (t, $^{3}J_{H,H}$ = 6.8 Hz, 3H, 1-CH_3), 1.22 – 1.40 (m, 10H, 2-/3-/4-/5-/6-CH_2), 1.35 (d, $^{3}J_{H,H}$ = 6.3 Hz, 3H, 12-CH_3), 1.62 – 1.73 (m, 2H, 7-CH_2), 2.69 (s, 1H, OH), 4.13 – 4.28 (m, 2H, 8-CH_2), 4.40 (dqn, $^{3}J_{H,H}$ = 7.7 Hz, $^{3}J_{H,F}$ = 6.1 Hz, 1H, 10-CH), 4.51 – 4.63

(m, 1H, 11-CH). **^{13}C NMR** (100 MHz, $CDCl_3$): δ 14.2 (q, C-1), 20.8 (dqn, $^4J_{C,F}$ = 2.0 Hz, C-12), 22.8 (t, C-2), 25.8 (t, C-6), 28.2 (t, C-7), 29.2, 29.2 (t, C-4/-5), 31.9 (t, C-3), 67.0 (t, C-8), 67.4 (dqn, $^3J_{C,F}$ = 2.4 Hz, C-11), 90.3 (dqn, $^2J_{C,F}$ = 8.3 Hz, C-10), 164.9 (qn, $^3J_{C,F}$ = 3.2 Hz, C-9). **^{19}F NMR** (282 MHz, $CDCl_3$): δ 67.2 (dm, $^2J_{F,F}$ = 146.2 Hz, 4F, 10-SF_5), 82.0 (qn, $^2J_{F,F}$ = 146.0 Hz, 1F, 10-SF_5). **MS-ES(+)-EM**: *m/z* calcd. for $C_{12}H_{23}F_5O_3SNa^+$ 365.1180; found: 365.1188 $[M+Na]^+$.

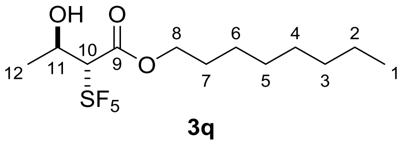

3q

^{1}H NMR (400 MHz, $CDCl_3$): δ 0.89 (t, $^3J_{H,H}$ = 6.8 Hz, 3H, 1-CH_3), 1.22 – 1.40 (m, 10H, 2-/3-/4-/5-/6-CH_2), 1.35 (d, $^3J_{H,H}$ = 6.3 Hz, 3H, 12-CH_3), 1.62 – 1.73 (m, 2H, 7-CH_2), 2.69 (s, 1H, OH), 4.24 – 4.34 (m, 3H, 8-CH_2 and 10-CH), 4.51 – 4.63 (m, 1H, 11-CH). **^{19}F NMR** (282 MHz, $CDCl_3$): δ 66.0 (dm, $^2J_{F,F}$ = 146.5 Hz, 4F, 10-SF_5), 81.1 (qn, $^2J_{F,F}$ = 146.5 Hz, 1F, 10-SF_5).

2-(Pentafluorosulfanyl)acetic acid morpholide (8)

In an oven-dried pressure vessel under argon atmosphere, morpholine (1.1 equiv) was dissolved in dry CH_2Cl_2 (1 mL), and cooled down to −10 °C. Then 2-(pentafluorosulfanyl)acetic acid chloride (**7**) (215 mg, 1.05 mmol, 1.0 equiv) was added dropwise. The mixture was allowed to warm up to room temperature and stirred at this temperature for 15 hours. The solution was then diluted with CH_2Cl_2 (7 mL) and washed with 2 M HCl (5 mL), saturated bicarbonate solution (5 mL), and brine (5 mL). The combined organic phases were dried with $MgSO_4$, and the solvent was evaporated under reduced pressure. The product was isolated as a pure white solid. Additional purification was not needed. Yield: 159 mg (59%). Mp 84 – 85 °C.

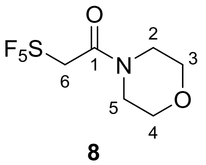

8

^{1}H NMR (300 MHz, $CDCl_3$): δ 3.54 – 3.74 (m, 8H, 2-/3-/4-/5-CH_2), 4.45 (qn, $^3J_{H,F}$ = 7.6 Hz, 2H, 6-CH_2). **^{13}C NMR** (75 MHz, $CDCl_3$): δ 42.7* and 47.1* (t, C-2 and C-5), 66.0* and 66.3* (t, C-3 and C-4), 69.1 (qn, $^2J_{C,F}$ = 7.6 Hz, 2H, C-6), 159.9 (qn, $^3J_{C,F}$ = 4.0 Hz, C-1). **^{19}F NMR** (282 MHz, $CDCl_3$): δ 70.3 (dm, $^2J_{F,F}$ = 147.2 Hz, 4F, 6-SF_5), 80.7 (qn, $^2J_{F,F}$ = 147.2 Hz, 1F, 6-SF_5). **MS-ES(+)-EM**: *m/z* calcd. for $C_6H_{10}F_5NO_2SNa^+$ 278.0245; found: 278.0241 $[M+Na]^+$.

* Because of hindered C-N amide bond rotation at ambient temperature, C-2 and C-5 as well as C-3 and C-4 show different chemical shifts. This phenomenon id known in the literature for other N-acyl morpholides [2].

Attempts to use 2-(pentafluorosulfanyl)acetic acid morpholide (8) as methylene component in aldol reaction with *p*-nitrobenzaldehyde

Varination A:

According to the general procedure, 2-(pentafluorosulfanyl)acetic acid morpholide (**8**) (55 mg, 0.22 mmol, 1.0 equiv) was reacted with *p*-nitrobenzaldehyde. Besides the starting materials, minor amounts of a SF_5-containing compound were found. Yield: 115 mg (crude product, composition: 87% of **8** and 13% of the unknown SF_5-containing compound, ^{19}F NMR spectrum. The product could not be isolated.

Variation B:

In a Young NMR tube under argon, 2-(pentafluorsulfanyl)acetic acid morpholide (**8**, 30 mg, 0.12 mmol, 1.0 equiv) was dissolved in CD_2Cl_2 (0.5 mL), and TMSOTf (40 mg, 0.18 mmol, 1.5 equiv) and triethylamine (18 mg, 0.18 mmol, 1.5 equiv) were added at room temperature. The formation of the (*Z*)-enolate was followed by NMR spectroscopy. After a couple of minutes, when the enolate formation was complete (see NMR spectra at pages S64–S67), *p*-nitobenzaldehyde (19 mg, 0,12 mmol, 1.0 equiv) and $TiCl_4$ (7 mg, 0.04 mmol, 0.3 equiv) were added. After 3 days at room temperature (NMR spectra are shown at pages S68 and S69), the reaction was stopped by addition of ice water (5 mL). The phases were separated and the aqueous phase was extracted with CH_2Cl_2 (4 × 5 mL). The combined organic phases were dried with $MgSO_4$, and the solvent was evaporated under reduced pressure to give 76 mg of a crude mixture. The proposed product could not be isolated, and its structure remains to be further elucidated.

Ketene silylaminal of 2-(pentafluorosulfanyl)acetic acid morpholide

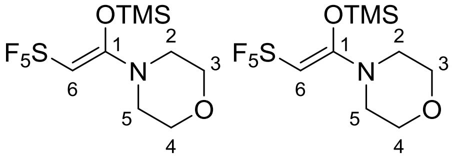

^{1}H NMR (600 MHz, CD_2Cl_2): δ 2.91 (m, 4H, 2-/5-CH_2), 3.69 (m, 4H, 3-/4-CH_2), 5.15 (qn, $^3J_{H,F}$ = 7.0 Hz, 1H, 6-CH). **^{13}C NMR** (150 MHz, CD_2Cl_2): δ 1.3 (q, $SiMe_3$), 48.5 (t, C-2 and C-5), 66.2 (t, C-3 and C-5), 106.1 (qn, $^2J_{C,F}$ = 19.4 Hz, C-6), 156.9 (qn, $^3J_{C,F}$ = 4.8 Hz, C-1). **^{19}F NMR** (566 MHz, CD_2Cl_2): δ 74.1 (dm, 4 F, $^2J_{F,F}$ = 151.6 Hz, SF_5), 91.7 (qn, 1F, $^2J_{F,F}$ = 151.6 Hz, SF_5) (extracted from a crude reaction mixture).

3-Hydroxy-3-(4-nitrophenyl)-2-(pentafluorosulfanyl)propanoic acid morpholide trimethylsilyl aminal (9)

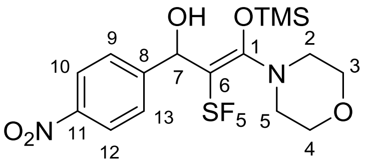

9

^{1}H NMR (600 MHz, CD_2Cl_2): *δ* 0.4 (s, 9H, OTMS), 3.27 (dq, 4H, $^2J_{H,H}$ and $^3J_{H,H}$ = 14.5 Hz, $^3J_{H,H}$ = 7.3 Hz, 2-/5-CH_2), 3.37 (dq, 4H, $^2J_{H,H}$ and $^3J_{H,H}$ = 14.5 Hz, $^3J_{H,H}$ = 7.3 Hz, 3-/4-CH_2), 4.62 (qm, 1H, $^3J_{H,H}$ = 7.2 Hz, $^4J_{H,F}$ = 0.5 Hz, 7-CH), 6.30 (brs, 1H, OH), 7.96 (d, 2H, 9-/13-CH), 8.32 (d, 2H, 10-/12-CH). **^{19}F NMR** (282 MHz, CD_2Cl_2): *δ* 57.6 (dm, $^2J_{F,F}$ = 147.3 Hz, 4F, SF_5), 74.7 (pn, $^2J_{F,F}$ = 147.3 Hz, 1F, SF_5) (extracted from a crude reaction mixture).

References

1. Friese, F.W.; Dreier, A.-L.; Matsnev, A. V.; Daniliuc, C.-G.; Thrasher, J. S.; Haufe, G. *Org. Lett.* **2016**, *18*, 1012-1015.
2. Salman, S. R.; Farrant, R. D.; Glen, R. C.; Lindon, J. C. *Spectroscopy Lett.* **1998**, *31*, 269-274 and references cited therein.

^{1}H NMR spectrum of compound **2a**

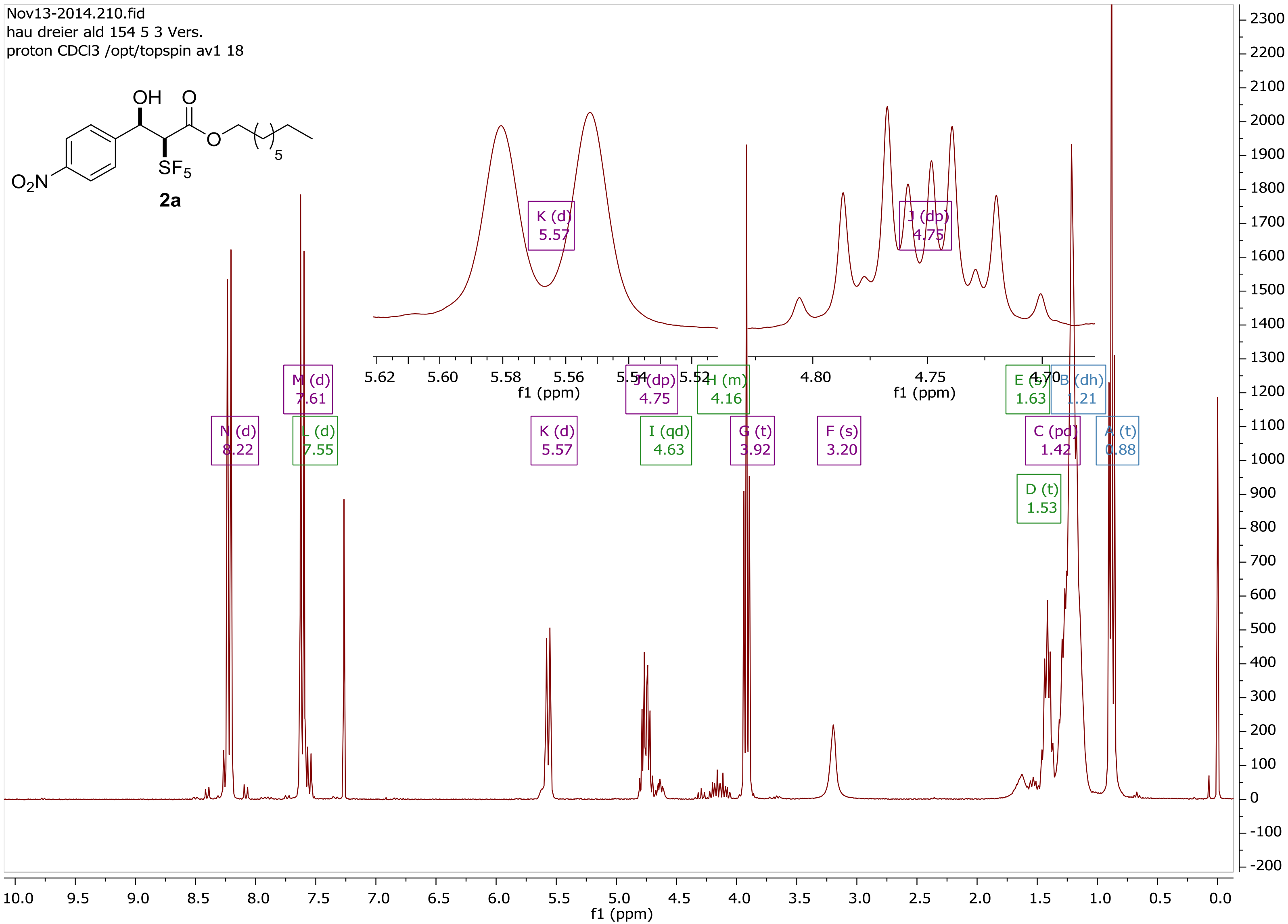

^{13}C NMR spectrum of compound **2a**

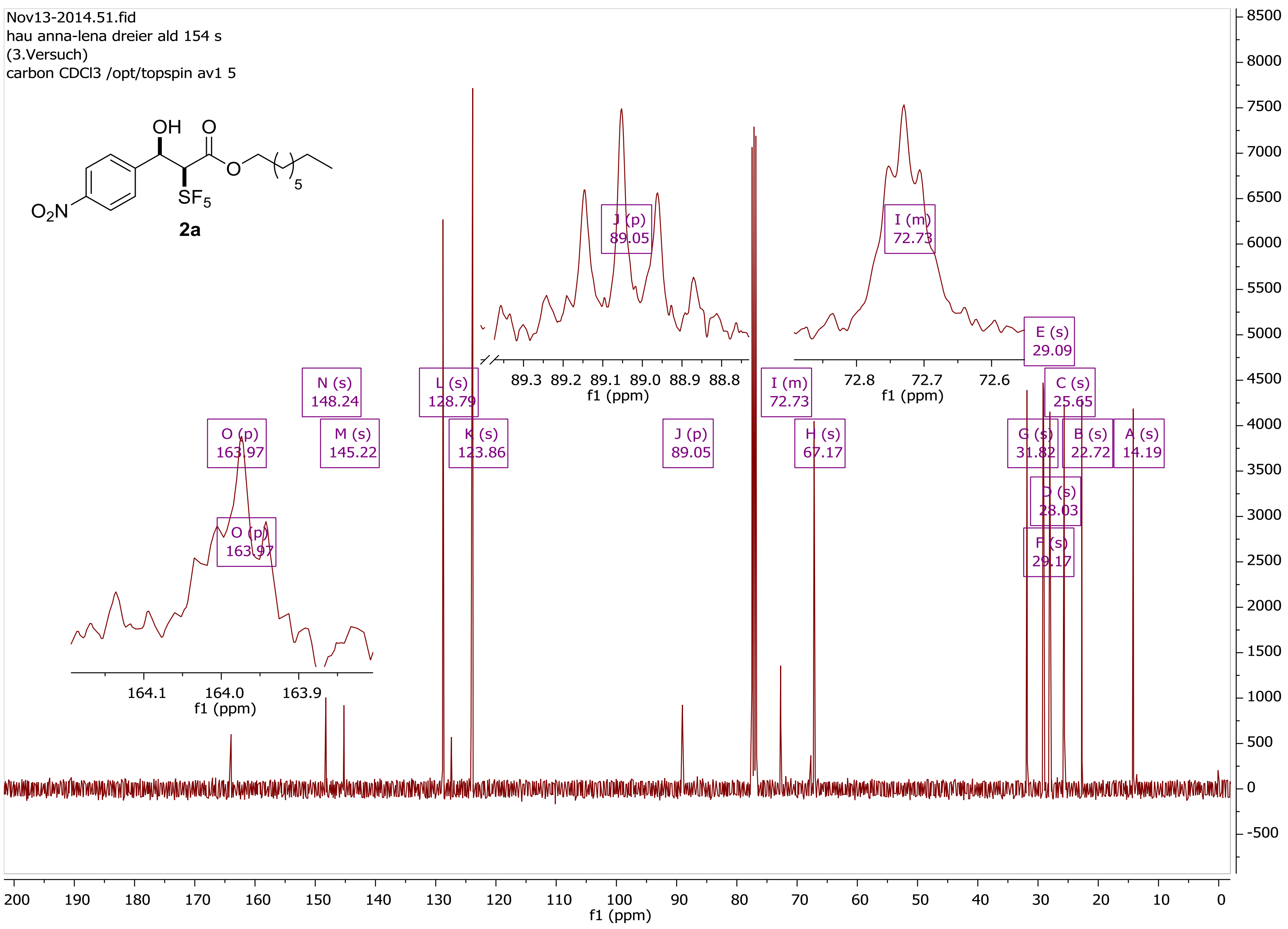

^{19}F NMR spectrum of compound **2a**

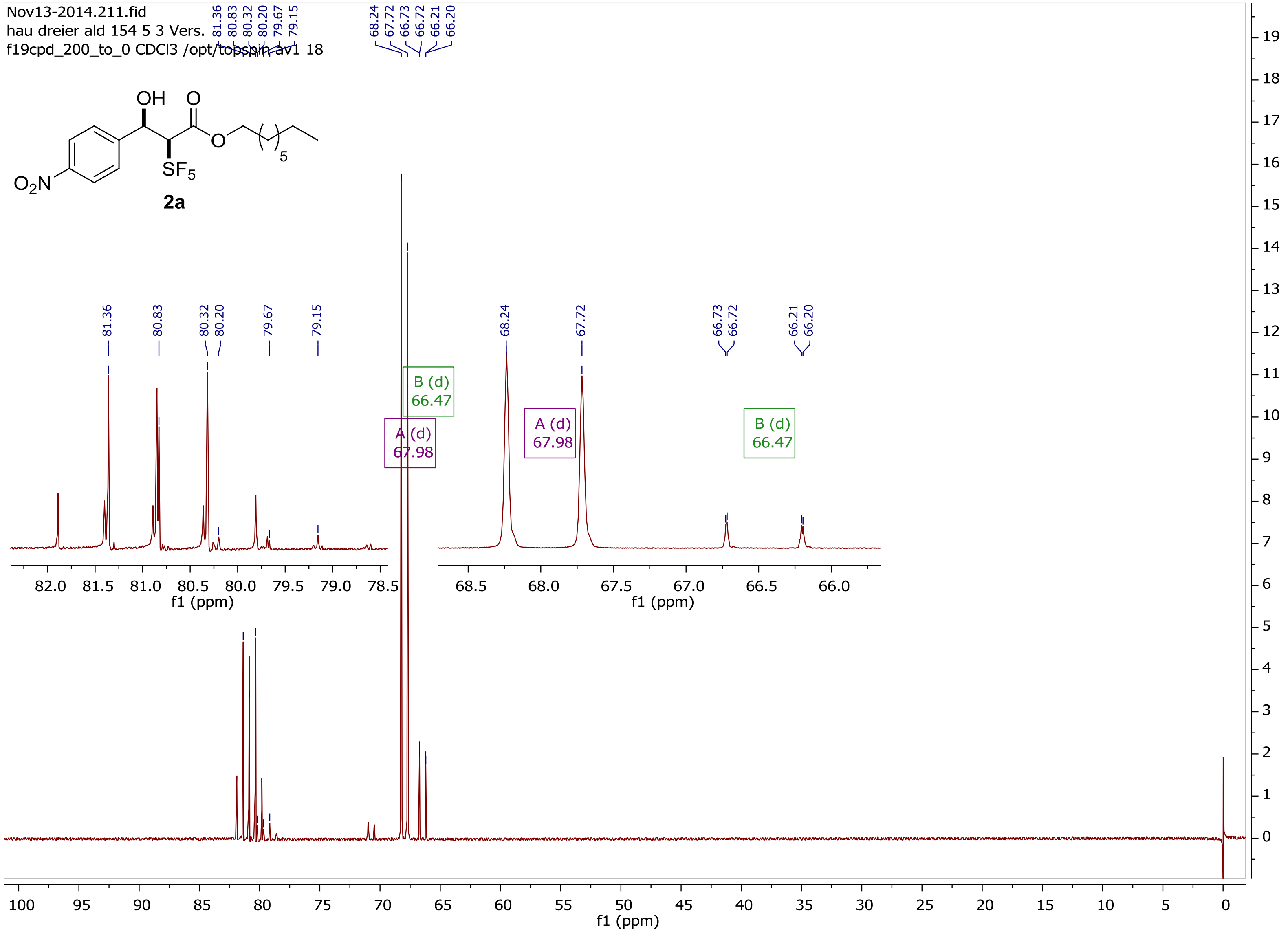

^{1}H NMR spectrum of compound **2b**

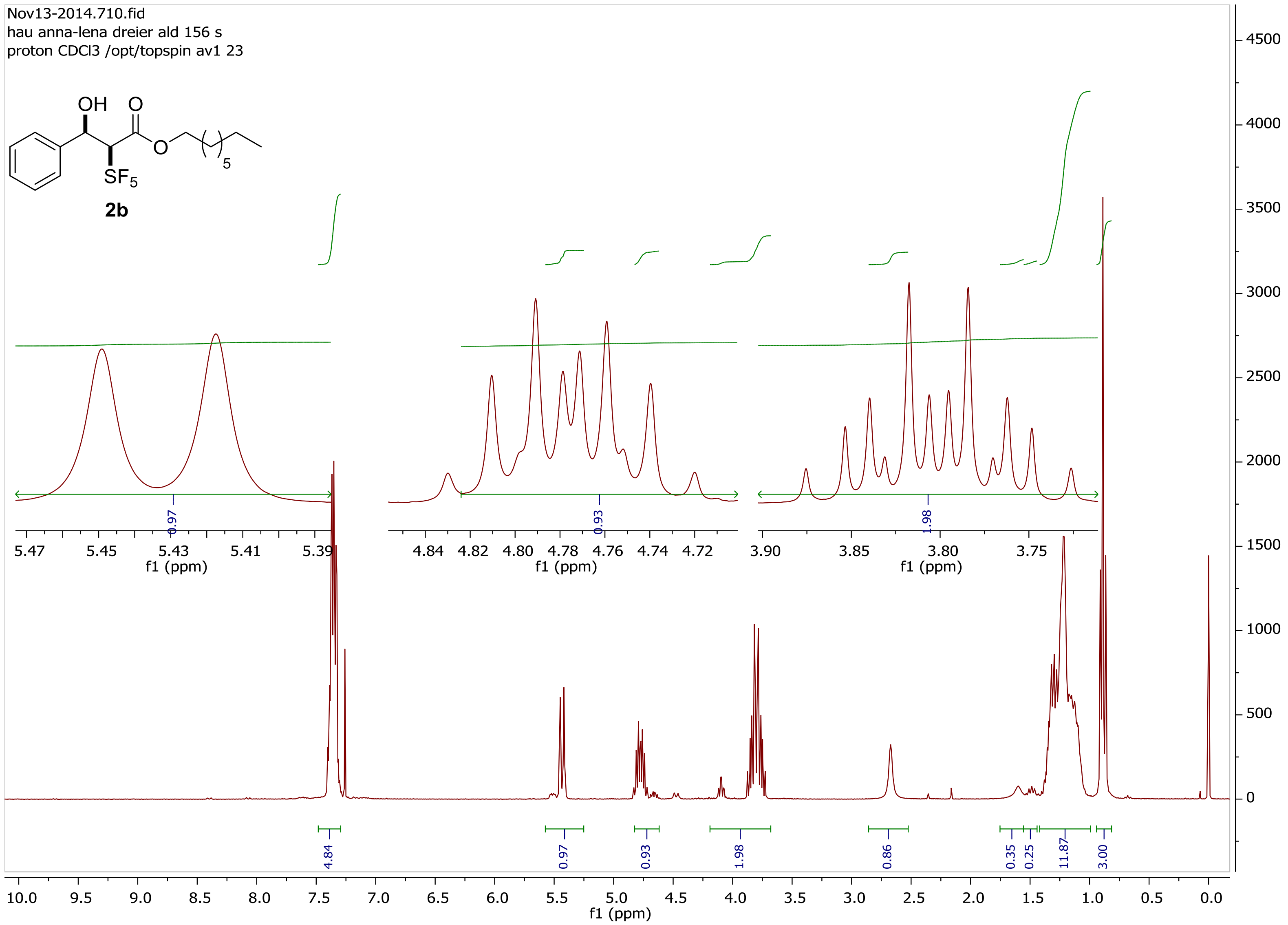

^{13}C NMR spectrum of compound **2b**

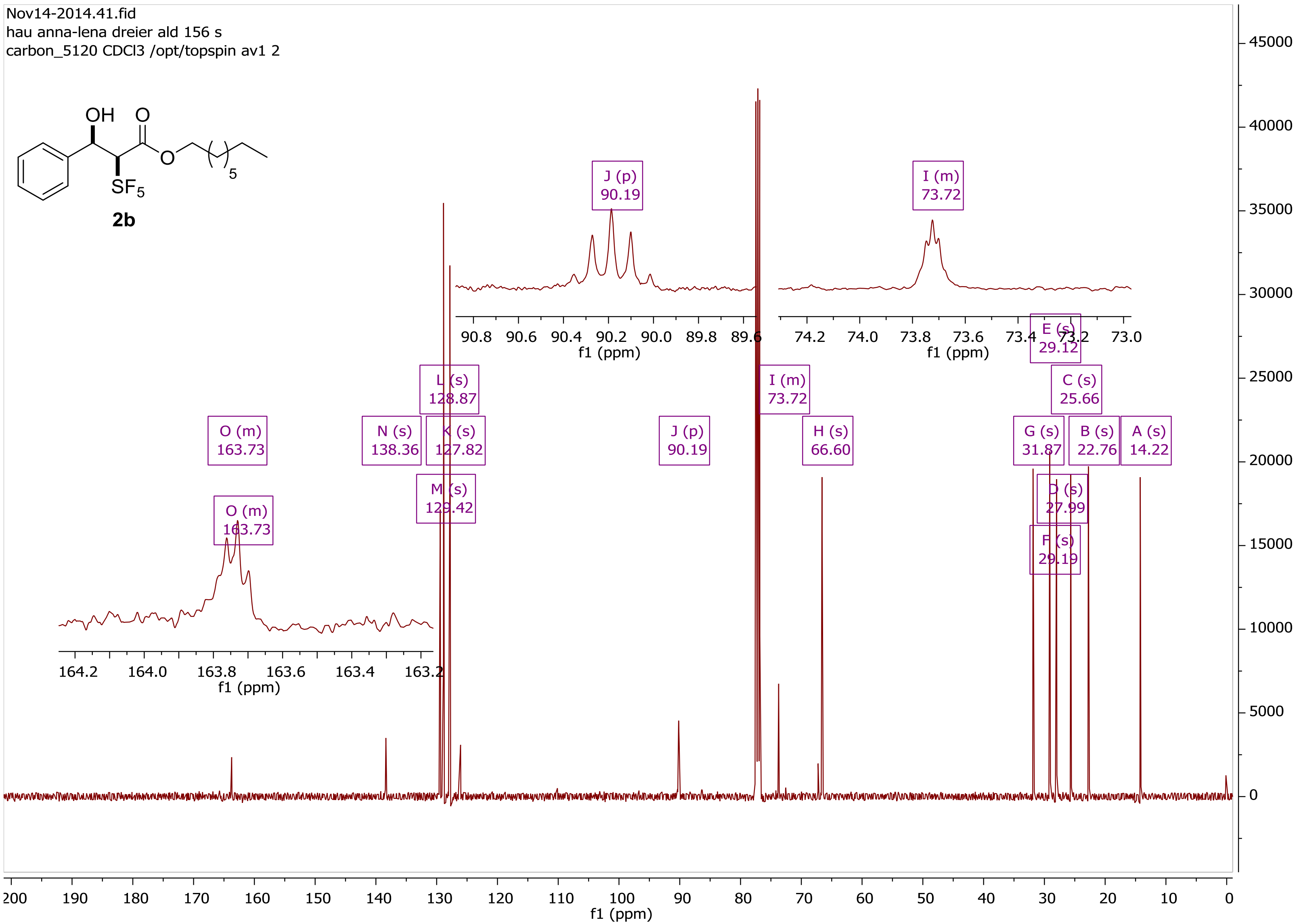

^{19}F NMR spectrum of compound **2b**

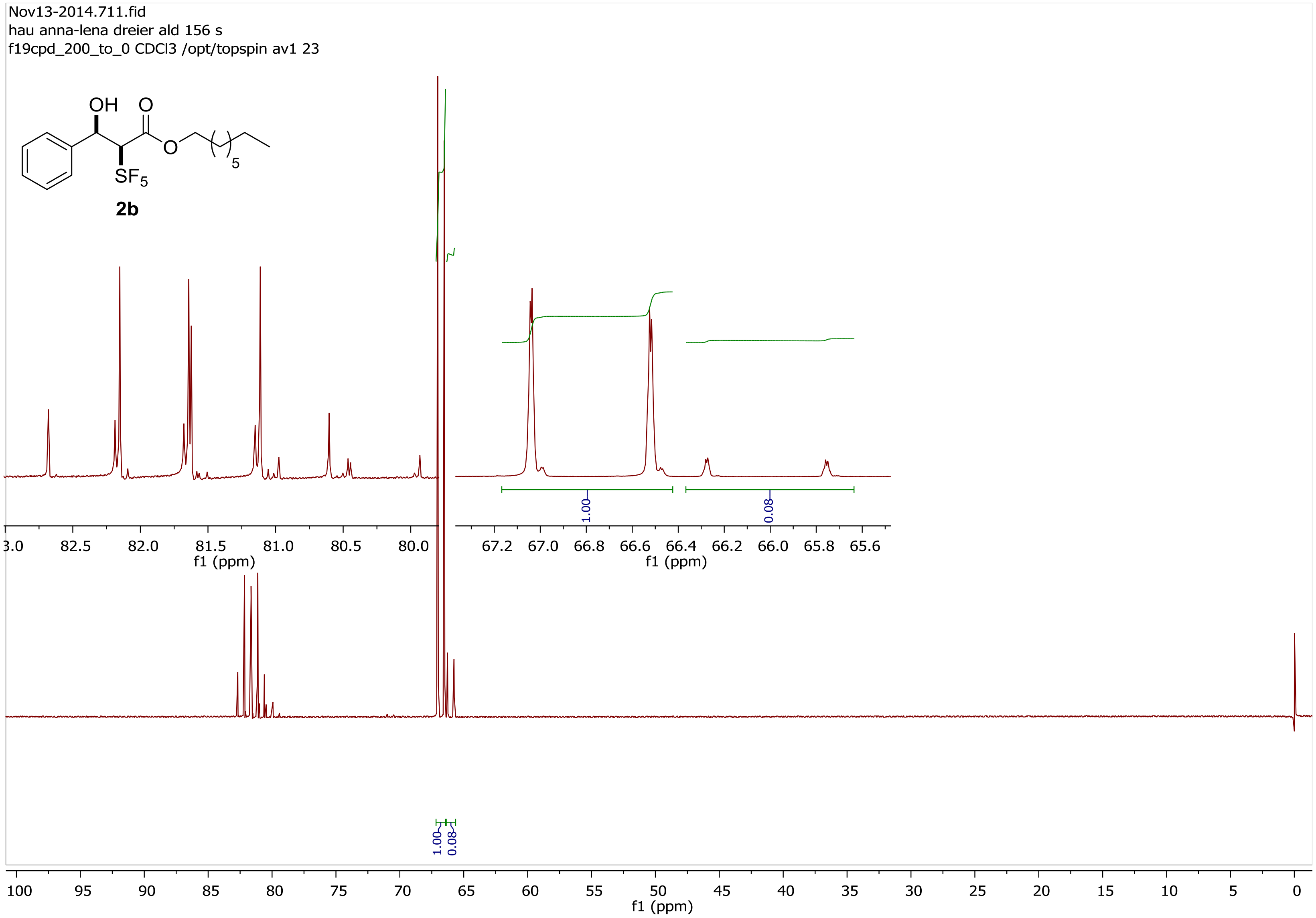

^{1}H NMR spectrum of compound **2c**

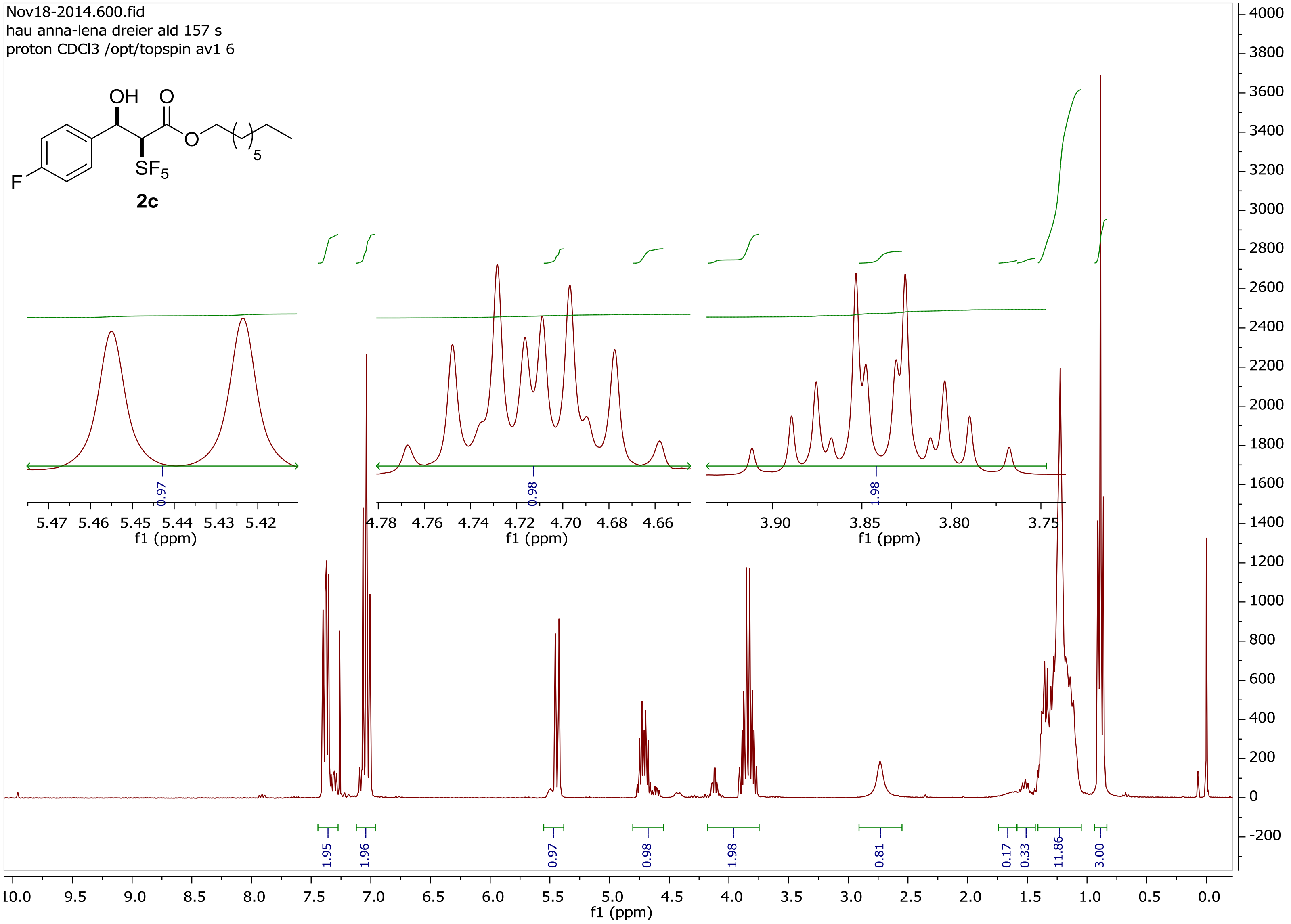

^{13}C NMR spectrum of compound **2c**

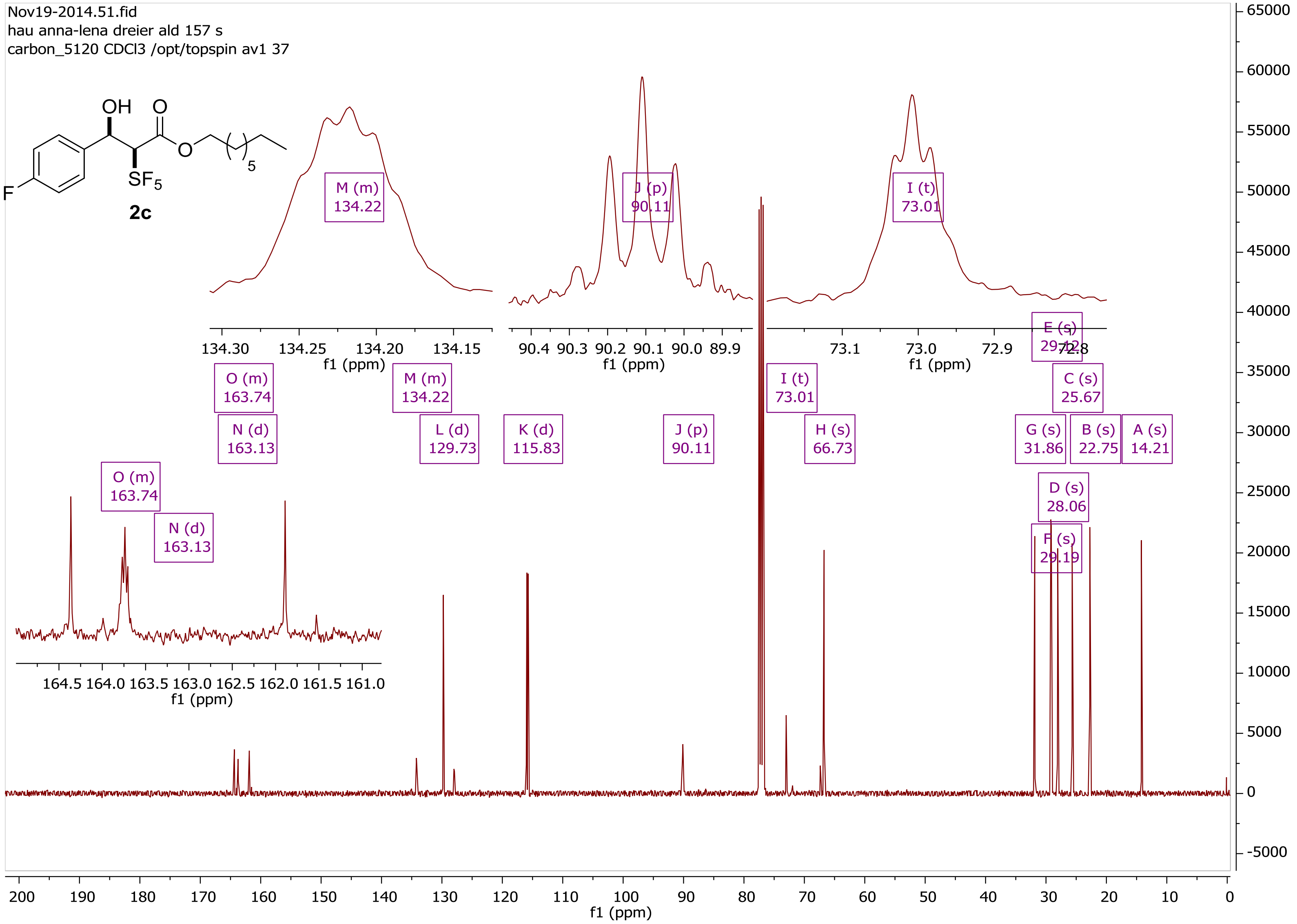

^{19}F NMR spectrum of compound **2c** (positive part)

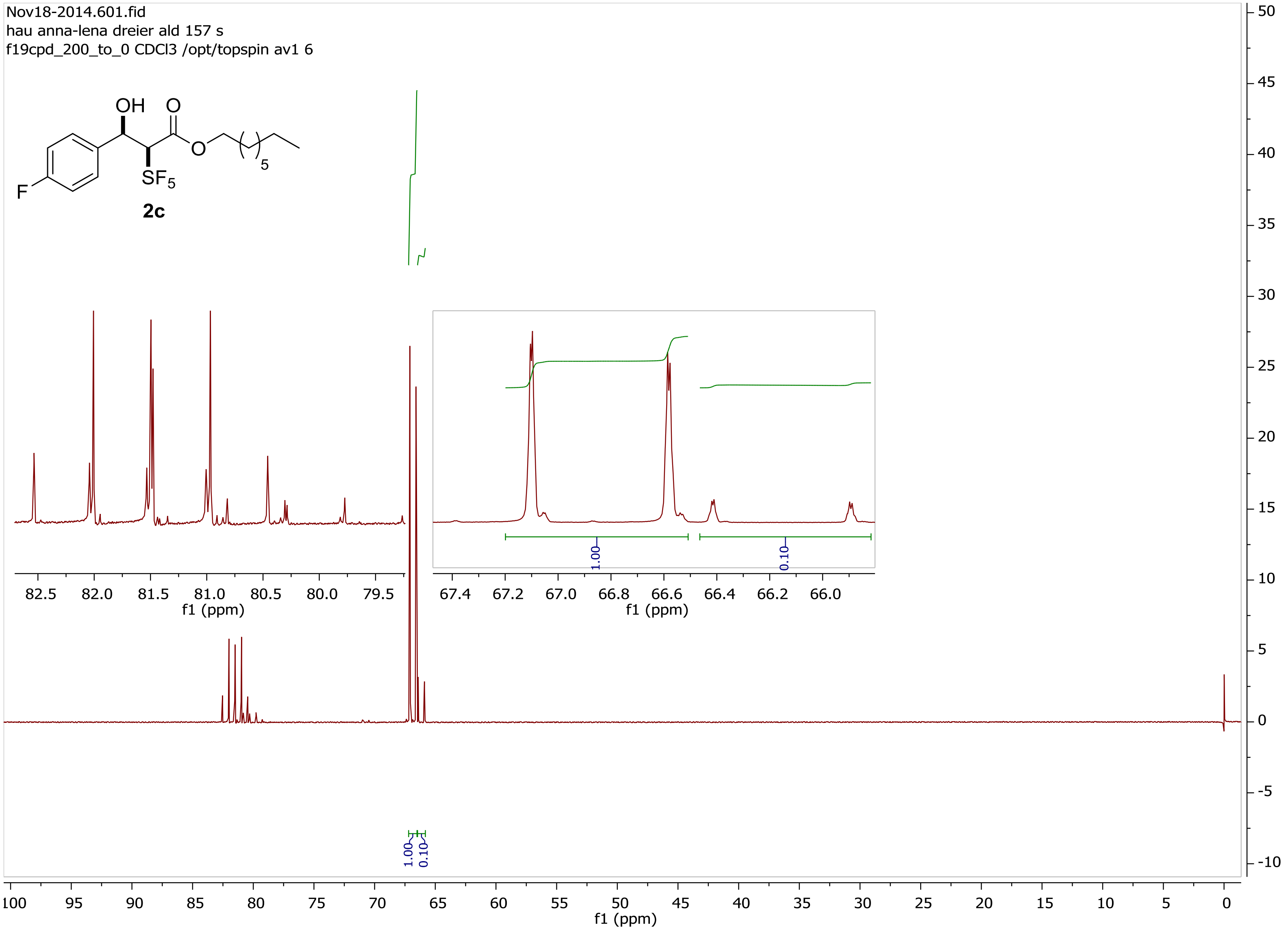

^{19}F NMR spectrum of compound **2c** (negative part)

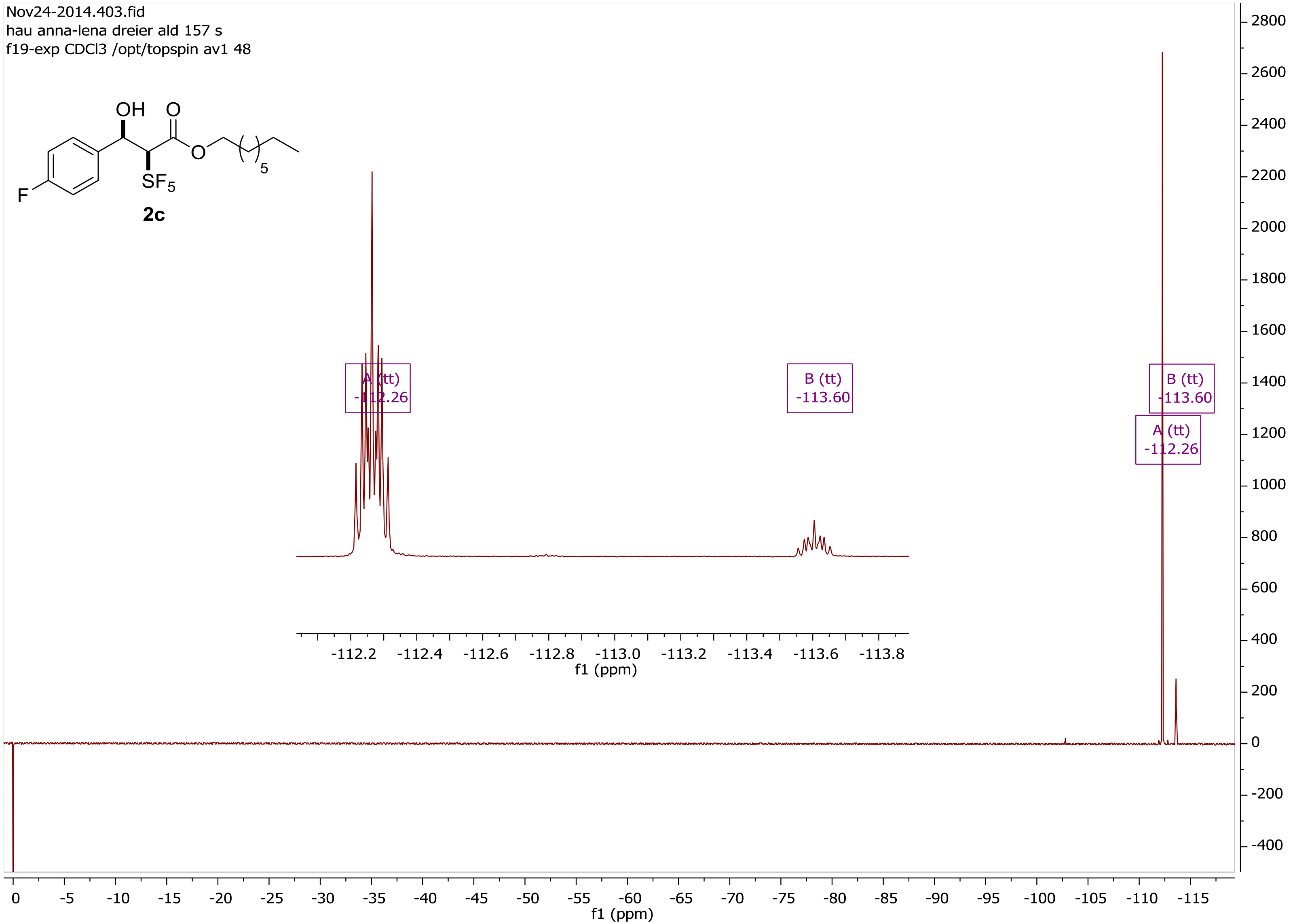

^{1}H NMR spectrum of compound **2d**

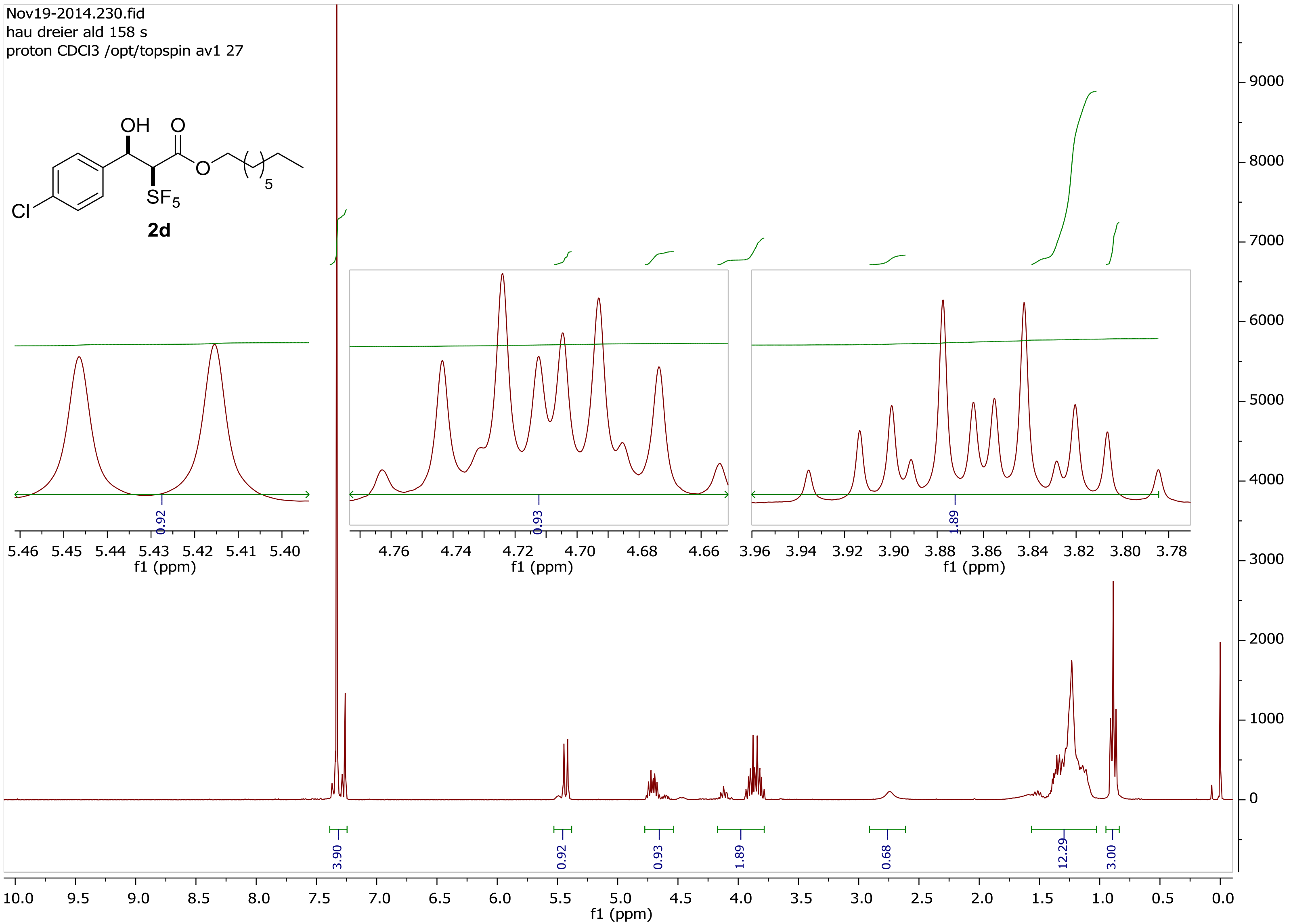

^{13}C NMR spectrum of compound **2d**

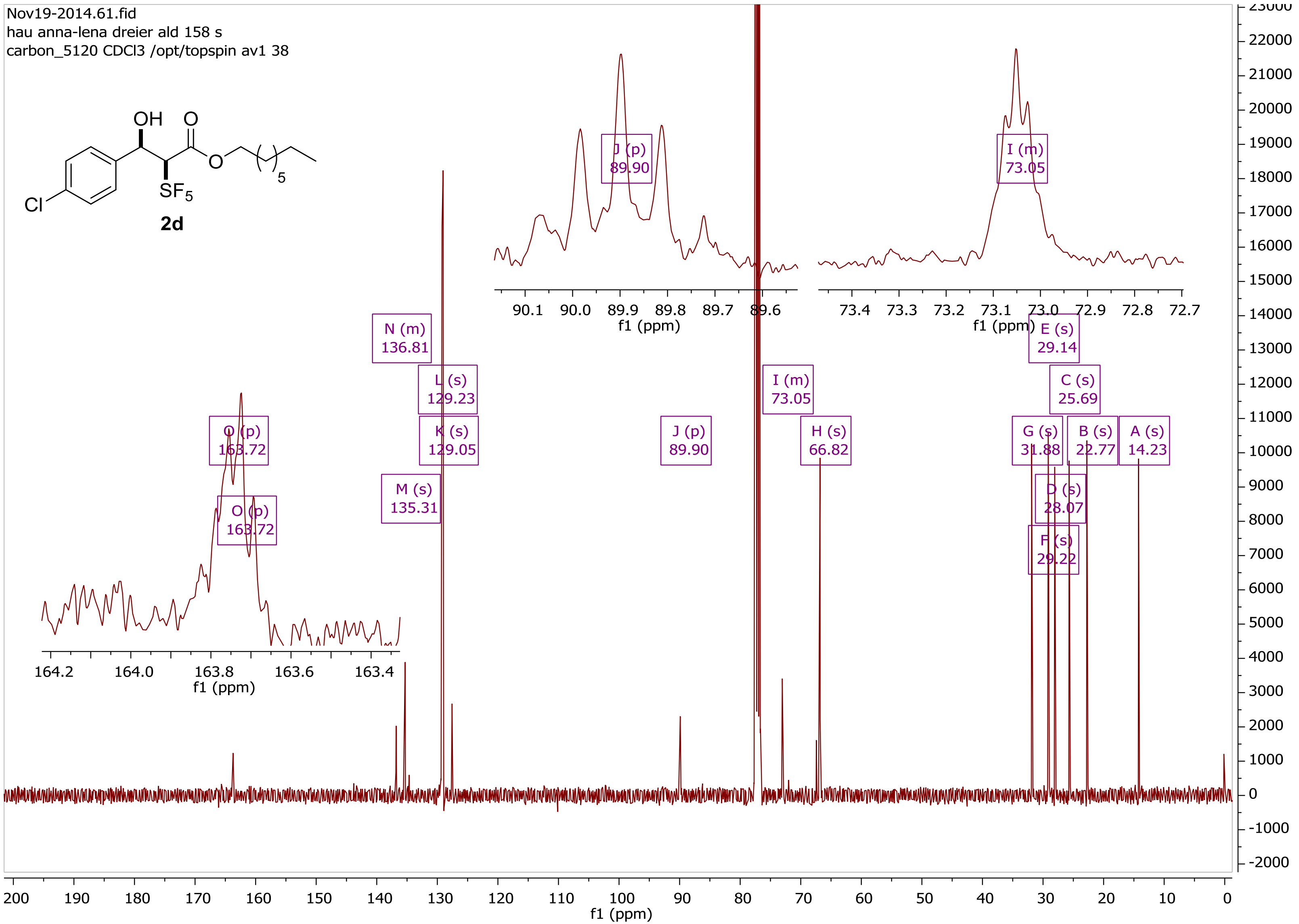

^{19}F NMR spectrum of compound **2d**

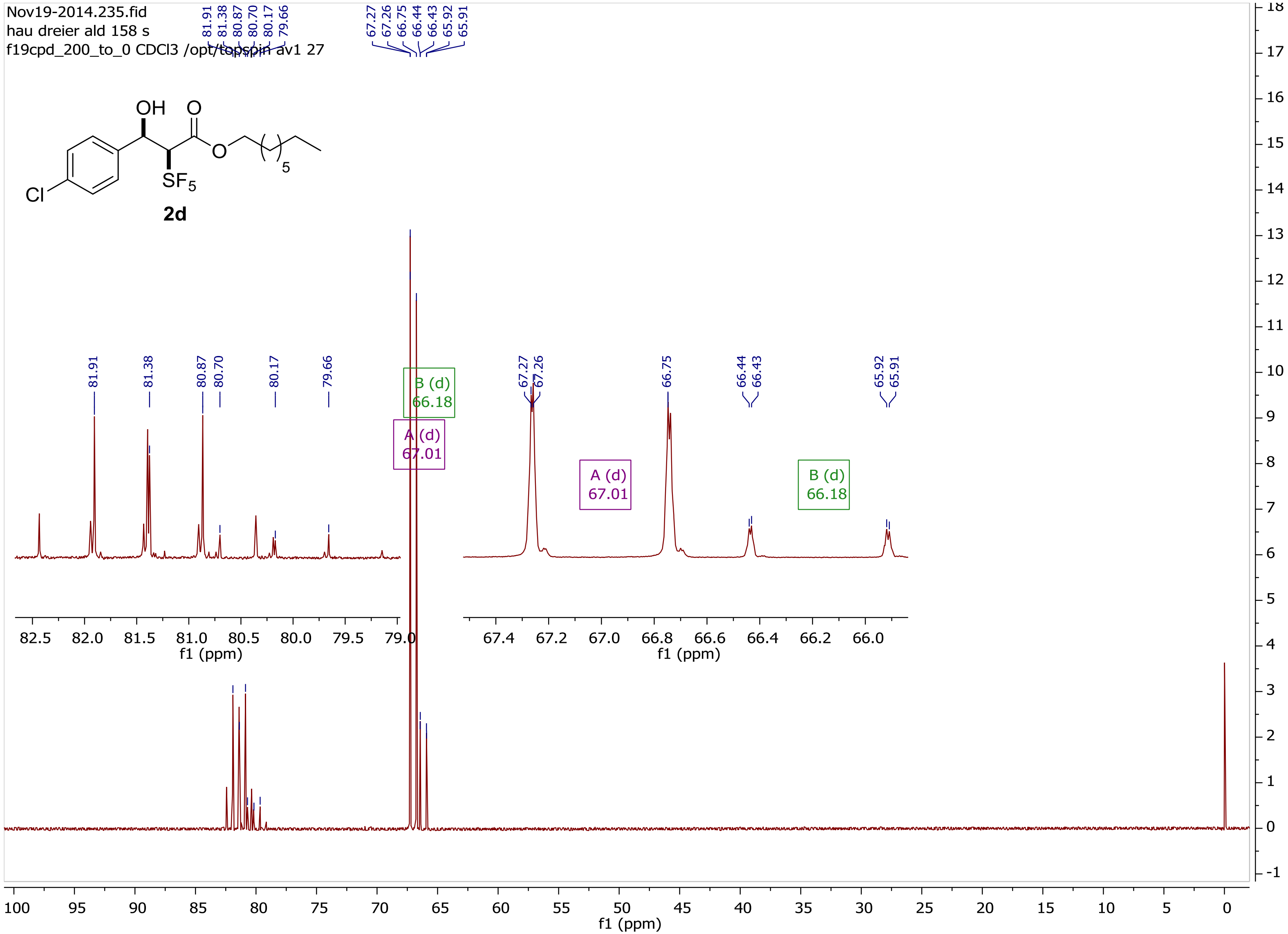

^{1}H NMR spectrum of compound **2e**

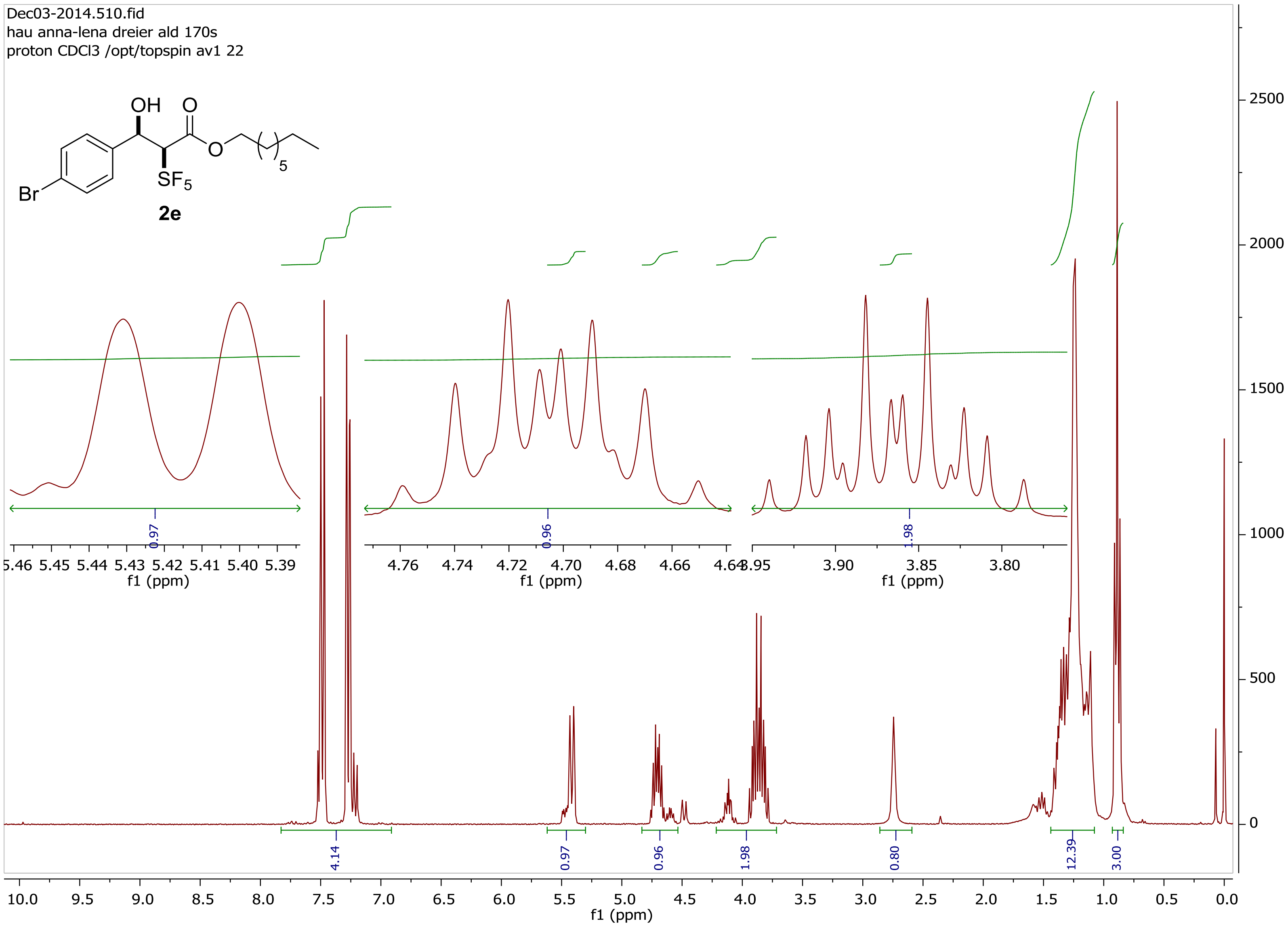

^{13}C NMR spectrum of compound **2e**

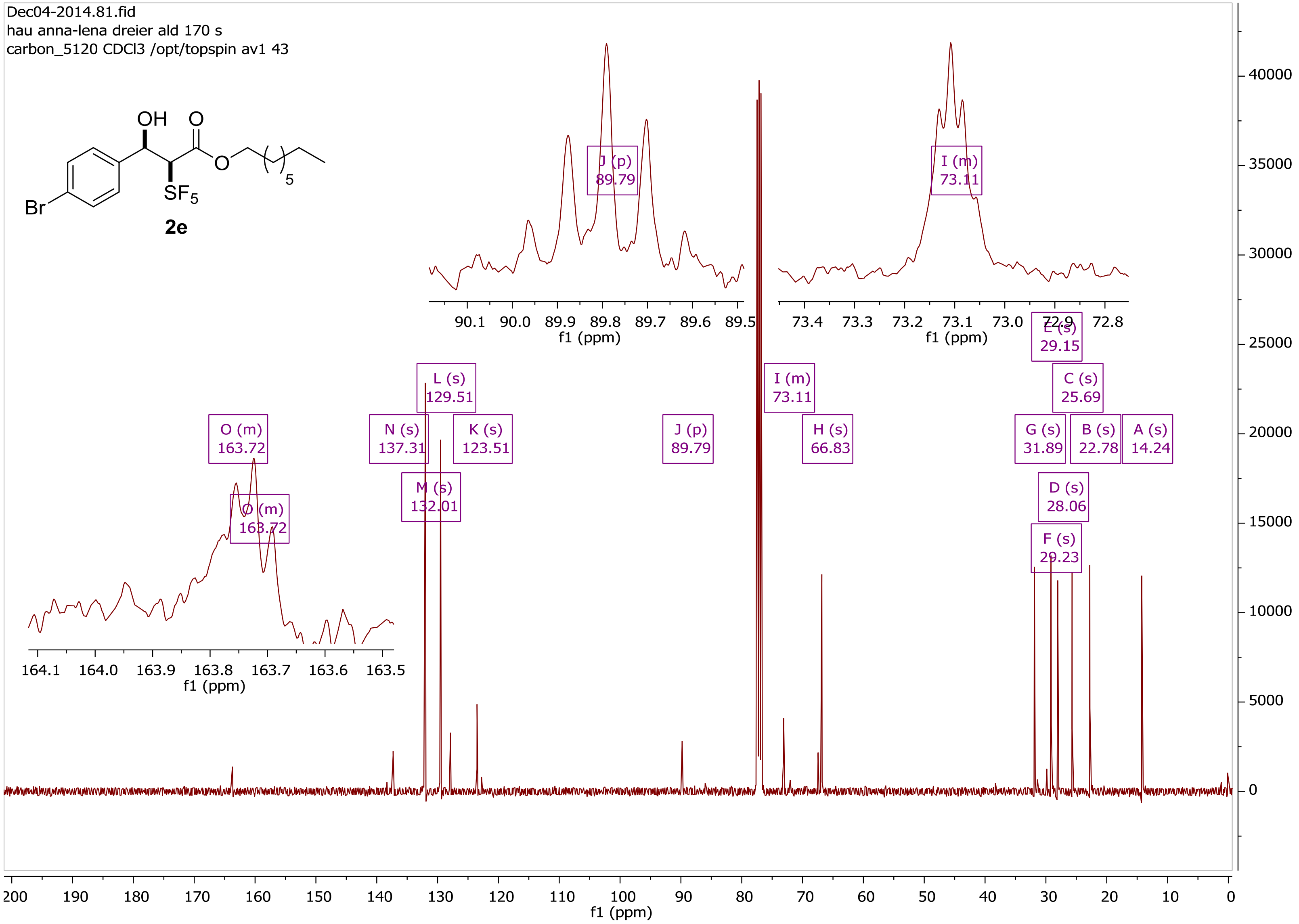

^{19}F NMR spectrum of compound **2e**

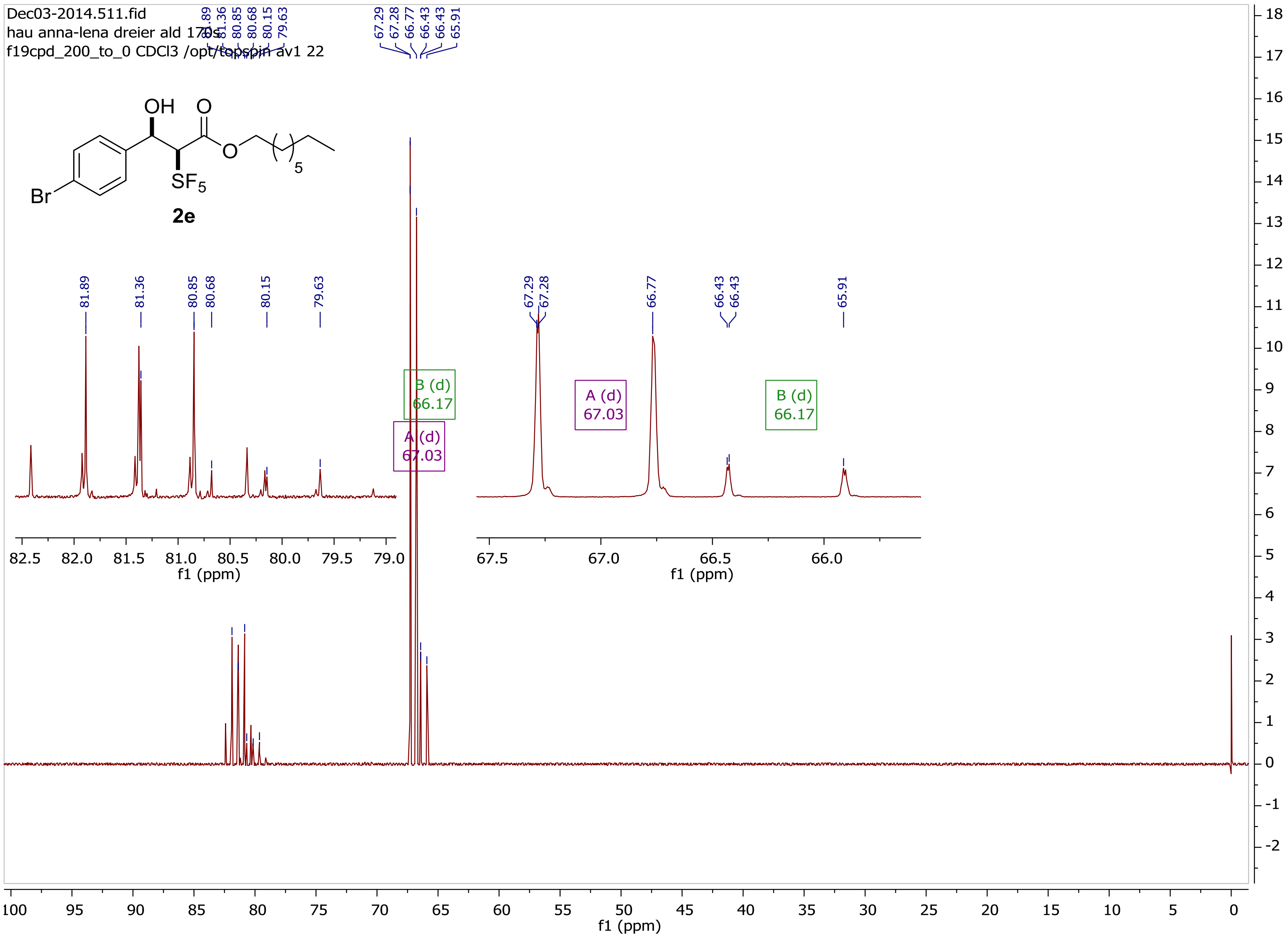

^{1}H NMR spectrum of compound **2f**

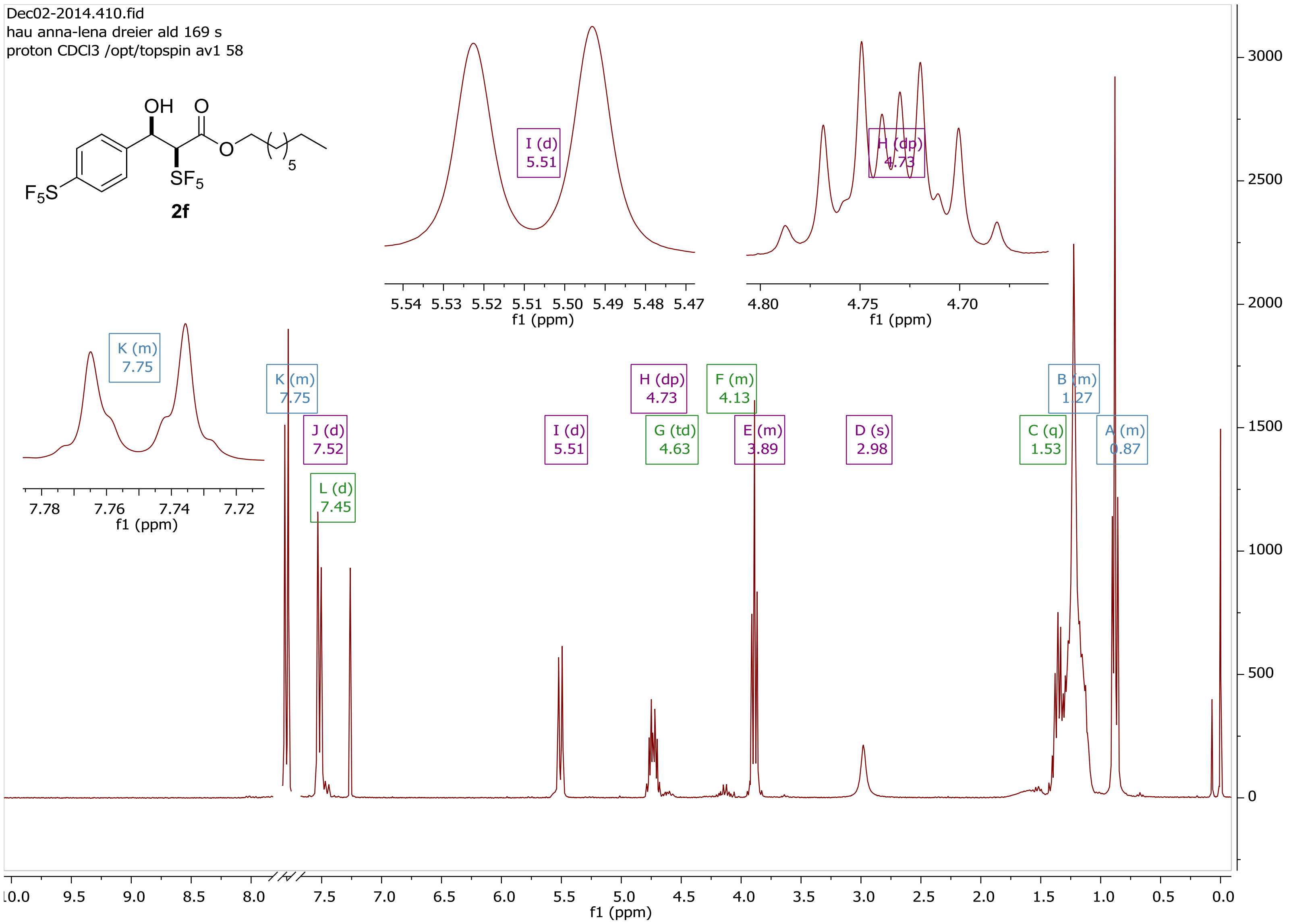

^{13}C NMR spectrum of compound **2f**

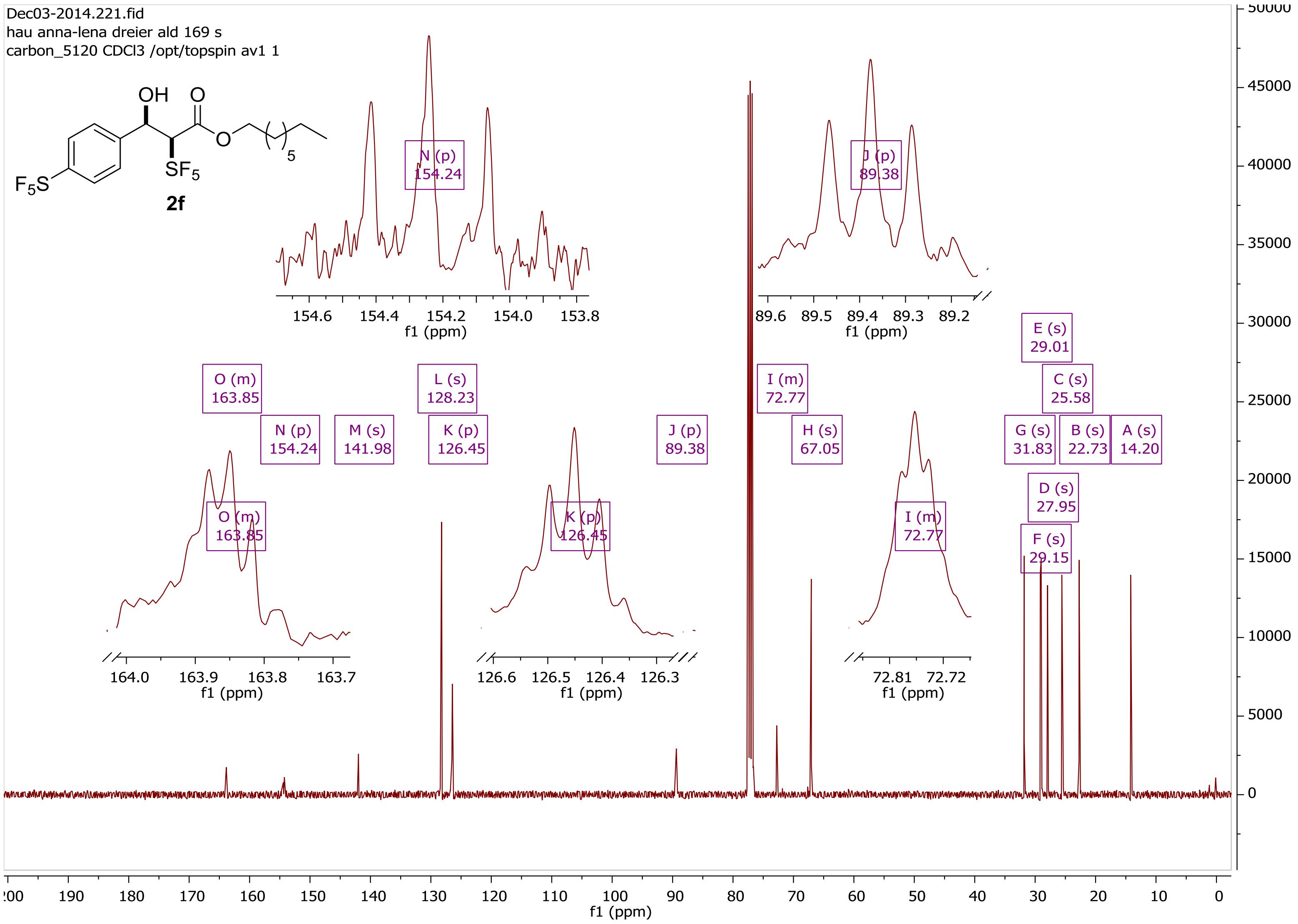

^{19}F NMR spectrum of compound **2f**

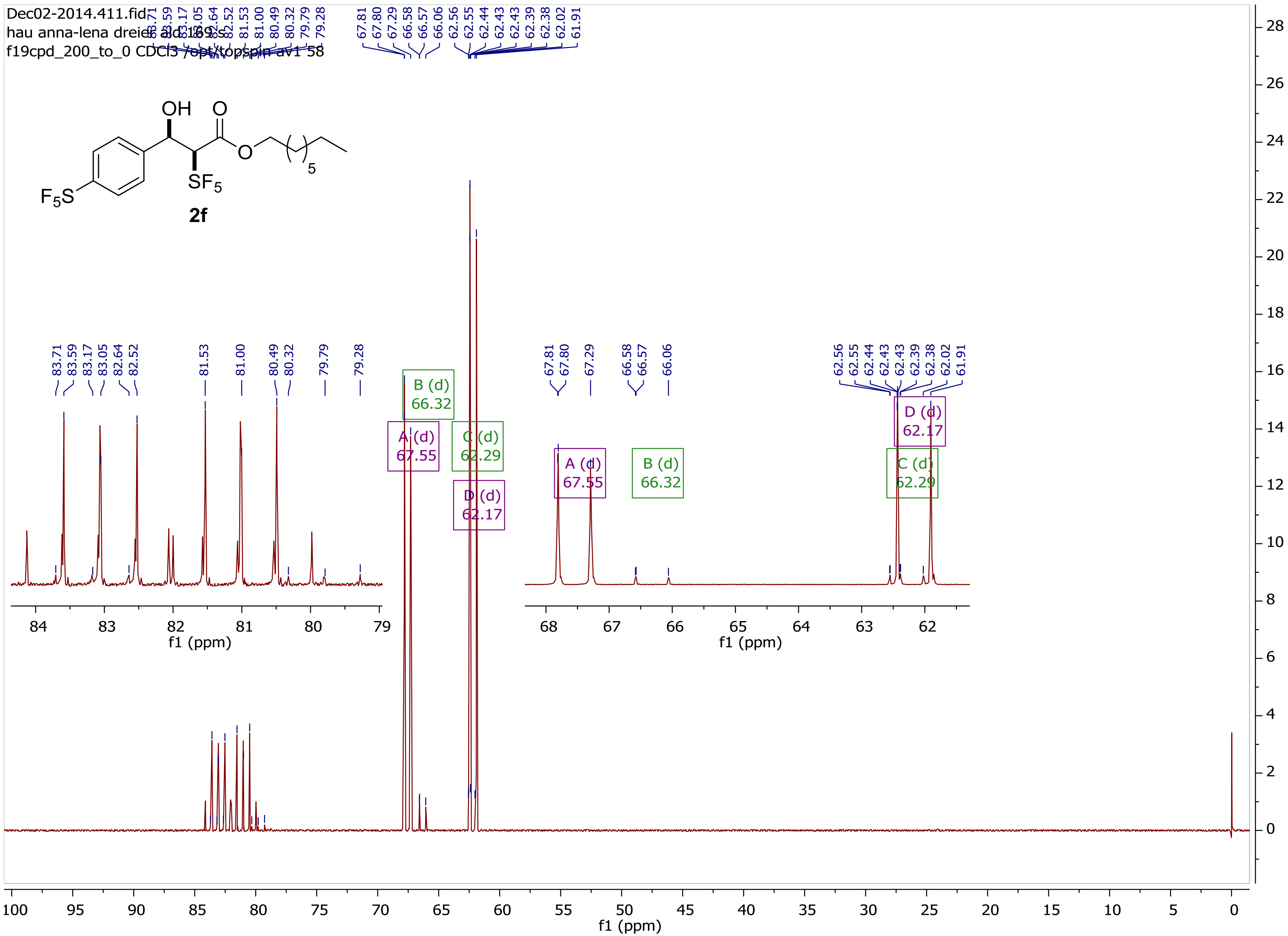

^{1}H NMR spectrum of compound **4g**

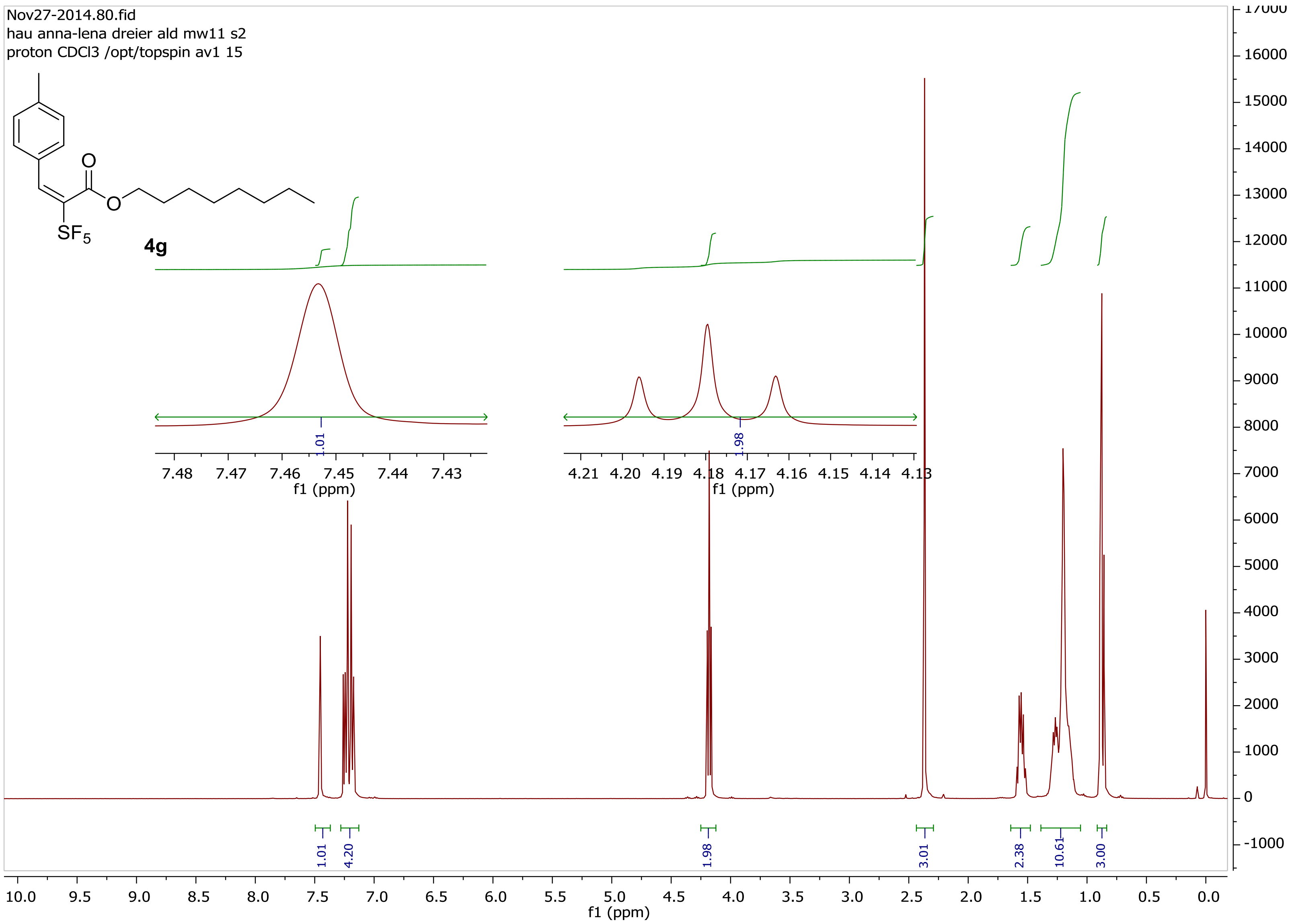

^{13}C NMR spectrum of compound **4g**

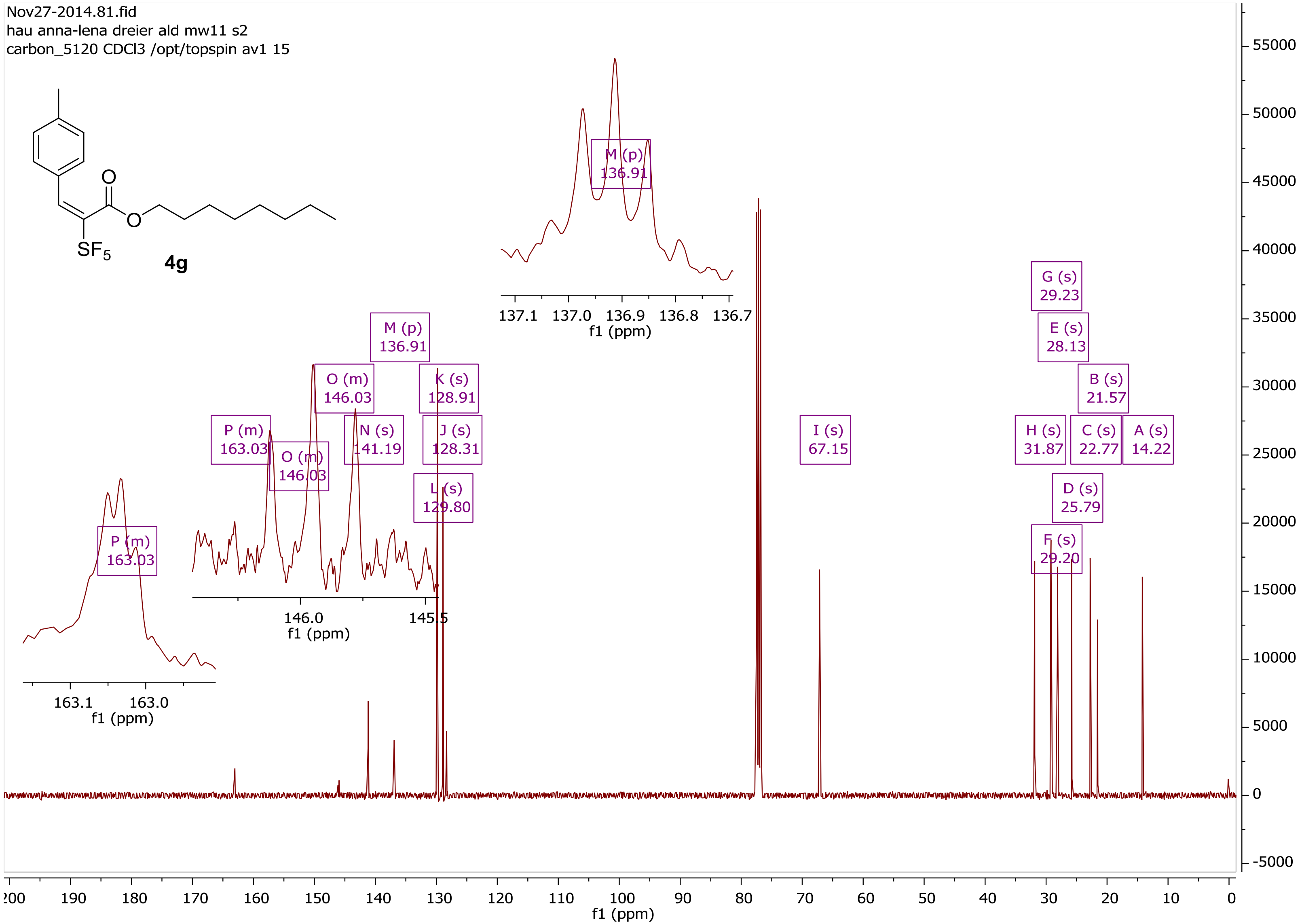

^{19}F NMR spectrum of compound **4g**

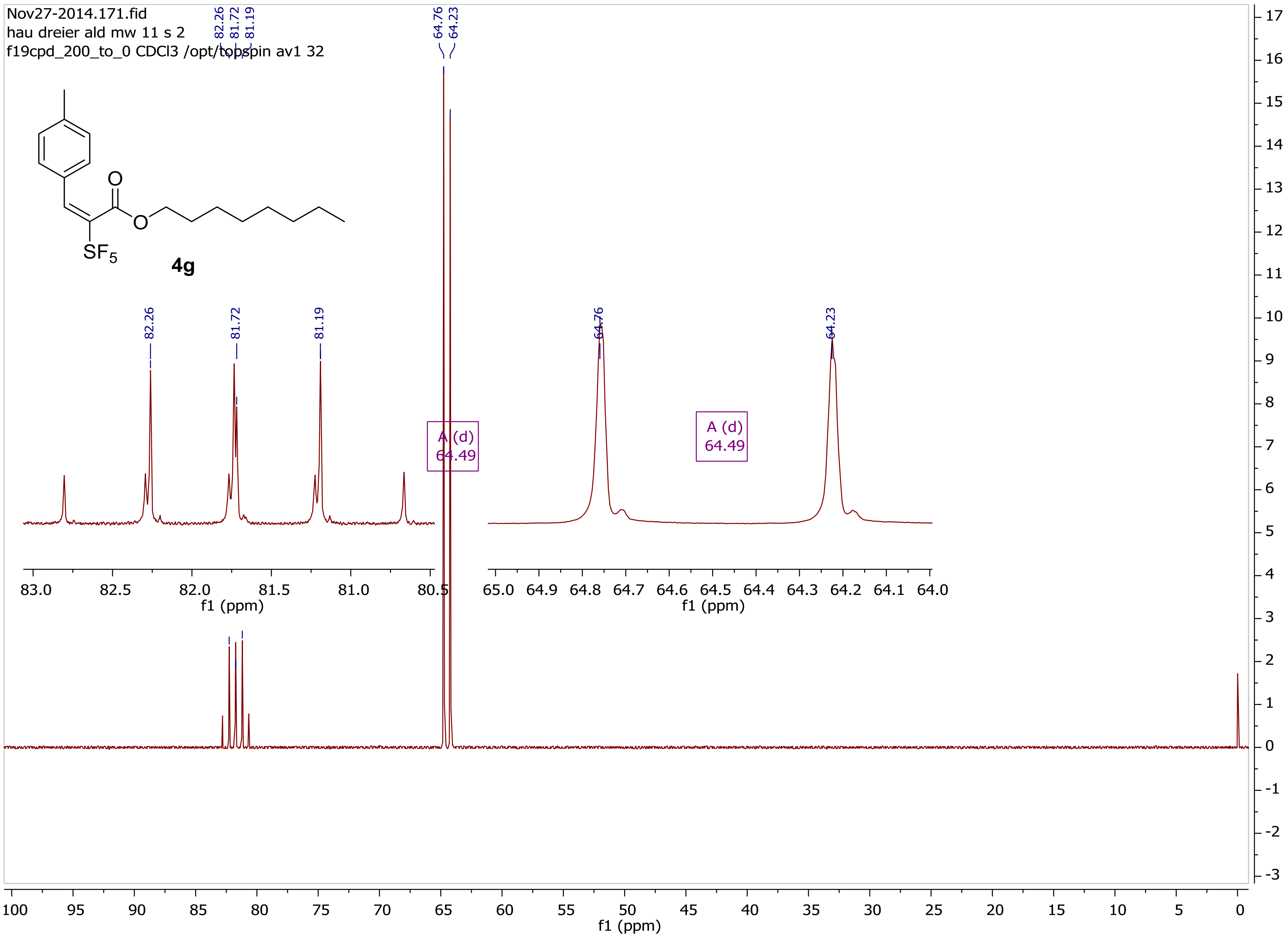

^{1}H NMR spectrum of compound **4h**

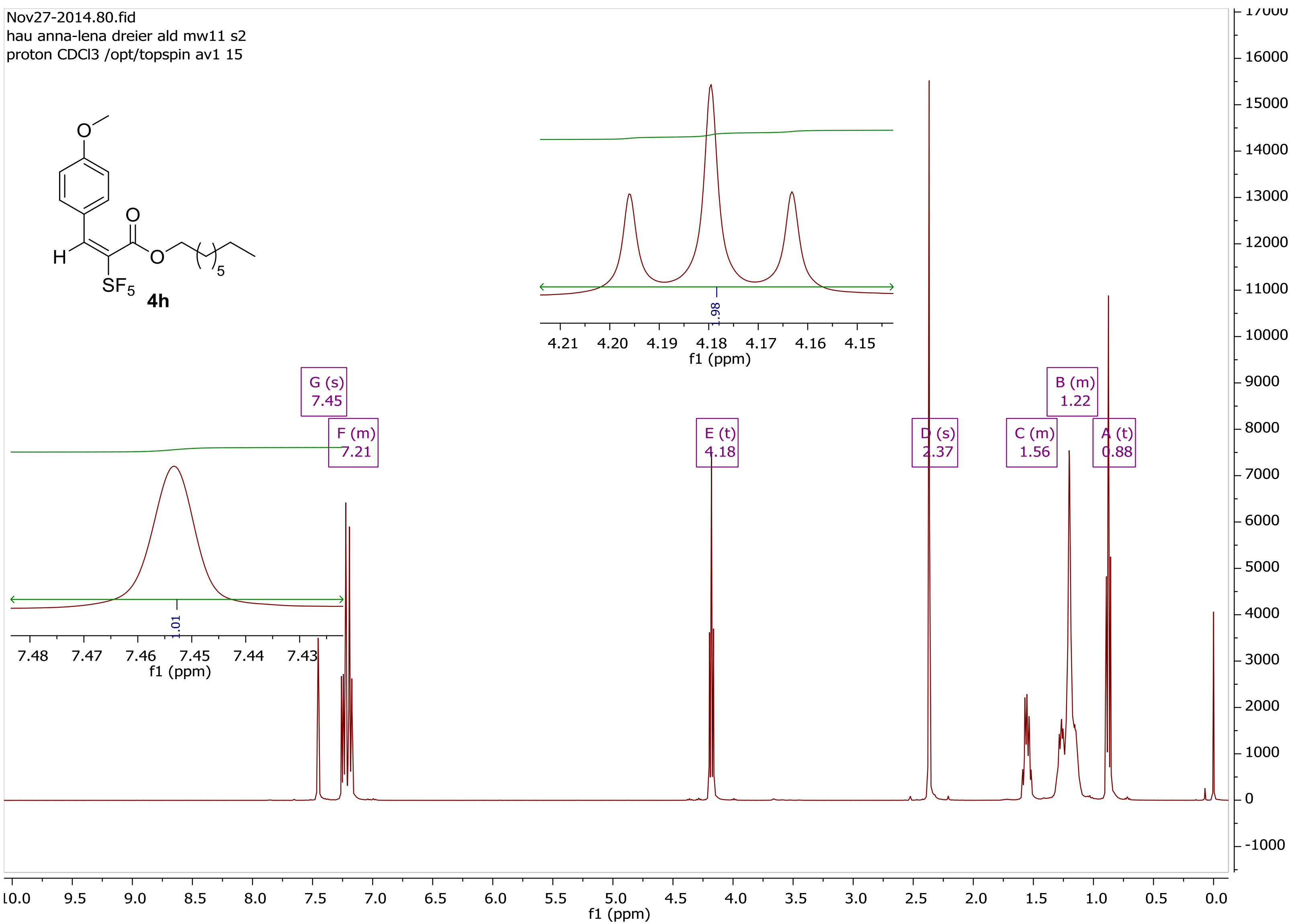

^{13}C NMR spectrum of compound **4h**

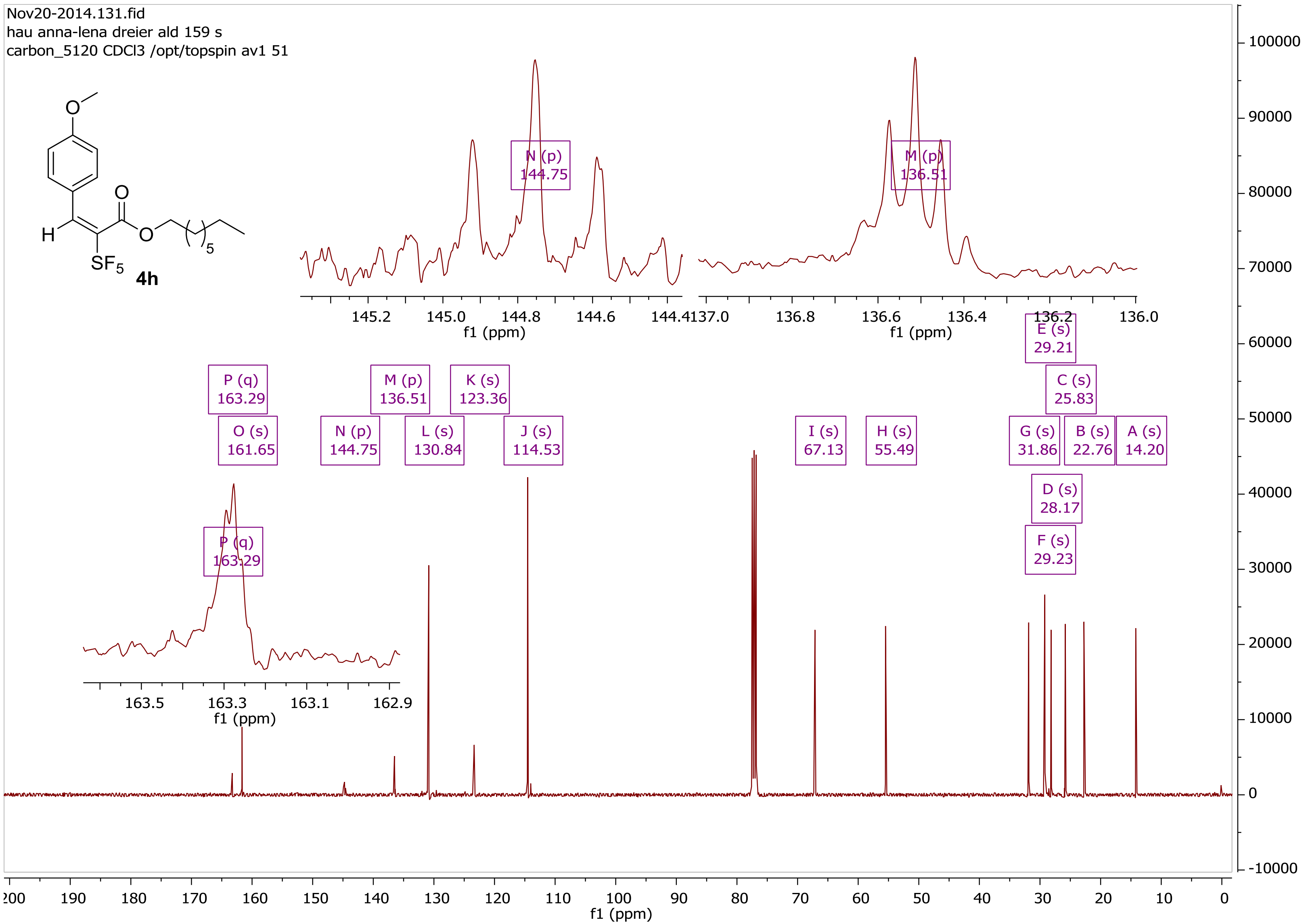

^{19}F NMR spectrum of compound **4h**

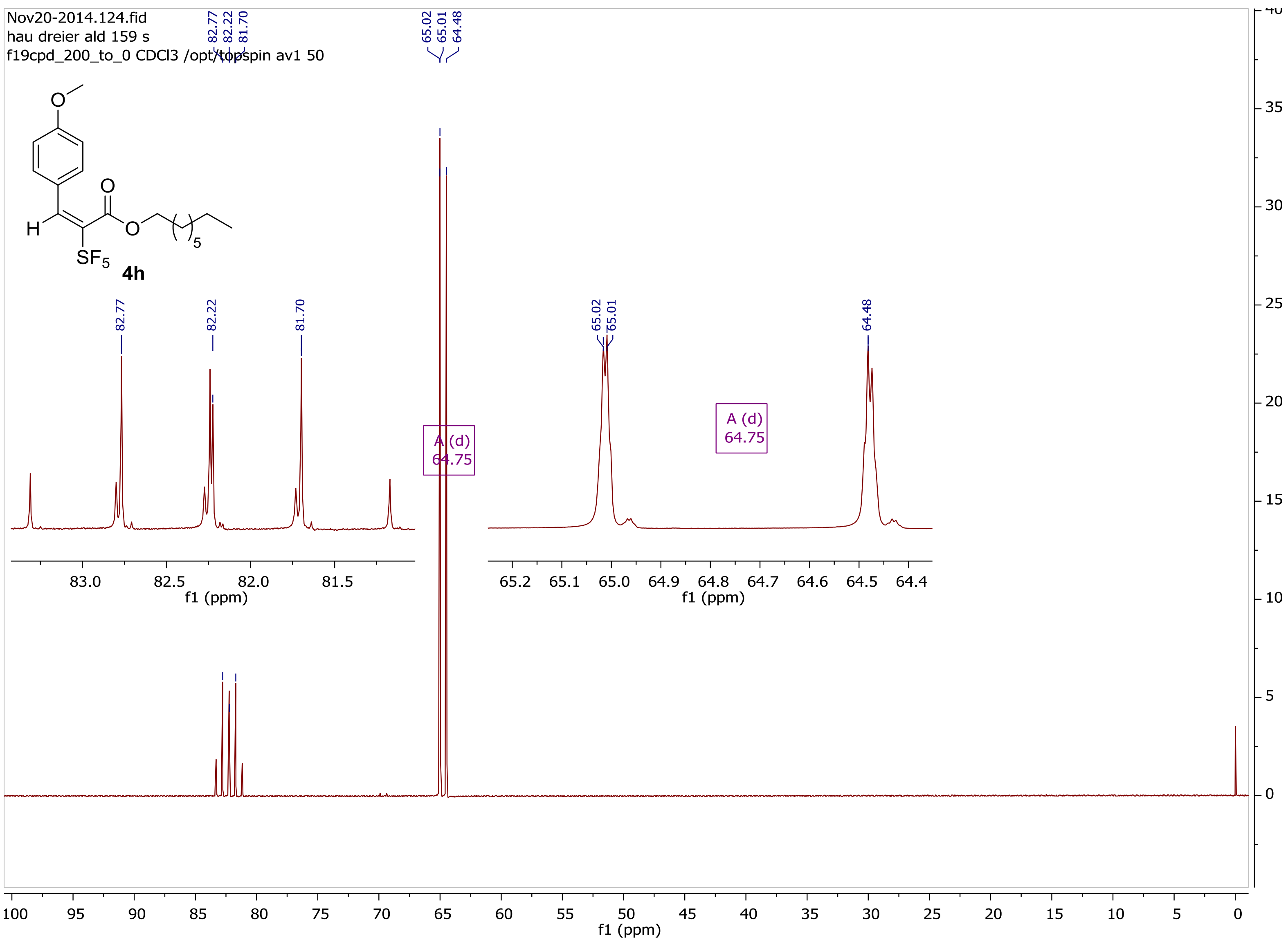

^{1}H,^{19}F-correlation spectrum (gHOESY) of **4h**

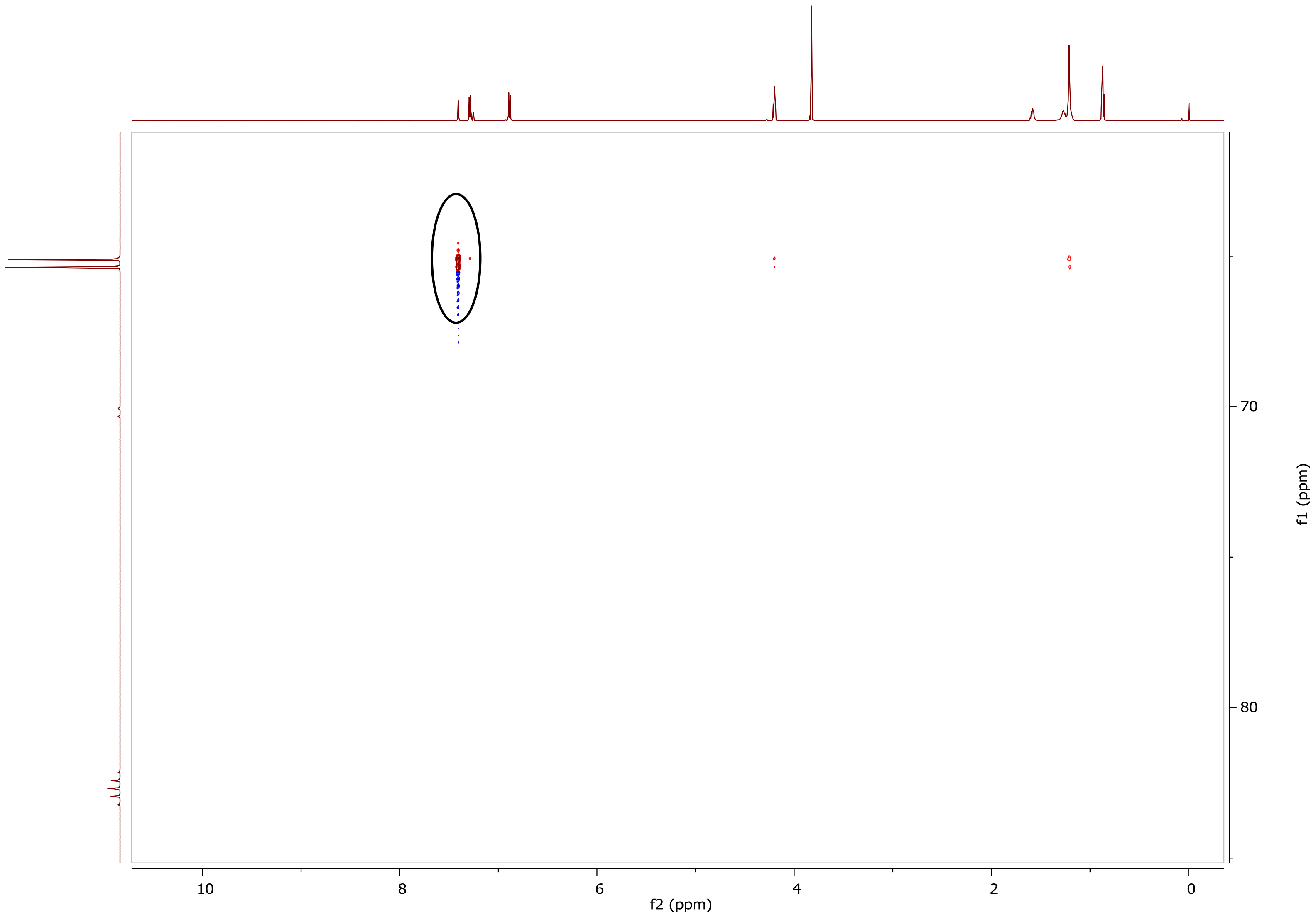

^{1}H NMR spectrum of compound **4i**

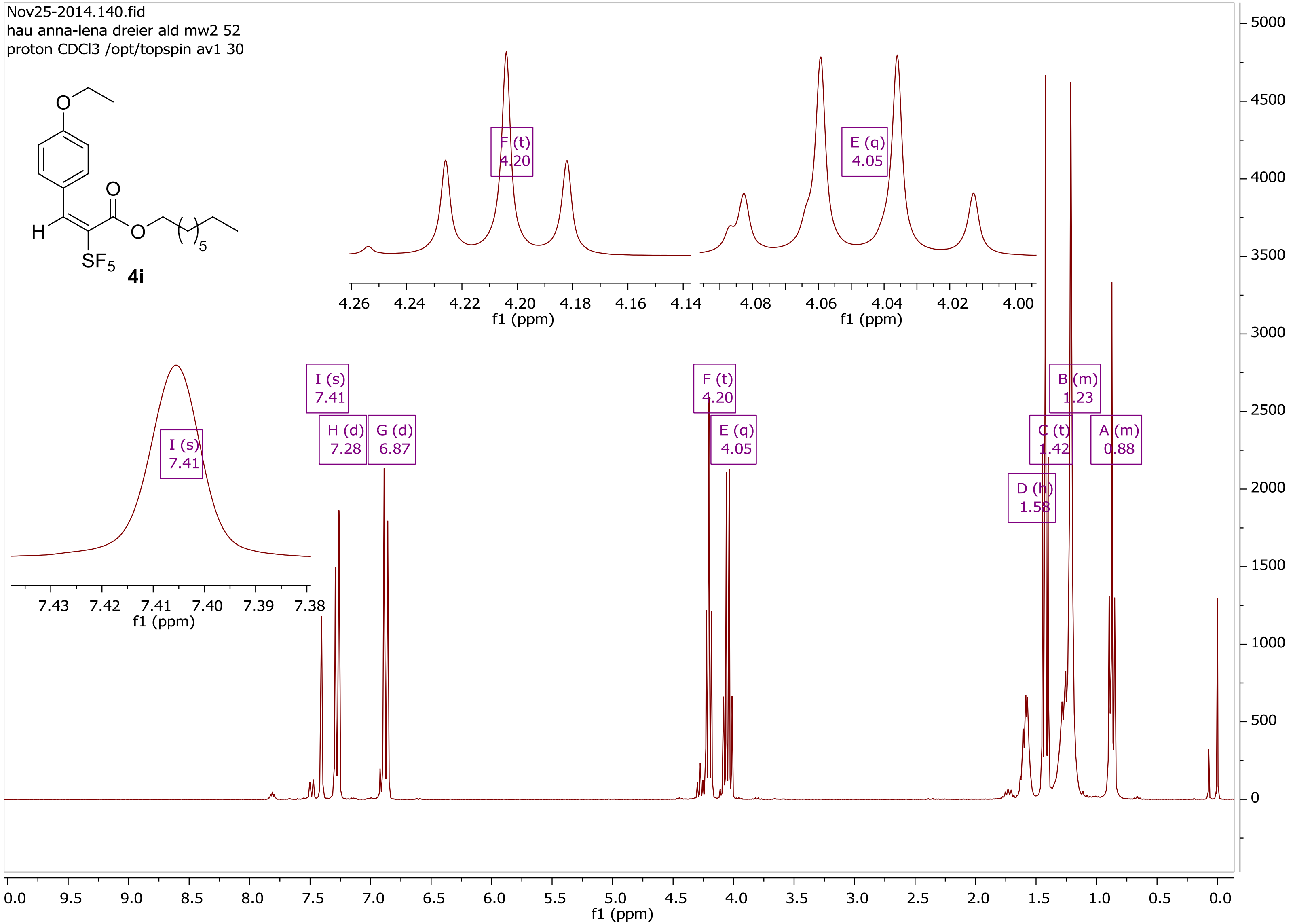

^{13}C NMR spectrum of compound **4i**

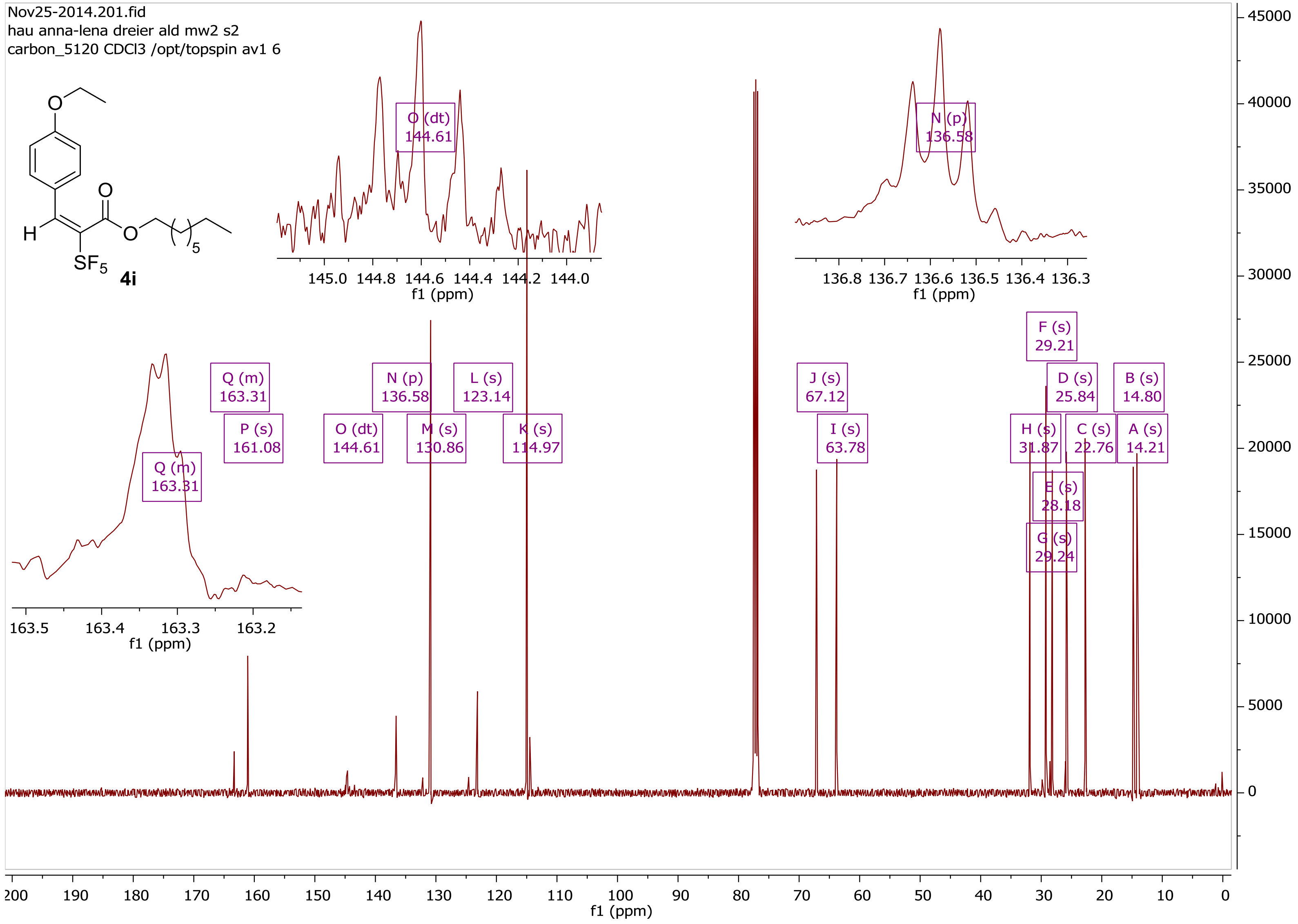

^{19}F NMR spectrum of compound **4i**

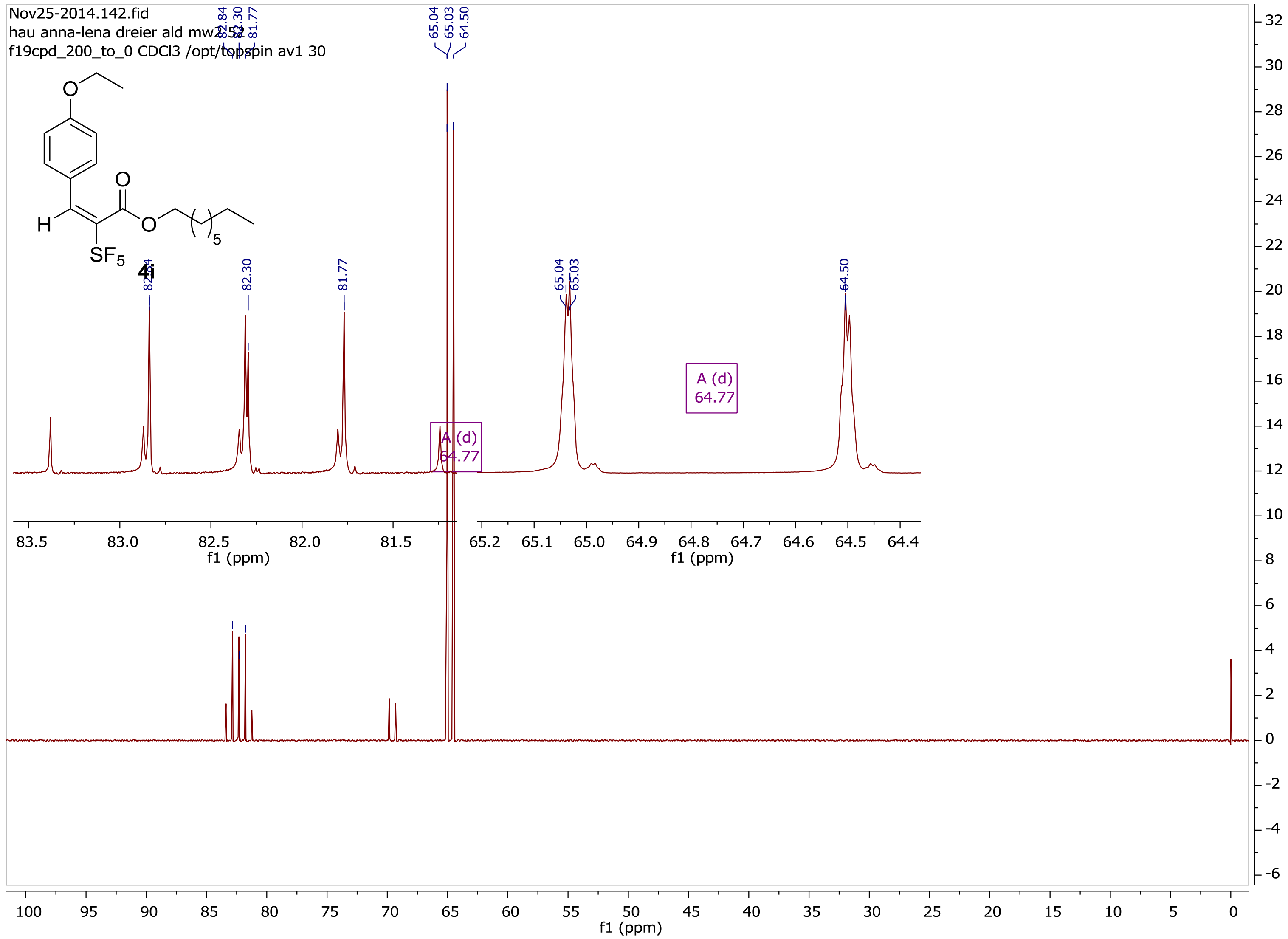

^{1}H NMR spectrum of compound **2j**

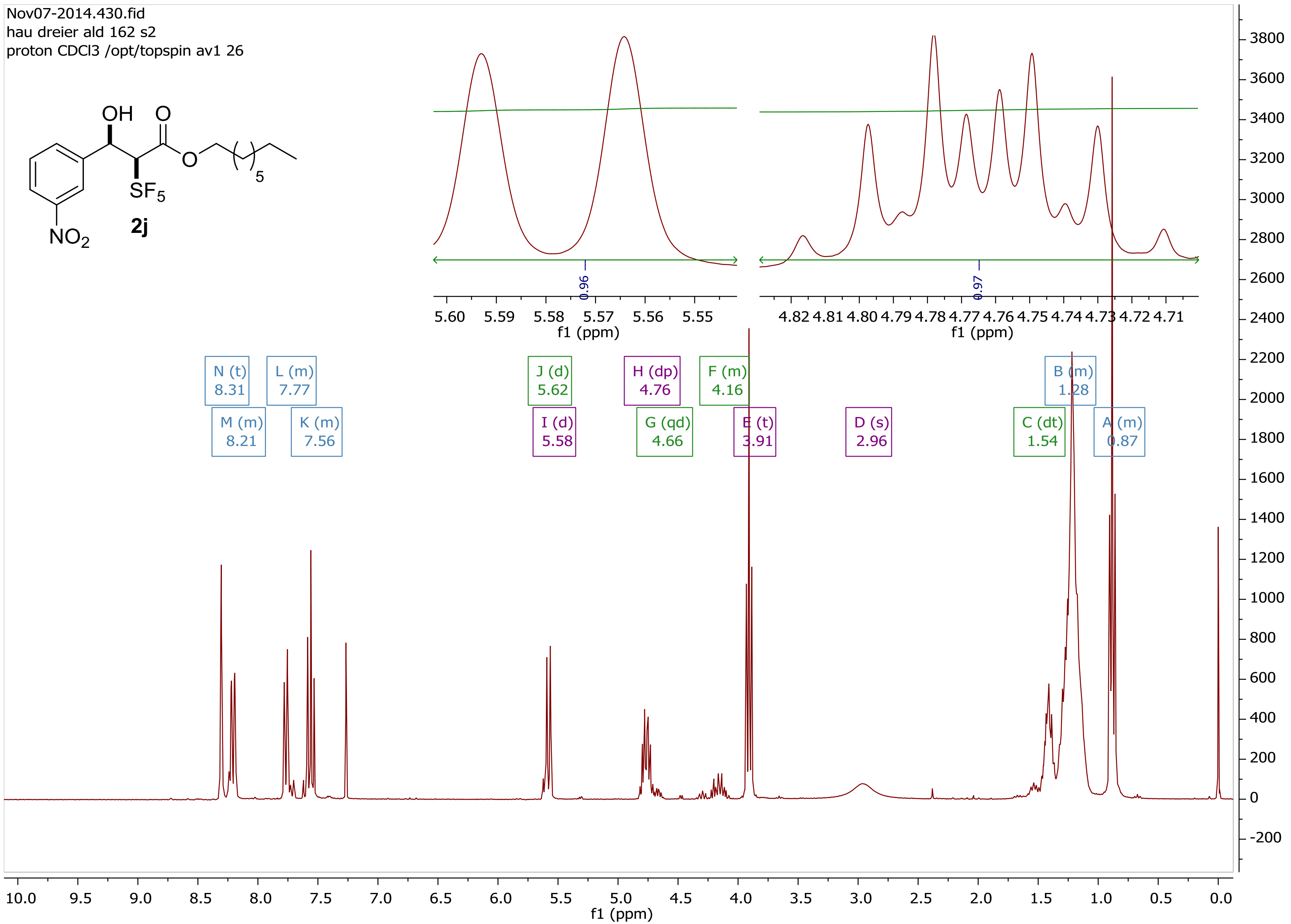

^{13}C NMR spectrum of compound **2j**

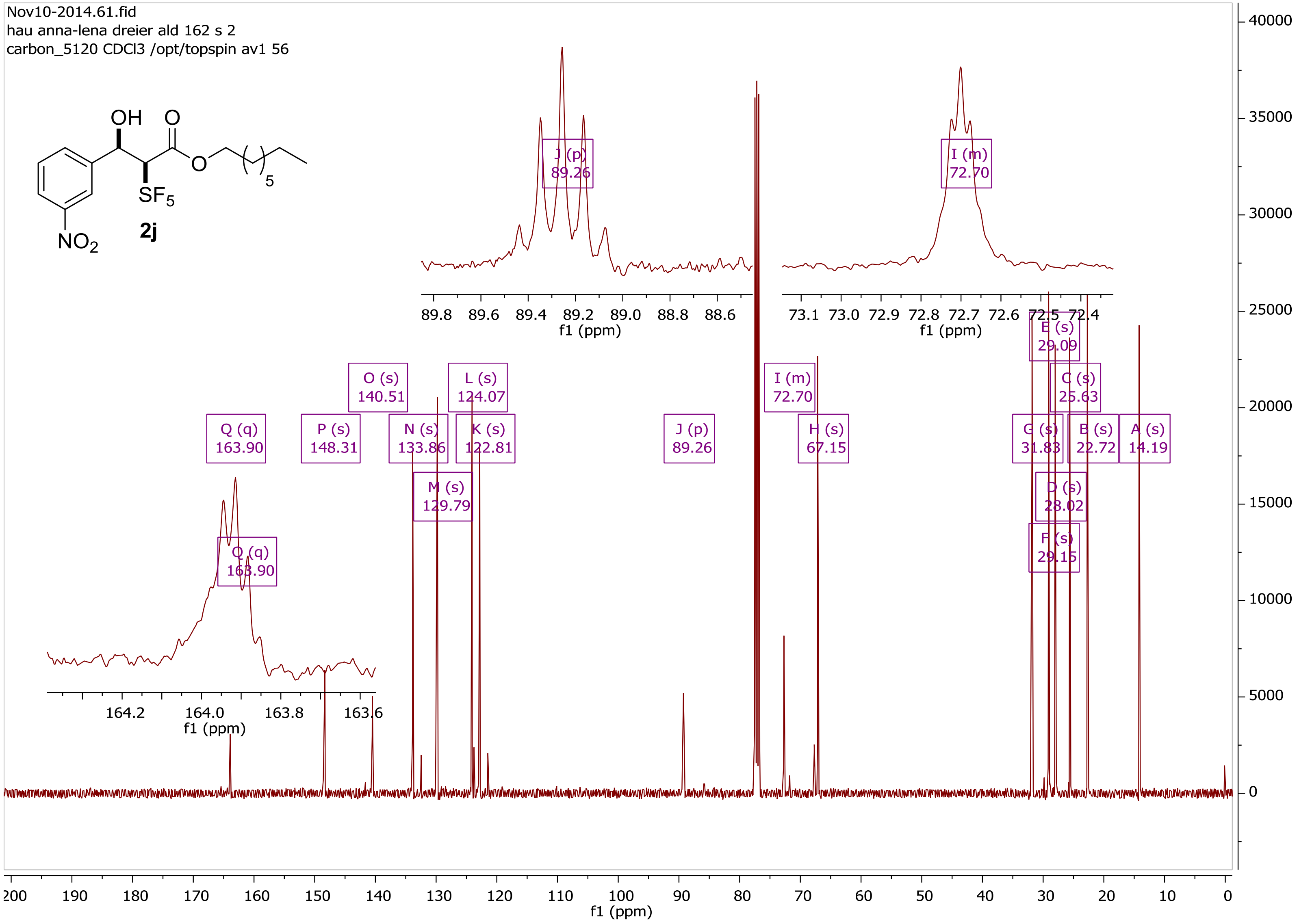

^{19}F NMR spectrum of compound **2j**

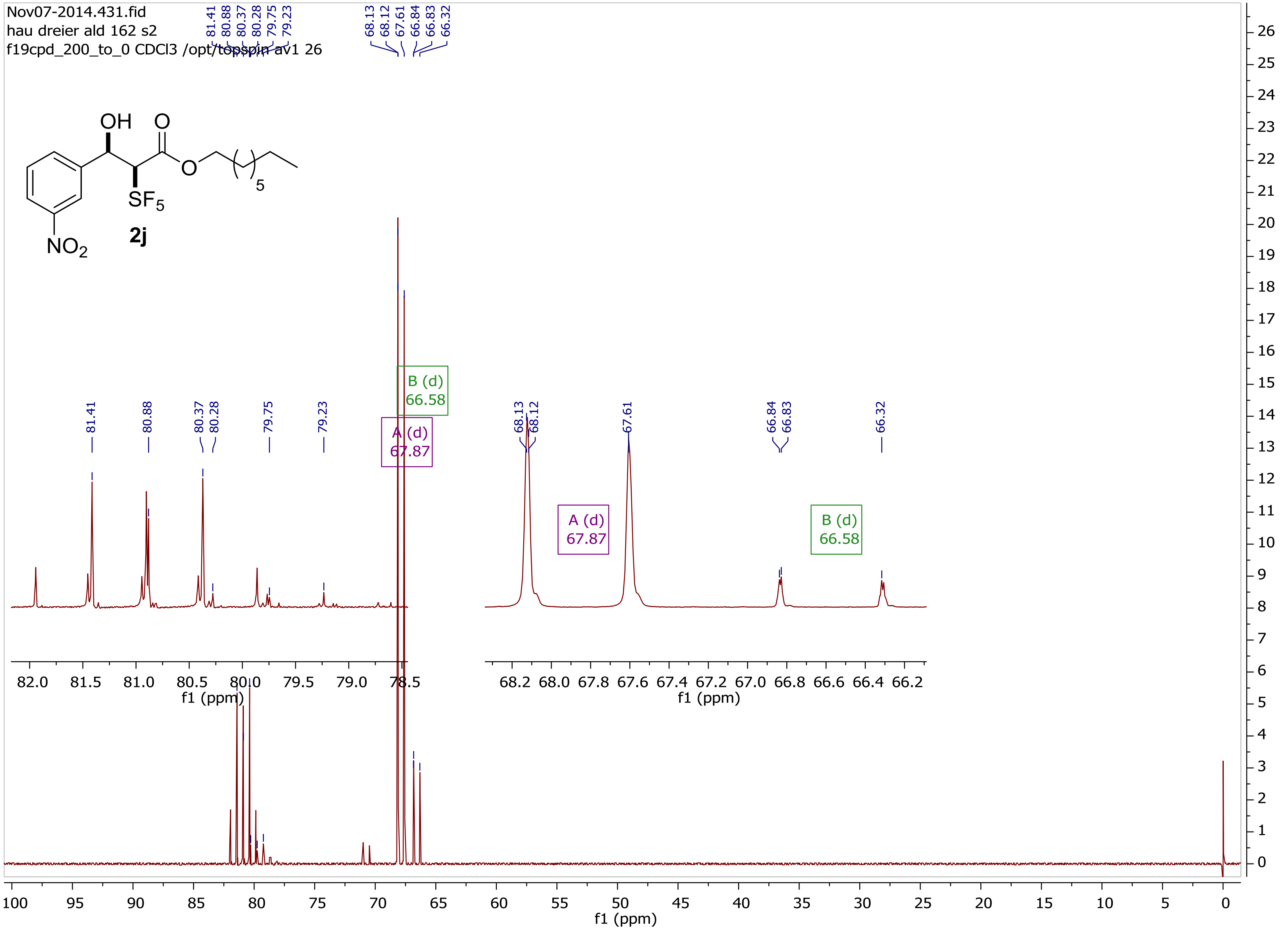

^{1}H NMR spectrum of compound **2k**

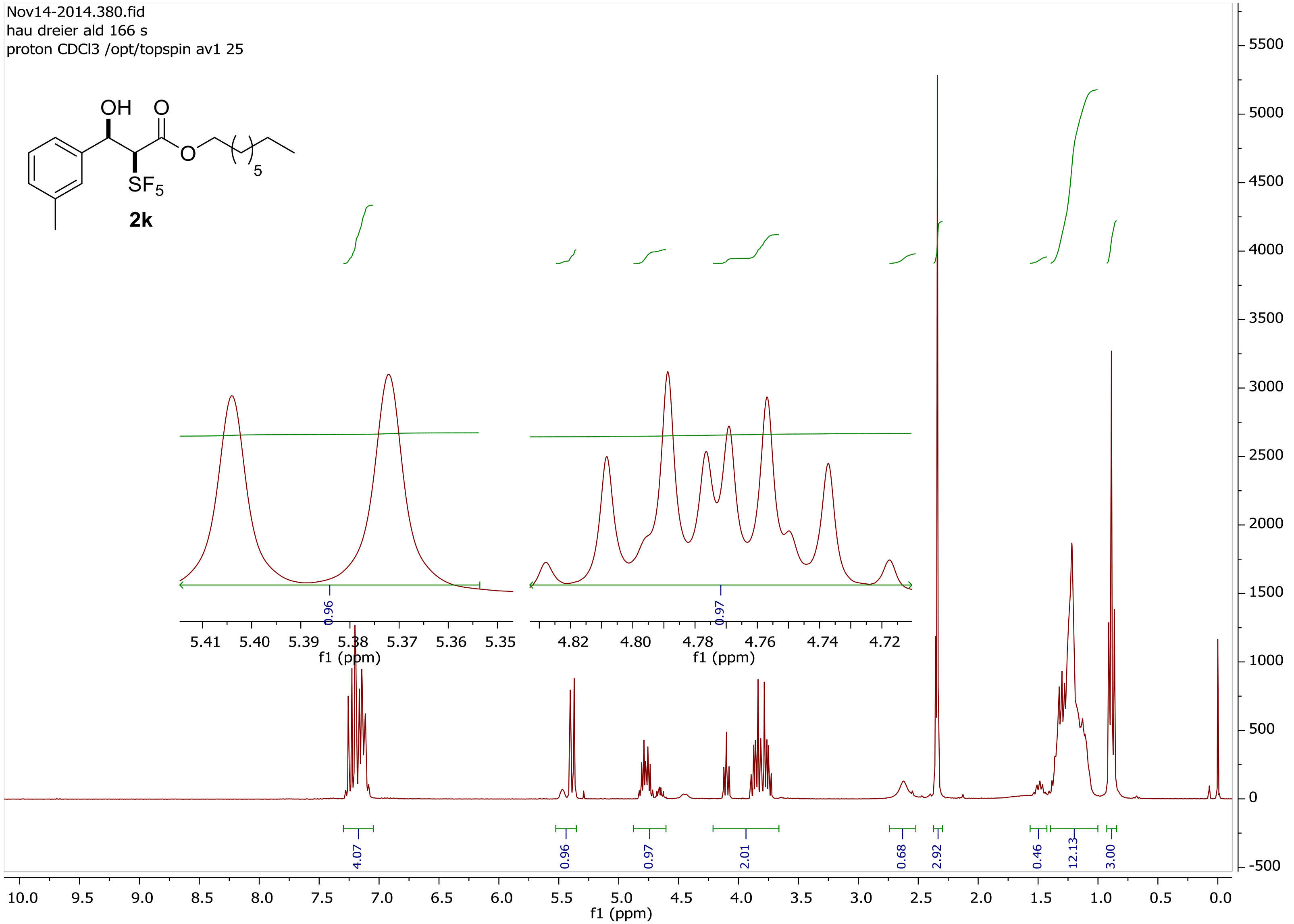

^{13}C NMR spectrum of compound **2k**

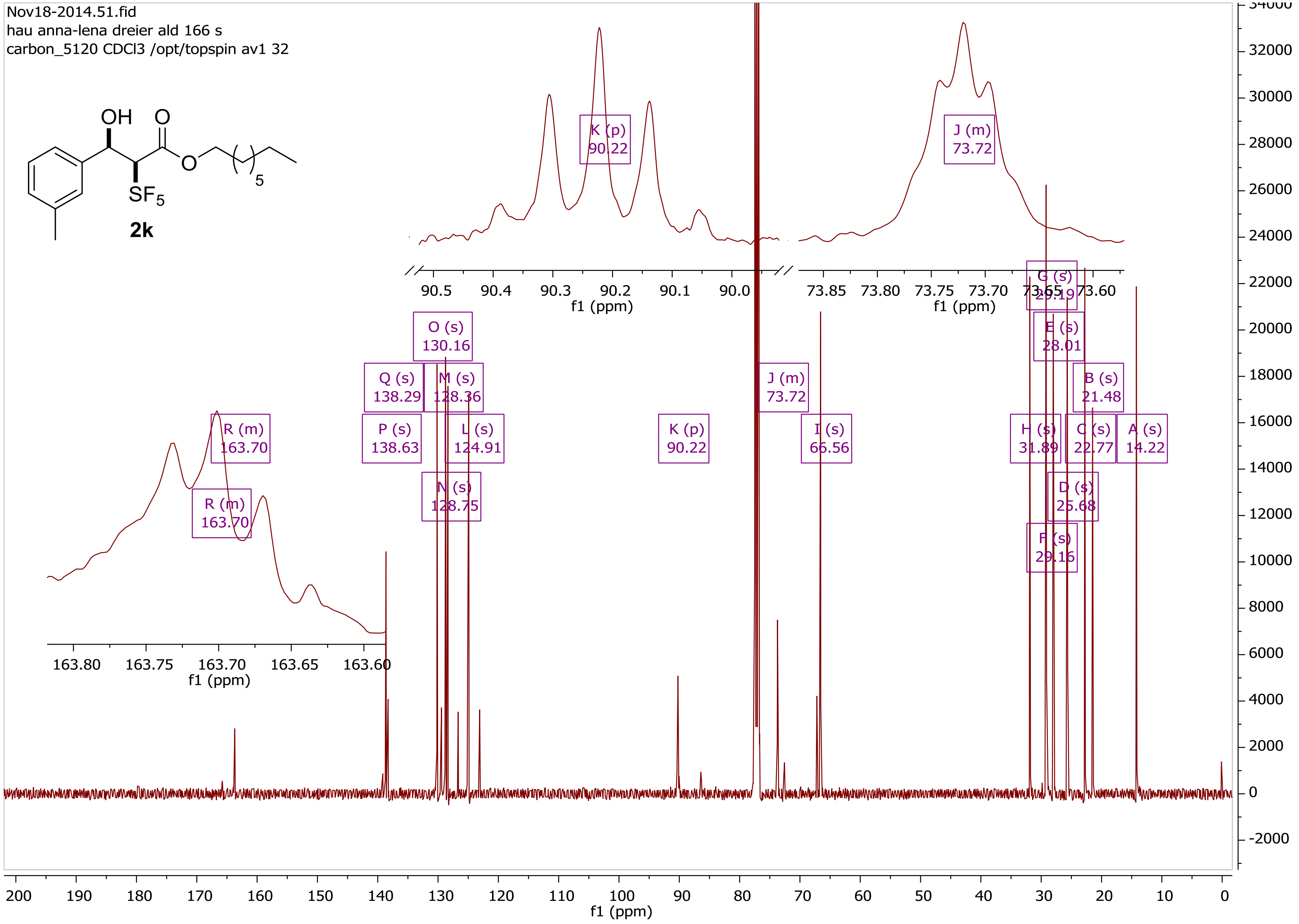

^{19}F NMR spectrum of compound **2k**

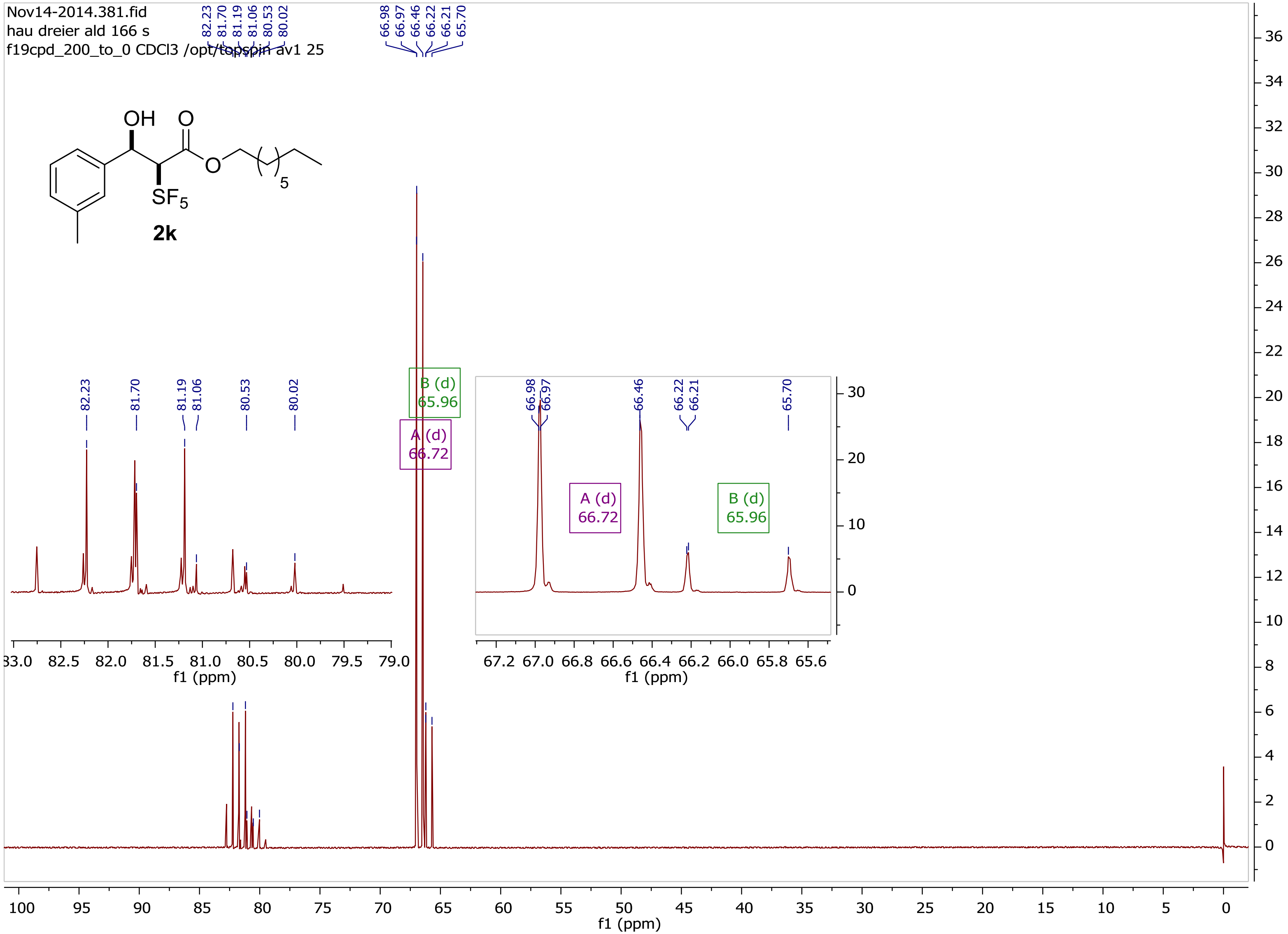

^{1}H NMR spectrum of compound **2l**

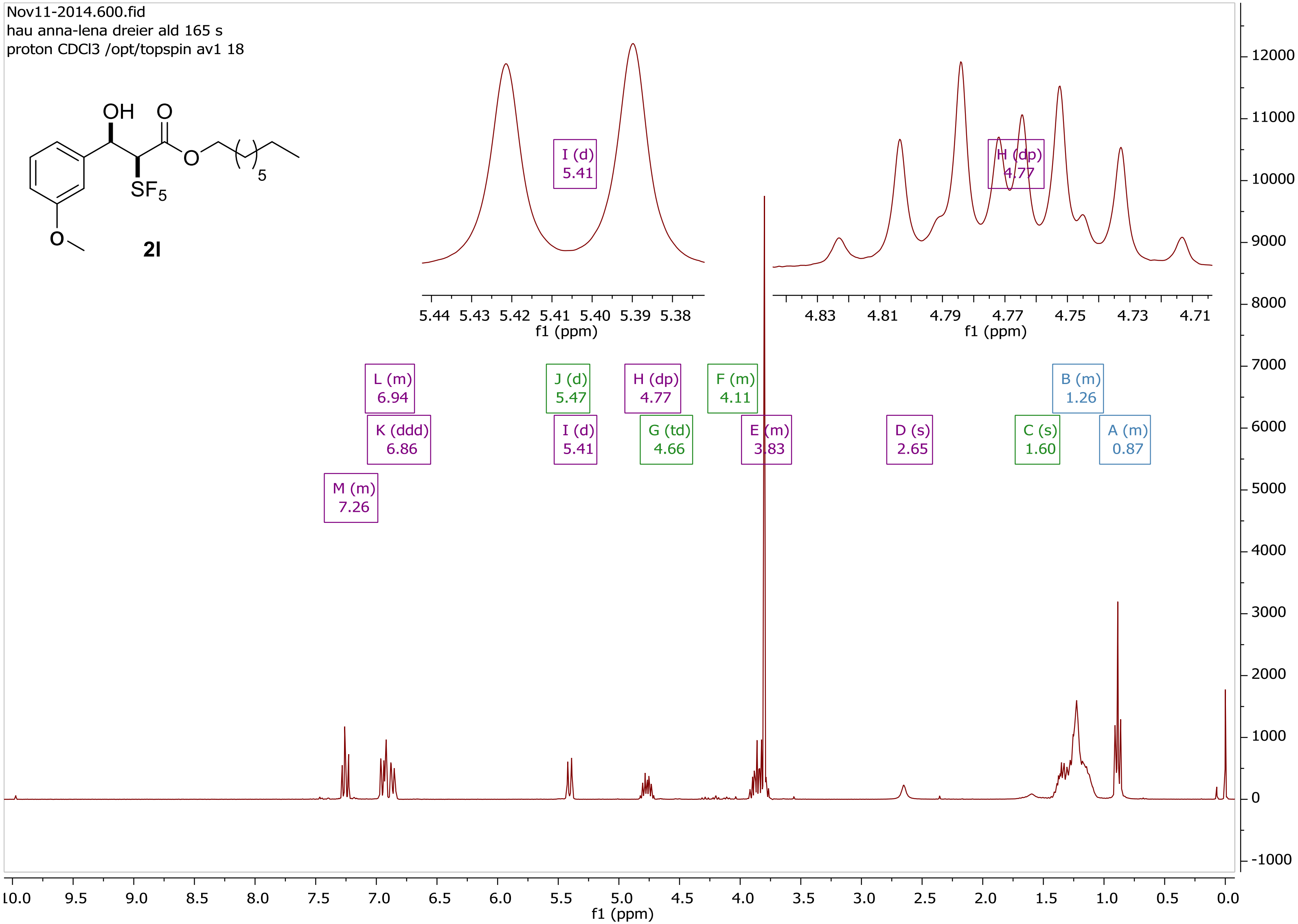

^{13}C NMR spectrum of compound **2l**

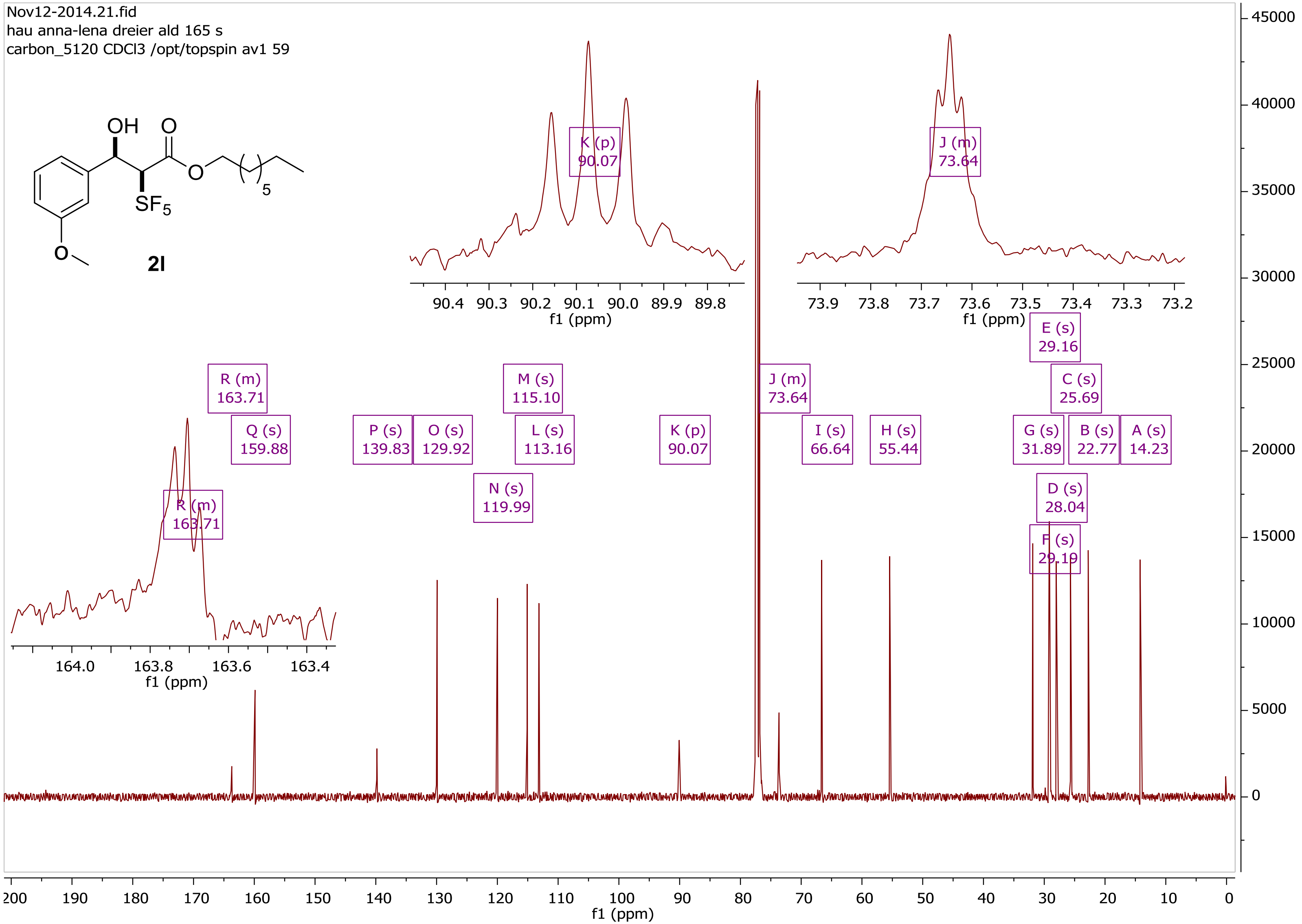

^{19}F NMR spectrum of compound **2l**

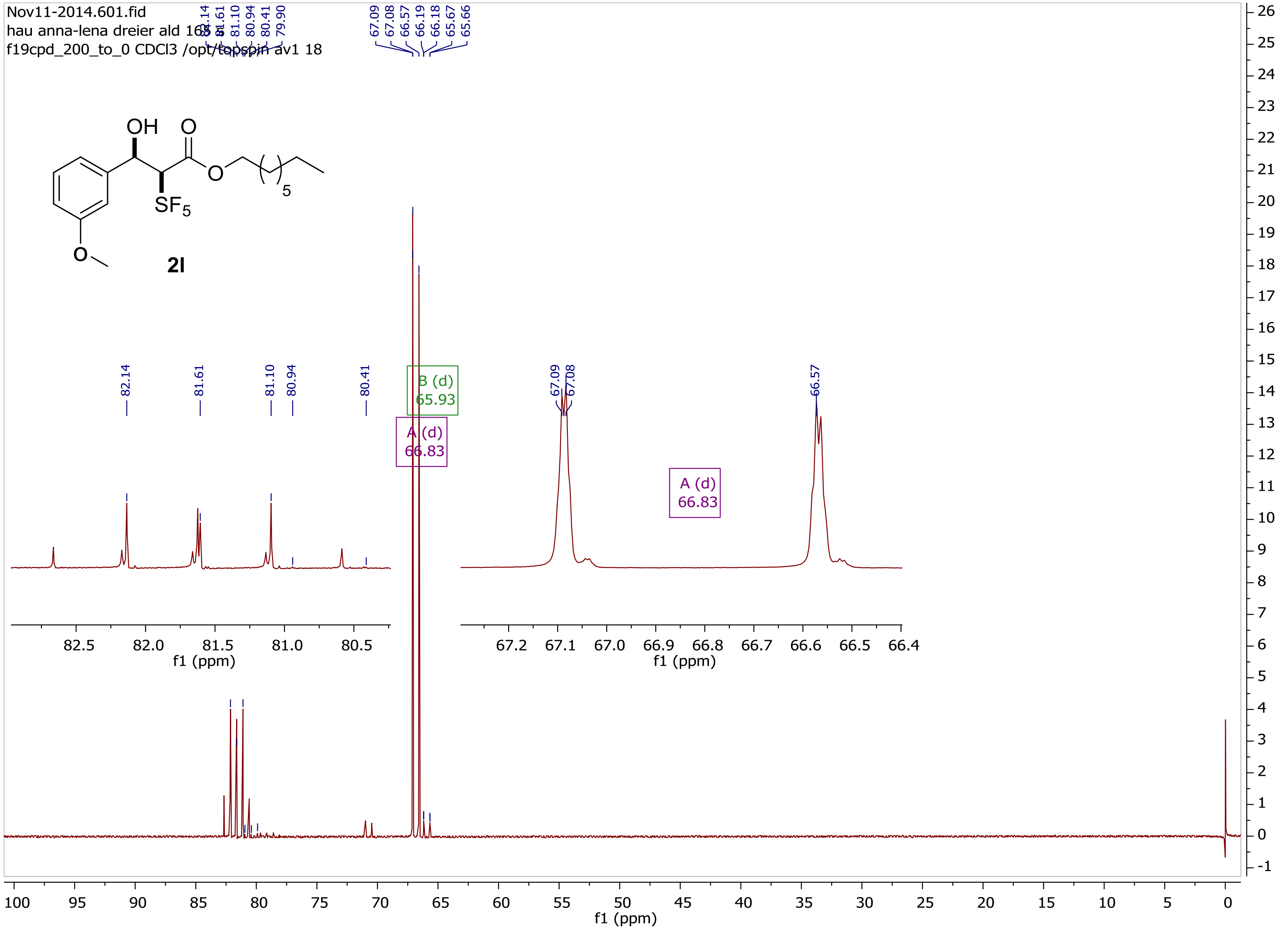

^{1}H NMR spectrum of compound **2m**

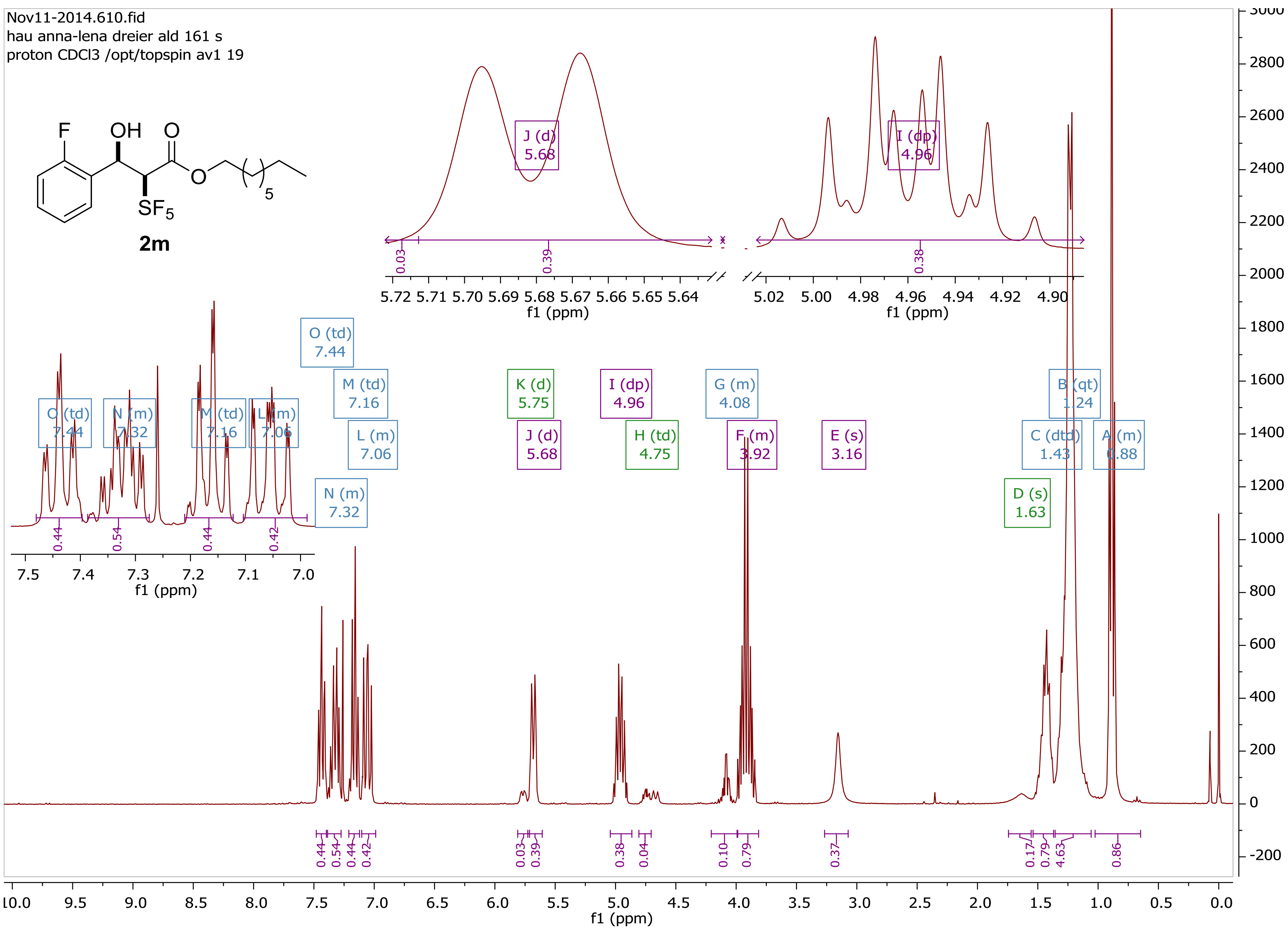

^{13}C NMR spectrum of compound **2m**

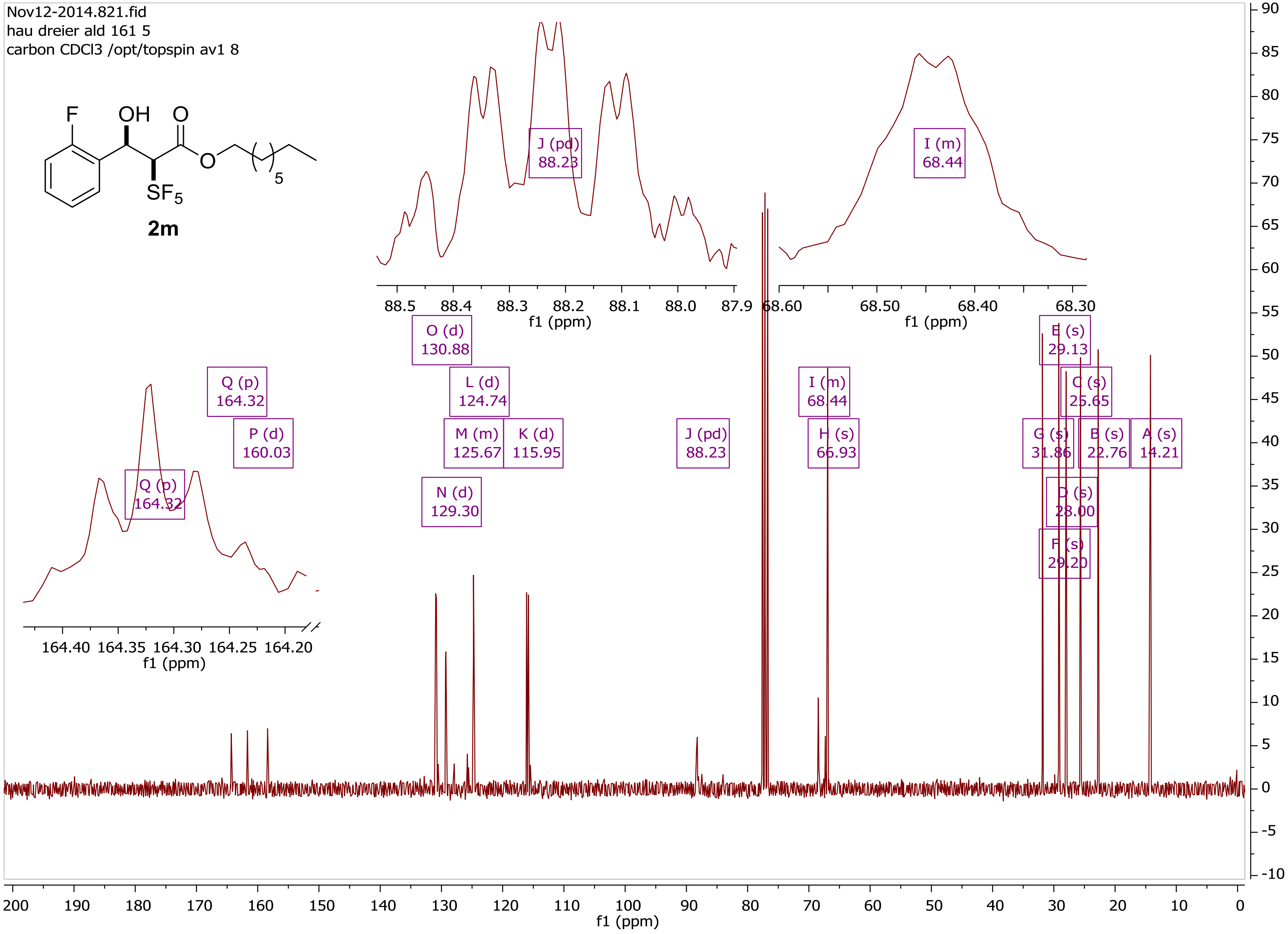

^{19}F NMR spectrum of compound **2m**

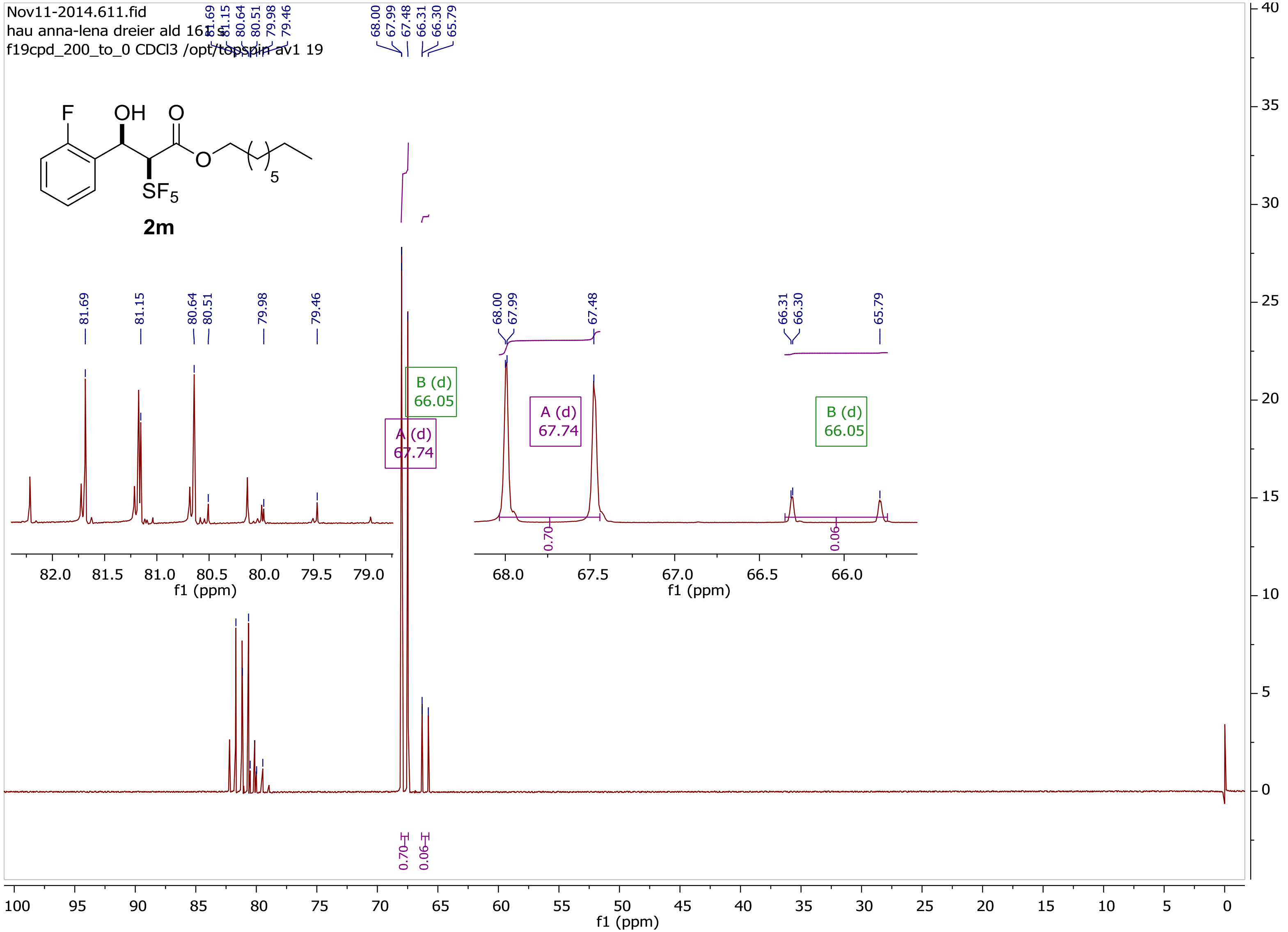

^{1}H NMR spectrum of compound **2q**

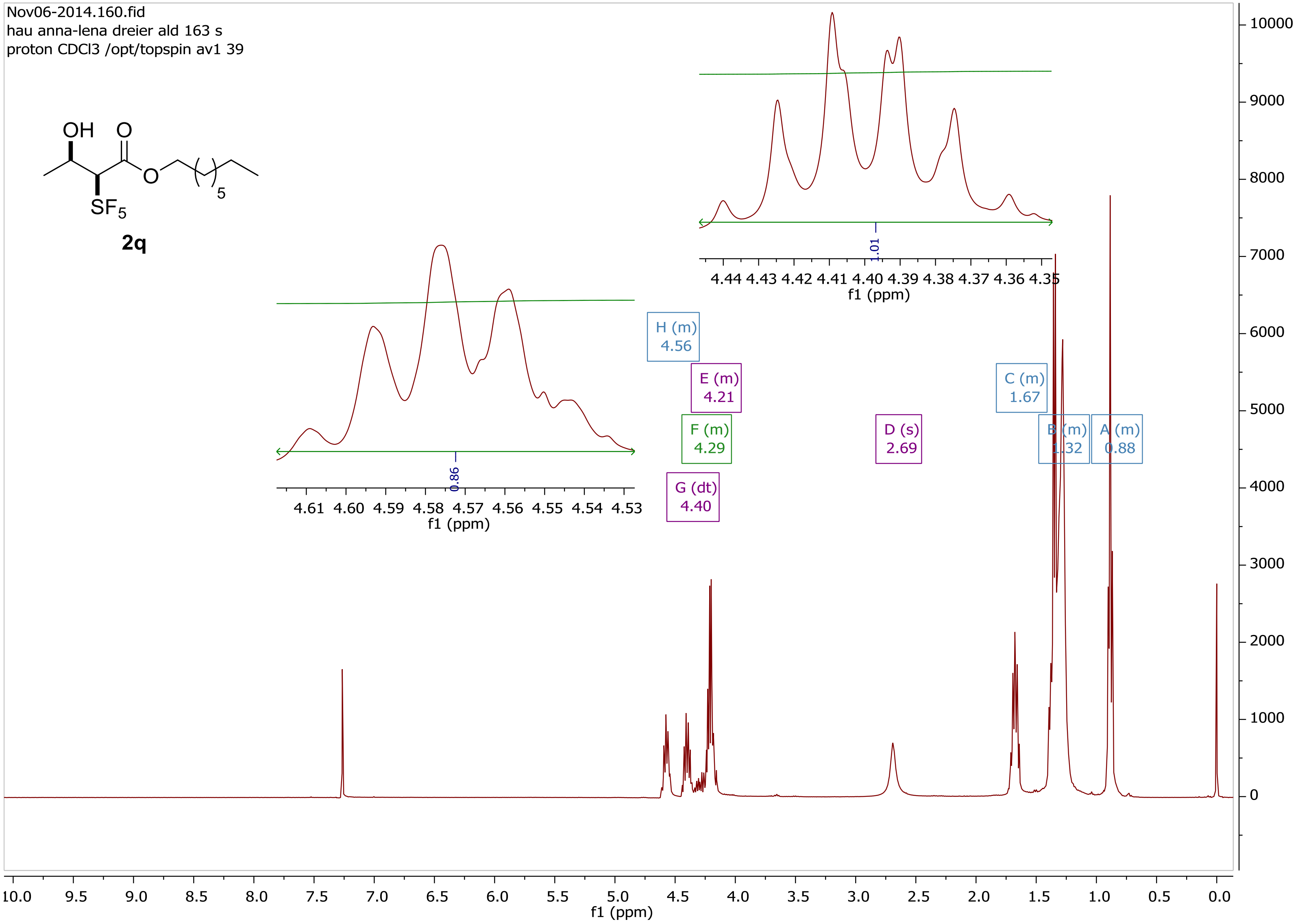

^{13}C NMR spectrum of compound **2q**

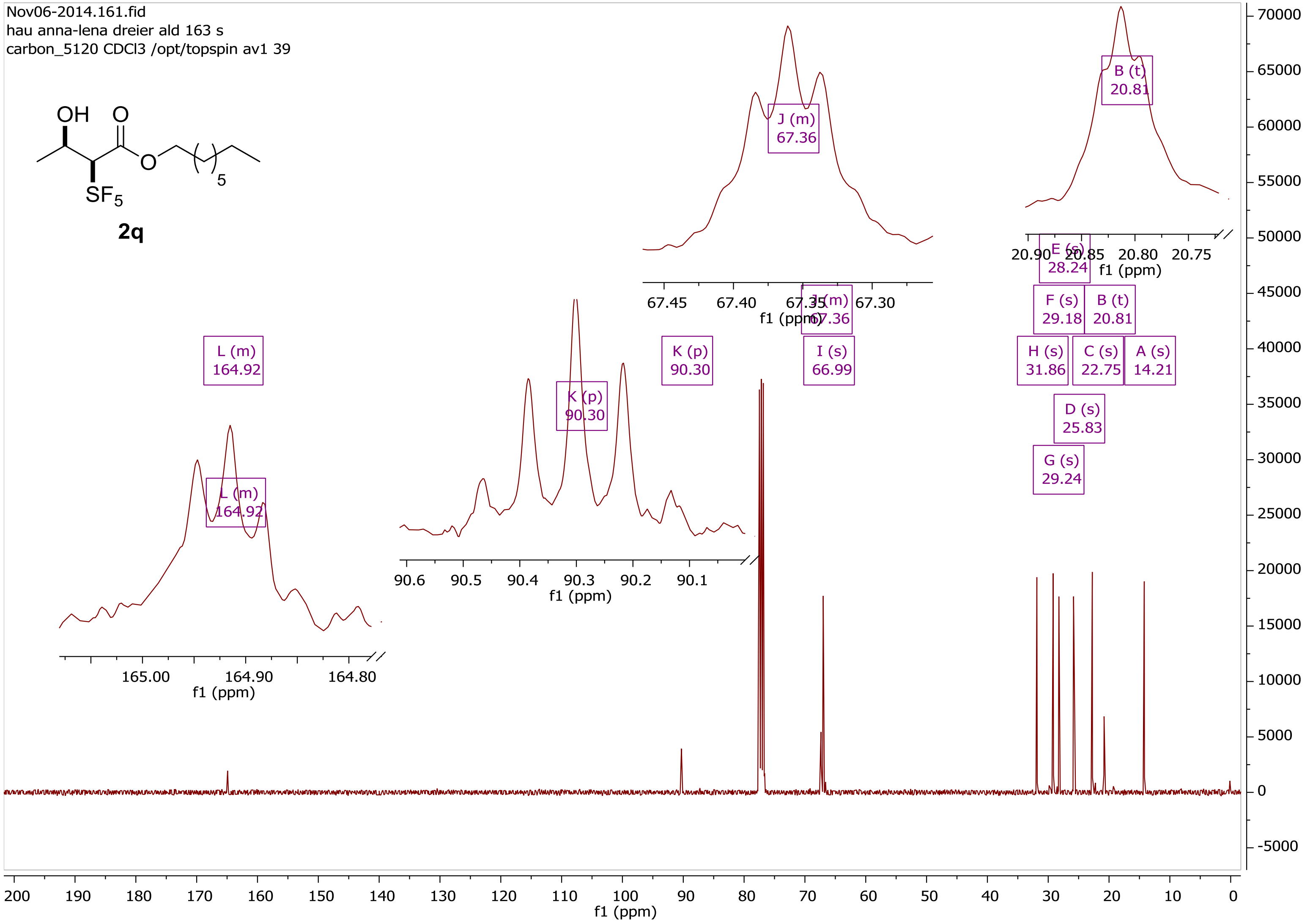

^{19}F NMR spectrum of compound **2q**

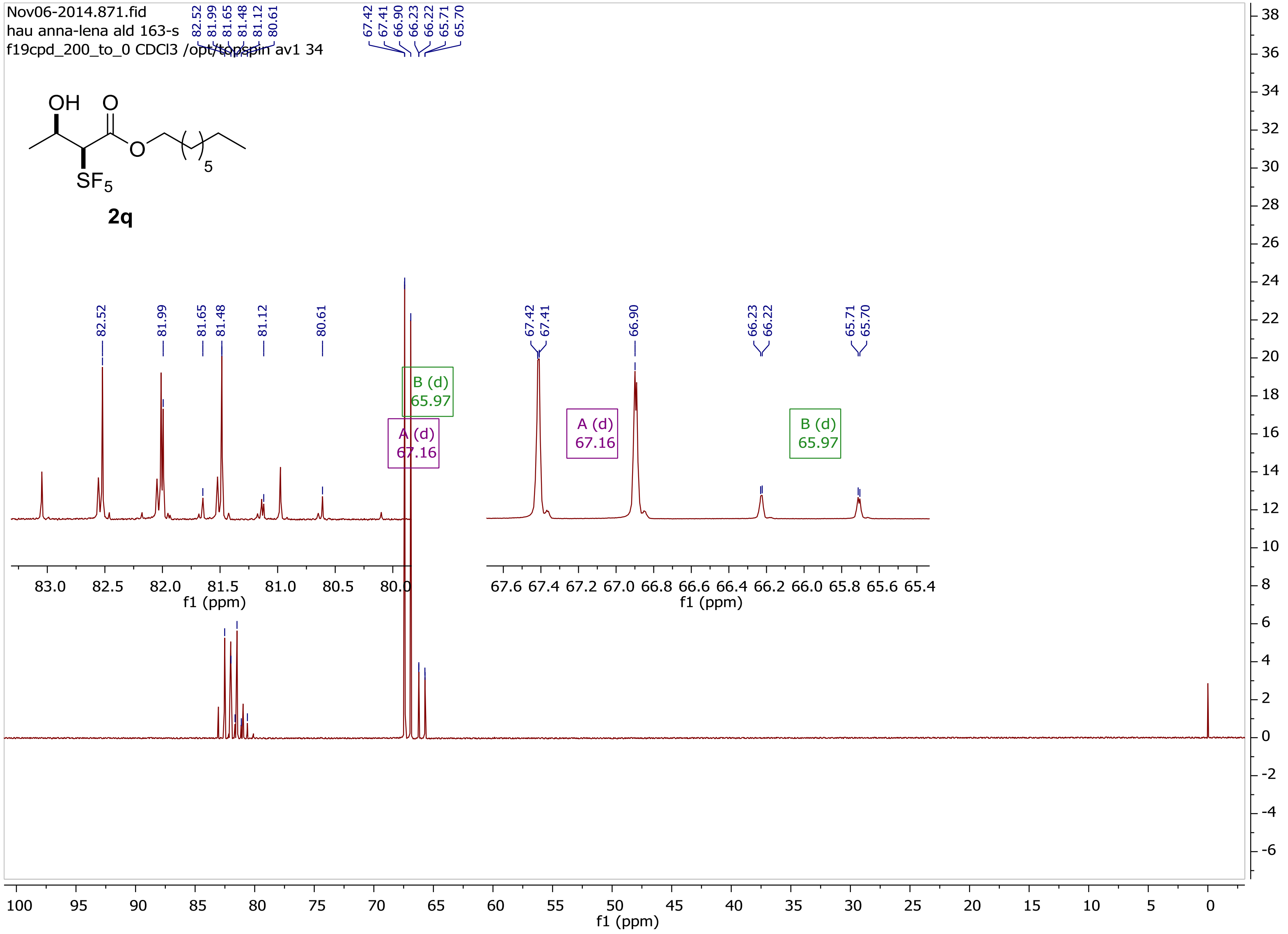

^{1}H NMR spectrum of compound **8**

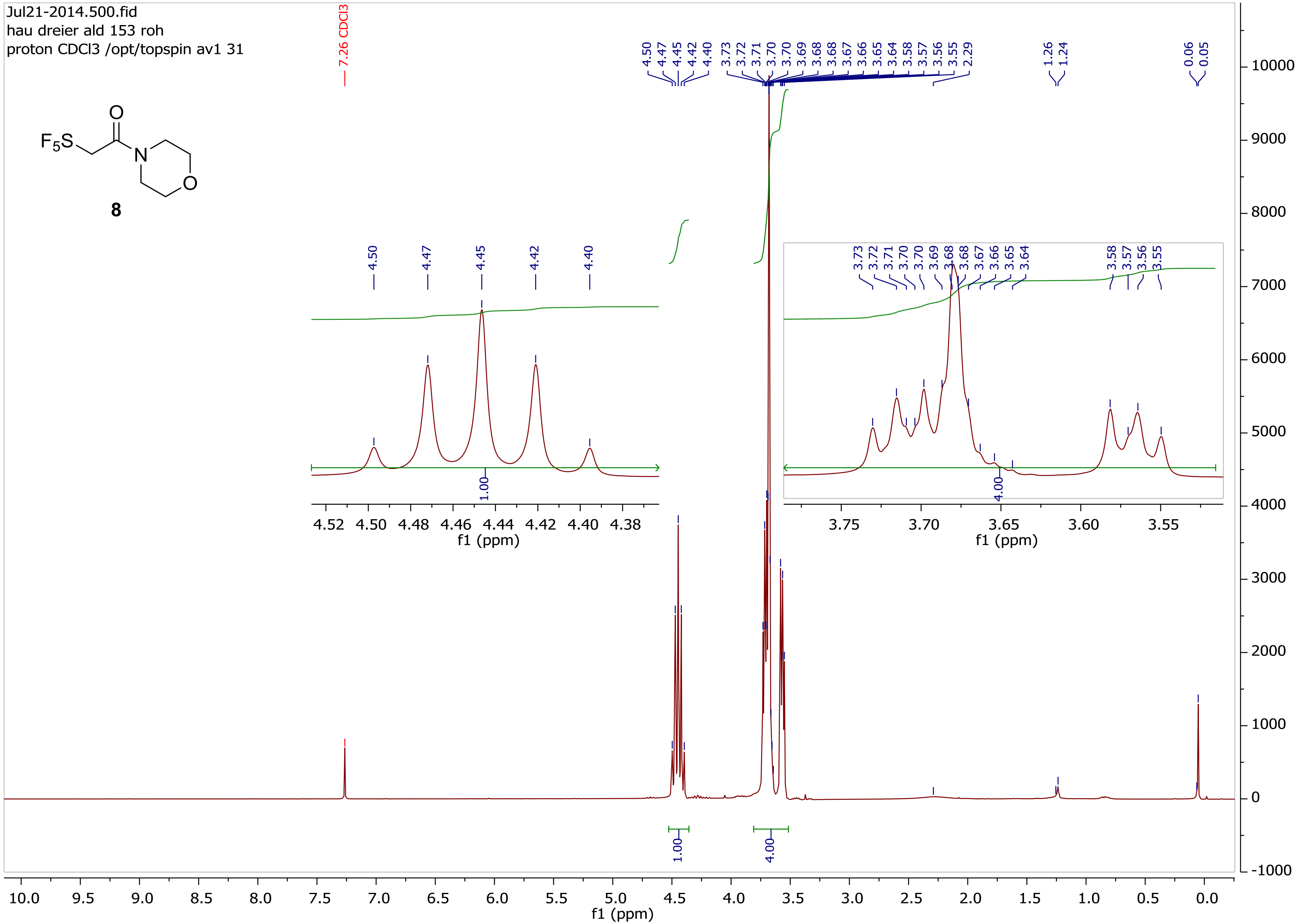

^{13}C NMR spectrum of compound **8**

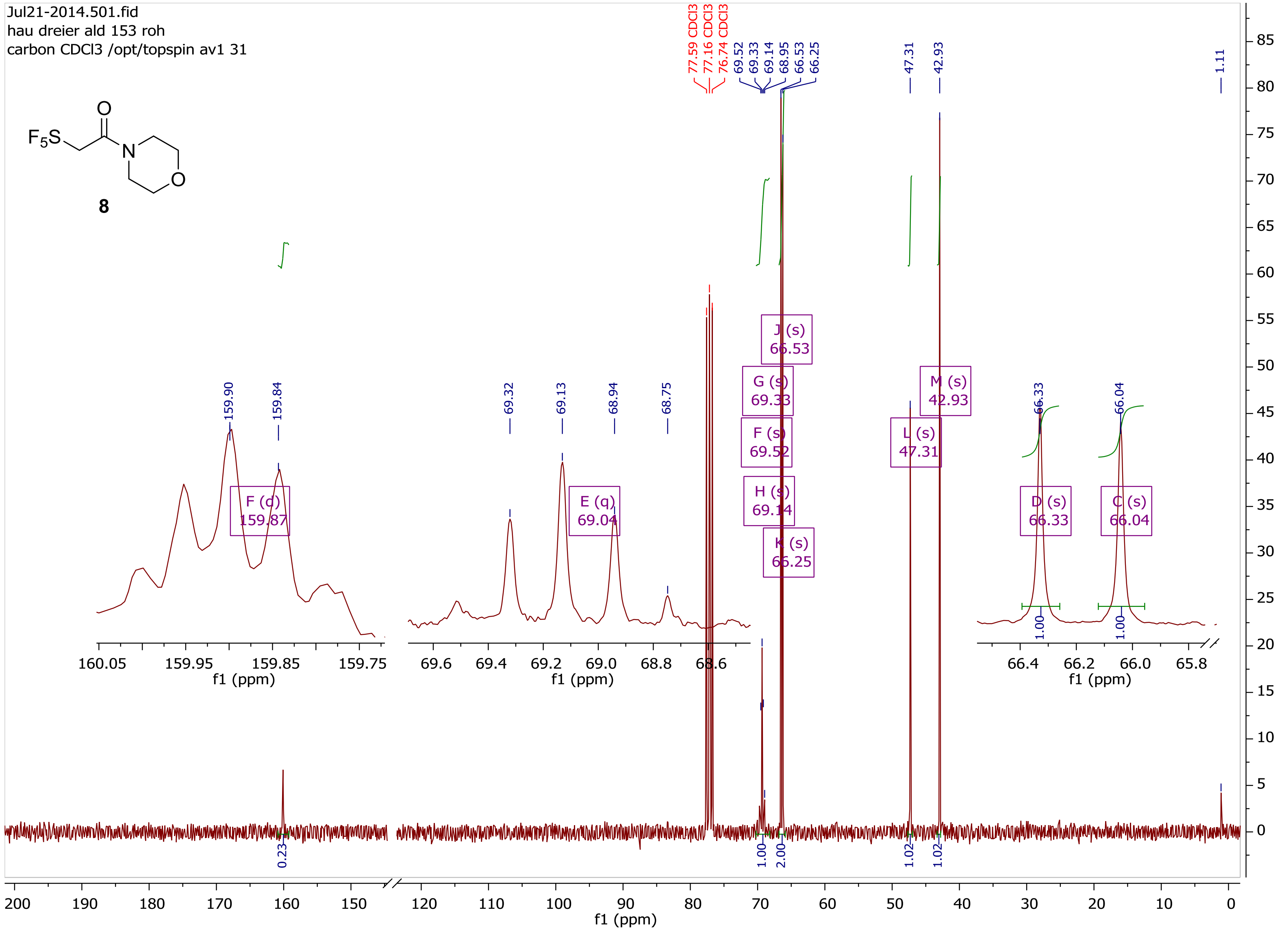

^{19}F NMR spectrum of compound **8**

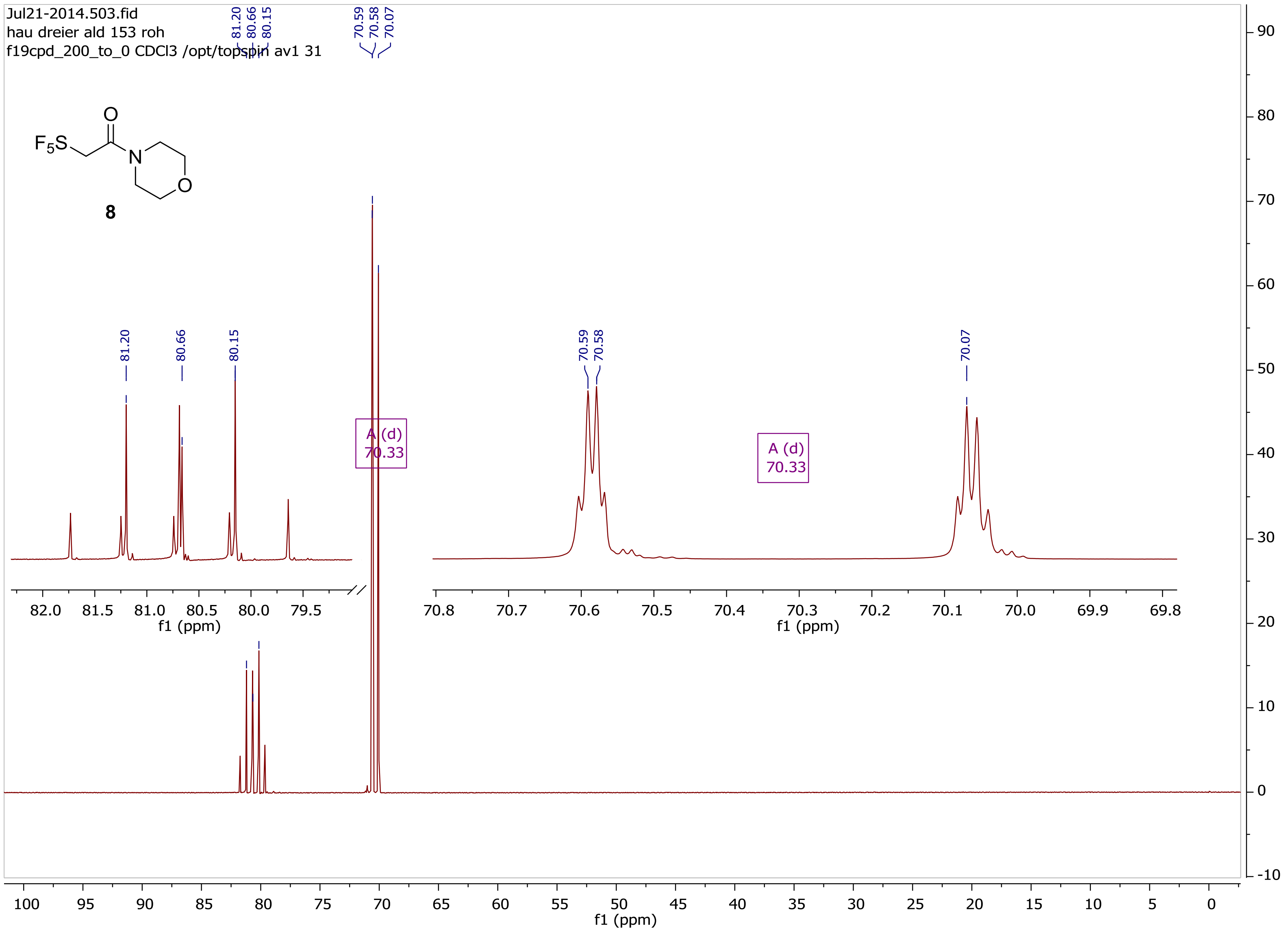

^{1}H NMR spectrum of the (*Z*)-ketene aminal formed from compound **8** in CD_2Cl_2 (crude)

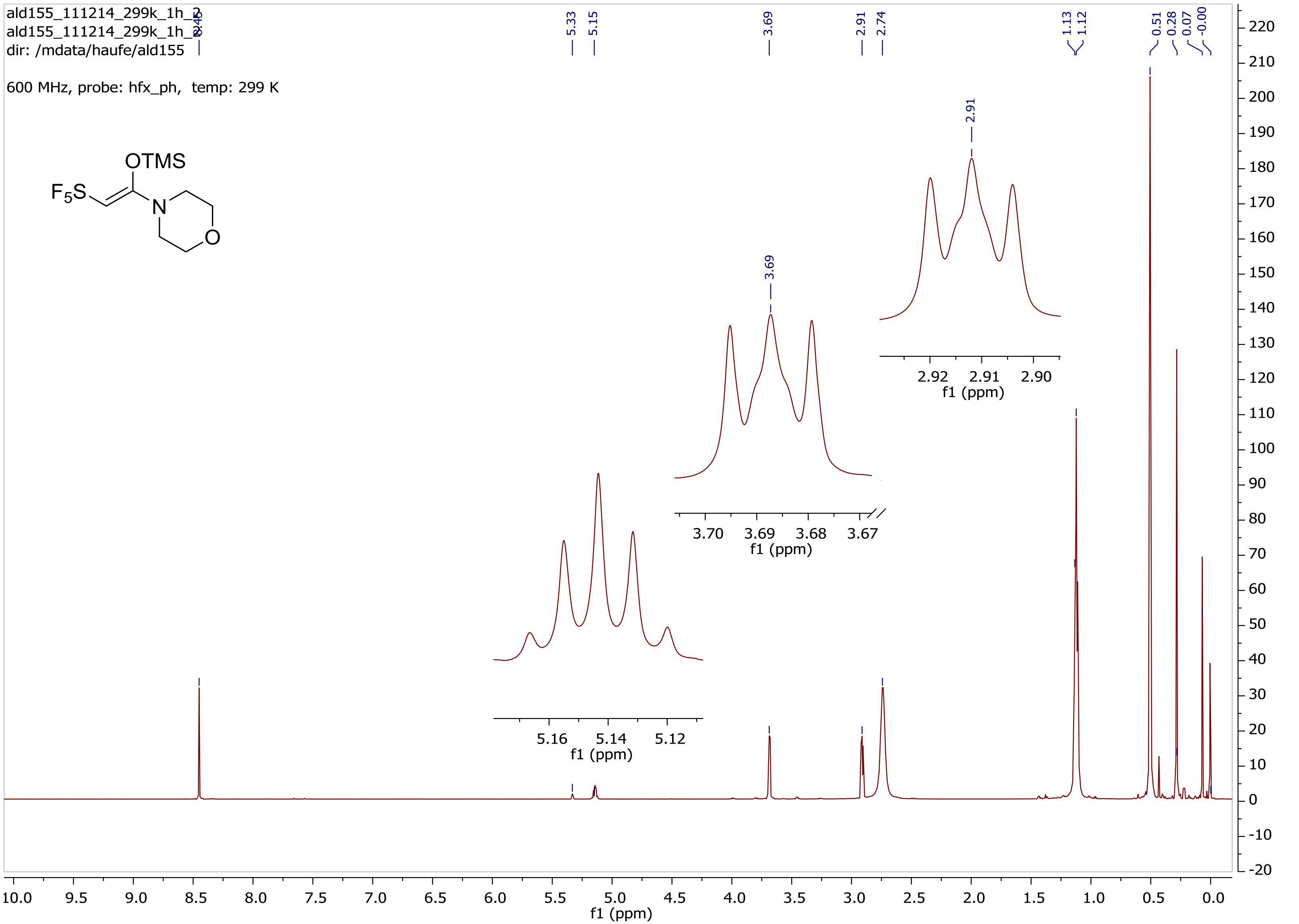

^{13}C NMR spectrum of the (*Z*)-ketene aminal formed from compound **8** in CD_2Cl_2 (crude)

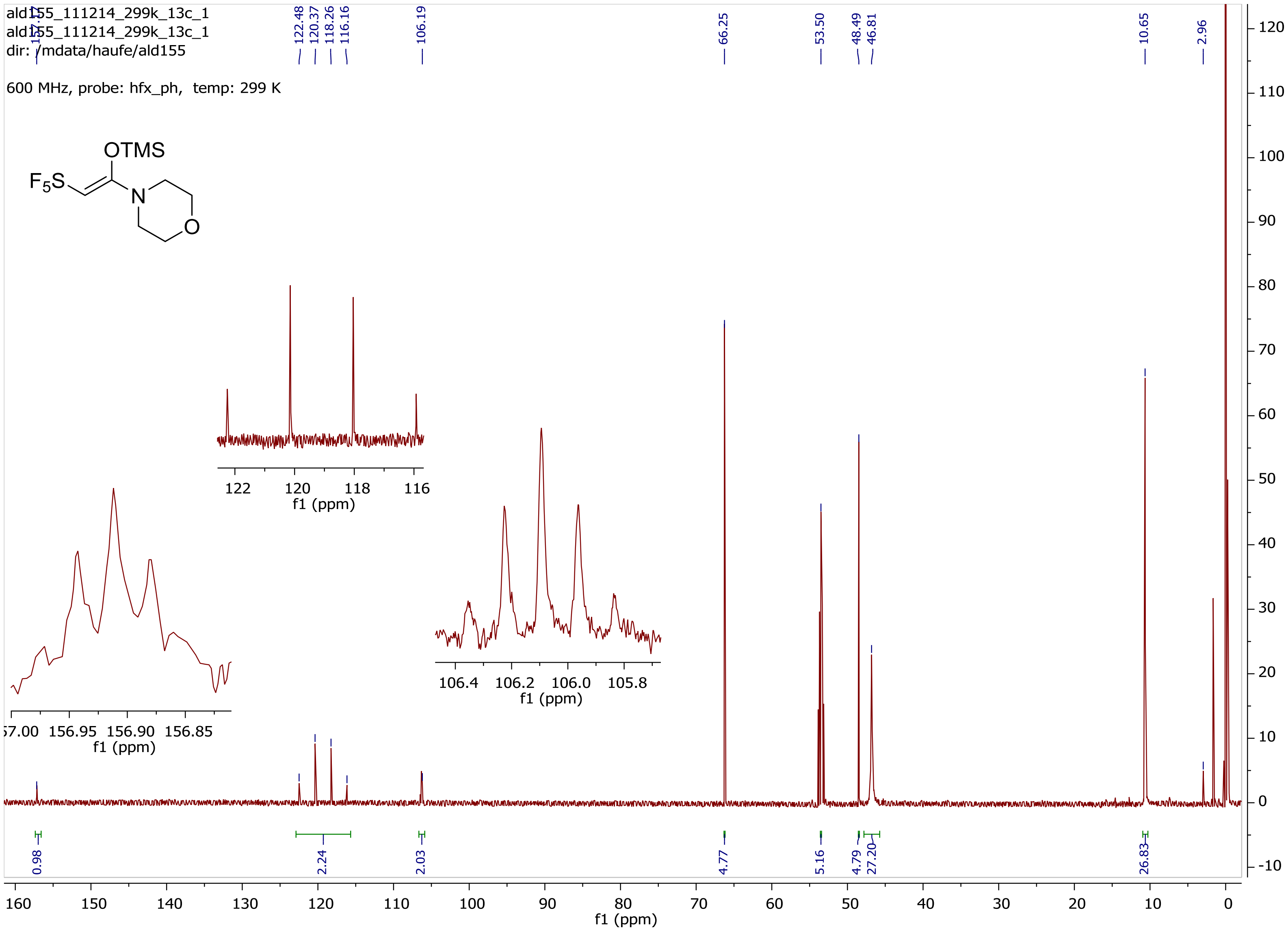

^{19}F NMR spectrum of the (*Z*)-ketene aminal formed from compound **8** in CD_2Cl_2 (crude, positive part)

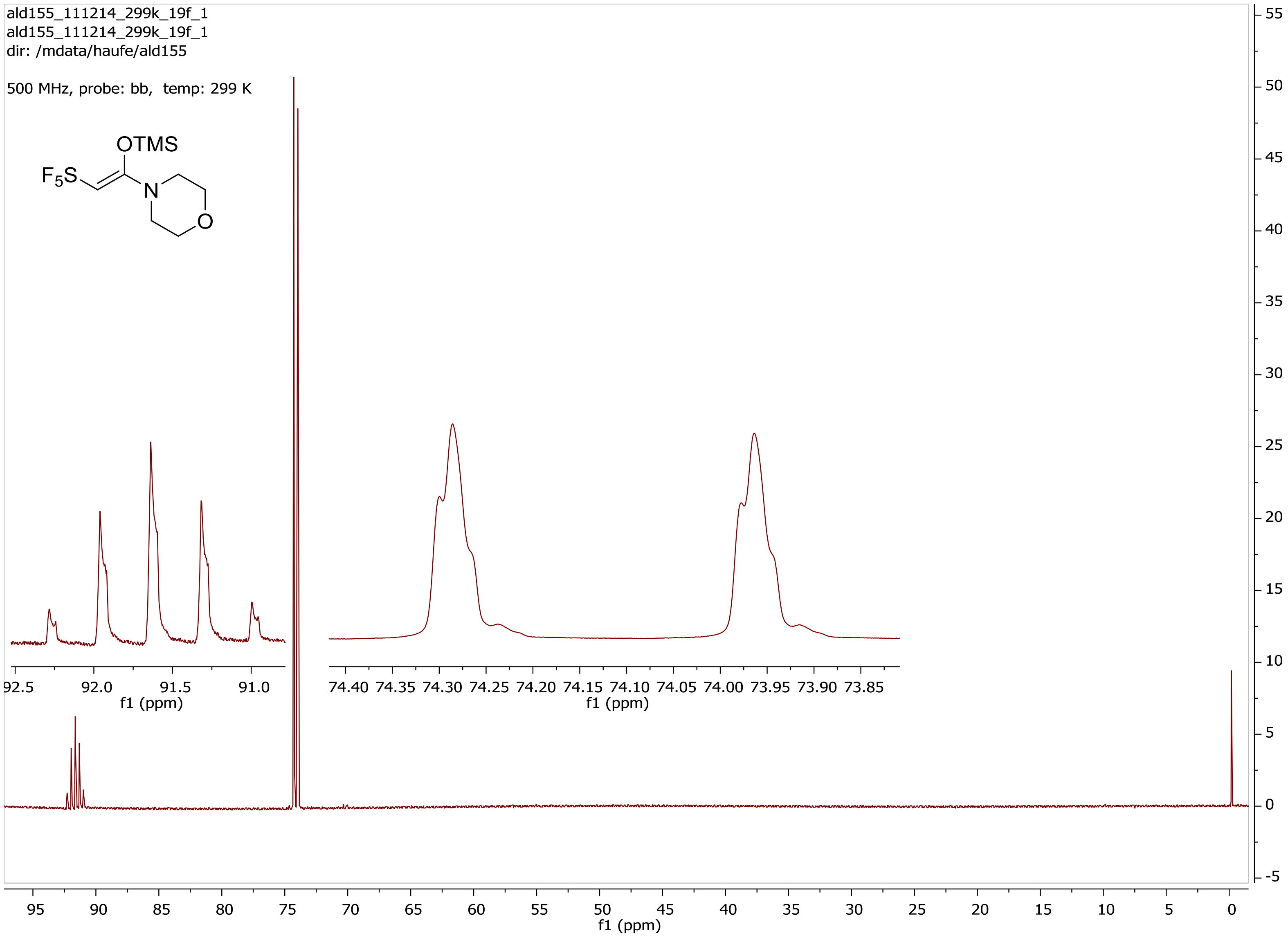

$^{1}H,^{19}F$-correlation spectrum (gHOESY) of the (*Z*)-ketene aminal formed from compound **8**

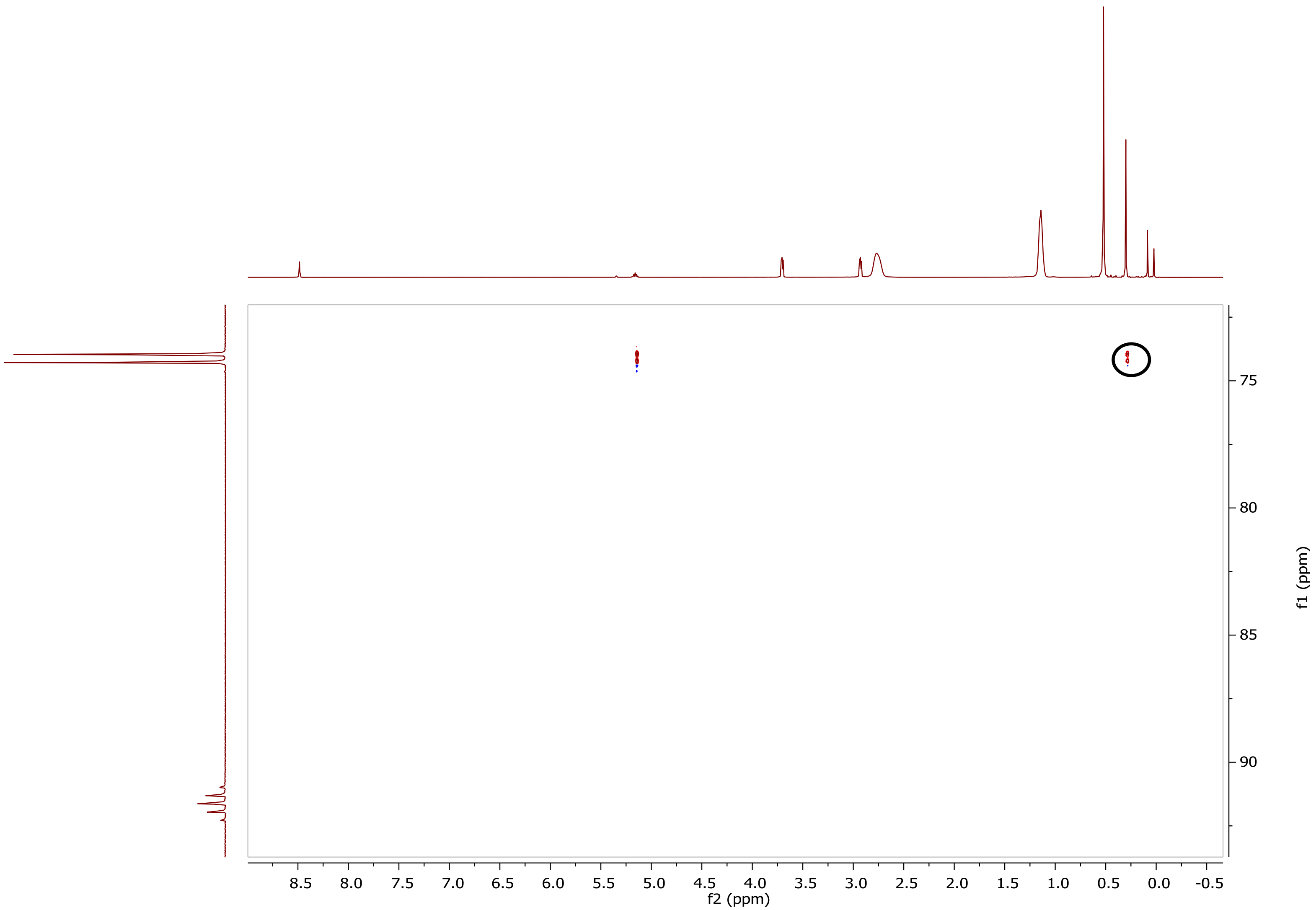

^{1}H NMR spectrum after workup of the reaction of (*Z*)-ketene aminal of **8** with *p*-nitrobenzaldehyde (crude)

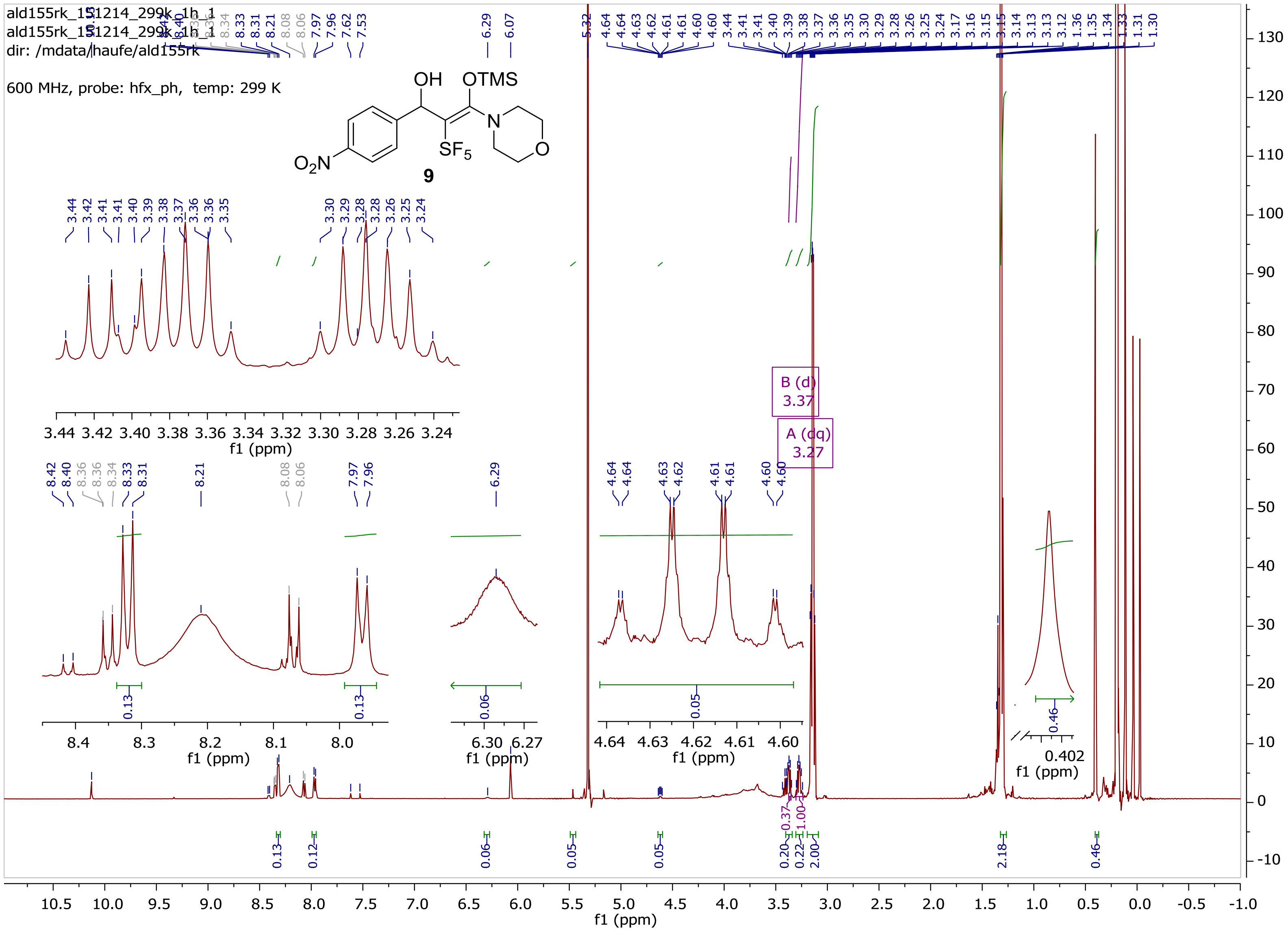

^{19}F NMR spectrum before workup of the reaction of (*Z*)-ketene aminal of **8** with *p*-nitrobenzaldehyde (crude)

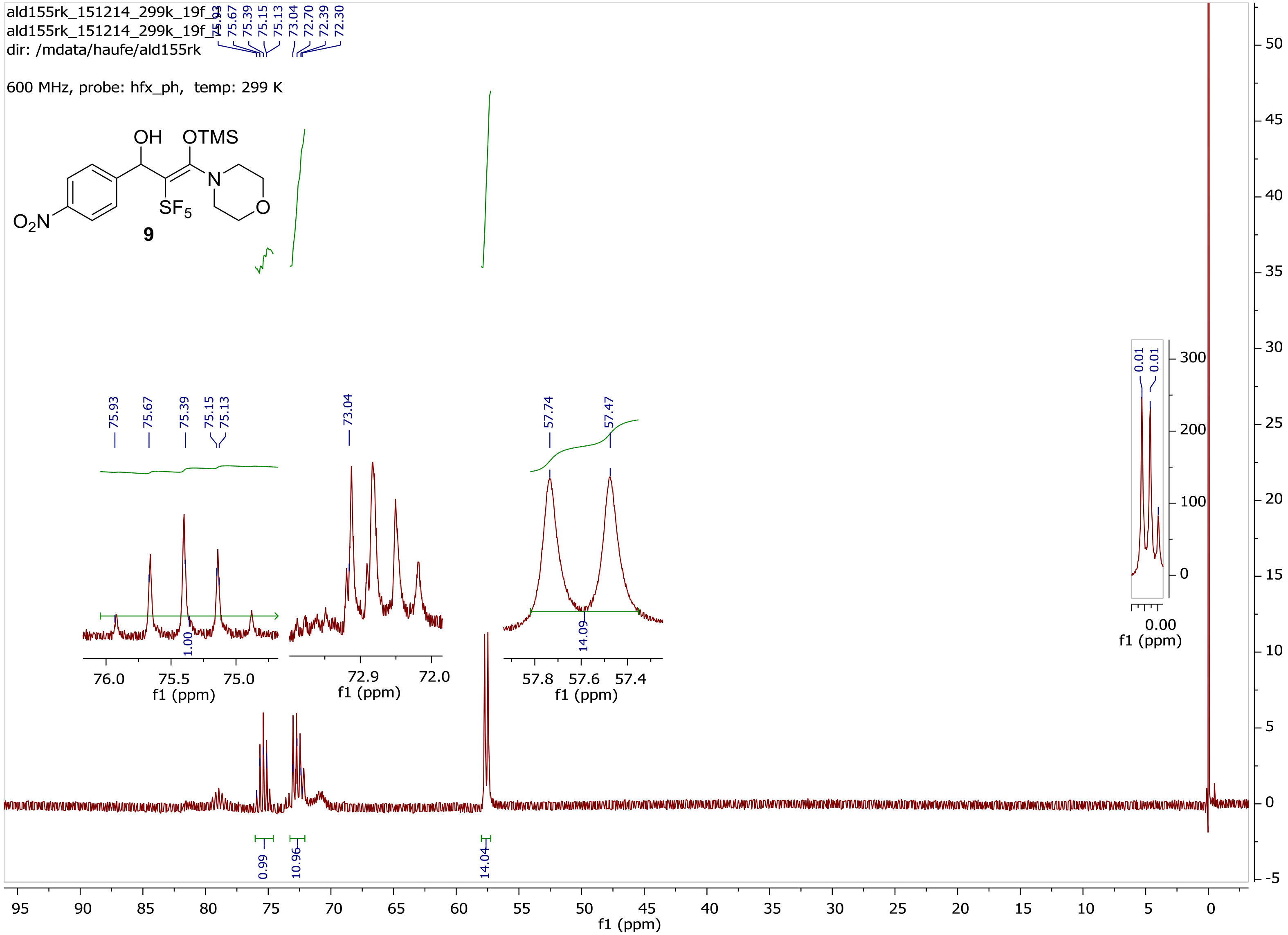

Supplement: File 1 — General procedure, synthesis of the aldol products, spectroscopic data, and copies of 1H, 13C, and 19F NMR spectra. [file Beilstein_J_Org_Chem-14-373-s001.pdf]
